# Genetic Predisposition to Sporadic Congenital Hearing Loss in a Pediatric Population

**DOI:** 10.1038/srep45973

**Published:** 2017-04-06

**Authors:** Jinsei Jung, Joon Suk Lee, Kyeong Jee Cho, Seyoung Yu, Joo-Heon Yoon, Heon Yung Gee, Jae Young Choi

**Affiliations:** 1 Department of Otorhinolaryngology, Brain Korea 21 PLUS Project for Medical Sciences, Yonsei University College of Medicine, Seoul 03722, Korea; 2 Department of Pharmacology, Brain Korea 21 PLUS Project for Medical Sciences, Yonsei University College of Medicine, Seoul 03722, Korea

## Abstract

Discriminating between inherited and non-inherited sporadic hearing loss is challenging. Here, we attempted to delineate genetic inheritance in simplex cases of severe-to-profound congenital hearing loss in Korean children. Variations in *SLC26A4* and *GJB2* in 28 children with bilateral severe-to-profound non-syndromic hearing loss (NSHL) without familial history were analyzed using Sanger sequencing. Genetic analysis of individuals without mutations in *SLC26A4* and *GJB2* was performed by whole exome sequencing (WES). Bi-allelic mutations in *SLC26A4* and *GJB2* were identified in 12 and 3 subjects, respectively. Of the 13 individuals without mutations in *SLC26A4* and *GJB2*, 2 and 1 carried compound heterozygous mutations in *MYO15A* and *CDH23*, respectively. Thus, 64.3% (18/28) of individuals with NSHL were determined to be genetically predisposed. Individuals with sporadic severe-to-profound NSHL were found to mostly exhibit an autosomal recessive inheritance pattern. Novel causative candidate genes for NSHL were identified by analysis of WES data of 10 families without mutations in known causative genes. Bi-allelic mutations predisposing to NSHL were identified in 64.3% of subjects with sporadic severe-to-profound NSHL. Given that several causative genes for NSHL are still unidentified, genetic inheritance of sporadic congenital hearing loss could be more common than that indicated by our results.

Hearing loss is a common sensorial disorder, with an incidence of 1 in 500–1000 among children^1^. At least half of these cases are attributable to genetic factors, and more than two-third of the cases in this subset are classified as non-syndromic hearing loss (NSHL)^2^, which is associated with autosomal dominant (AD) and recessive (AR), X-linked, and maternal inheritance patterns. Of the more than 100 genes (>140 loci) associated with NSHL, approximately 70% contribute to AR-NSHL (http://hereditaryhearingloss.org/)^3^.

Most individuals with AR-NSHL experience severe-to-profound congenital hearing loss^3^,^4^. Severe-to-profound congenital or prelingual hearing loss results in the delay of language and behavioural development at an early age^5^. Therefore, early diagnosis of hearing loss should be mandatory in screening procedures such as newborn hearing screening. Profound congenital hearing impairment detected in newborn screening is usually sporadic^6^. Over 90% of newborns with this condition are born of parents with normal hearing^7^, who will require genetic counseling for hearing rehabilitation and future family planning. Unfortunately, the genetic characteristics of individuals with sporadic hearing loss have not been sufficiently investigated till date^6^. Specifically, sporadic cases of severe-to-profound congenital hearing loss have been rarely weighted in previous targeted exon sequencing (TES) analyses. Moreover, genetic contribution is so heterogeneous that diversity among individuals of different ethnicities is great. For example, the most common genetic cause of hearing loss among Caucasians is *GJB2* mutation, whereas that among East Asians is *SLC26A4* mutation^8^,^9^. Therefore, it is necessary to delineate the characteristics of genetic predisposition patterns according to ethnicity.

Several studies have employed TES for identifying causative mutations of hearing loss^6^,^10^,^11^,^12^,^13^. Whole exome sequencing (WES) has also been successfully used for analysis of genetic factors for hearing loss^5^,^14^, Additionally, WES has facilitated the identification of novel genes associated with Mendelian disorders including hearing loss^15^,^16^,^17^. Recent studies have identified several causative genes for NSHL, including *GPSM2, DNMT1, ELMOD3, GRXCR2*, and *ADCY1*, by WES^15^,^16^,^18^,^19^,^20^.

In the present study, we aimed to establish a molecular diagnosis of sporadic congenital hearing loss in 28 individuals with severe-to-profound congenital NSHL (auditory hearing threshold >70 dB nHL) by genetic analysis by Sanger sequencing and WES.

## Methods

### Subjects

This study was approved by the institutional review board of the Severance Hospital, Yonsei University Health System (IRB#4-2015-0659). After obtaining informed consent, individuals with hearing loss were enrolled in the Yonsei University Hearing Loss (YUHL) cohort, and their clinical and pedigree data were recorded. The experimental methods were performed in accordance with the approved guidelines. For children, written informed consent was obtained from the parents. From the YUHL cohort, 28 individuals with bilateral severe-to-profound hearing loss without familial history were selected for this study, which was conducted between January 2013 and December 2015 at the Department of Otorhinolaryngology, Severance Hospital, Seoul, Korea. The selected individuals exhibited no other syndromic features except hearing loss and were referred to the Severance Hospital for cochlear implantation. Individuals with a history of non-inherited risk factors for hearing loss, such as perinatal cytomegalovirus infection, neonatal intensive care unit treatment, and middle ear infection, were excluded.

### Diagnosis of hearing loss

Physical examination and otoscopy were administered by an otologist. All subjects underwent evaluation for auditory brainstem response (ABR), auditory steady state response (ASSR), and oto-acoustic emission (OAE) for diagnosis of hearing impairment. Subjects who exhibited thresholds <70 dB nHL for ABR were excluded. Average thresholds for ASSR were measured at frequencies of 0.5, 1, 2, and 4 kHz. None of the subjects suspicious for auditory neuropathy were unresponsive in ABR evaluation and presented normal OAE findings. All participants underwent temporal bone computed tomography (TBCT) and magnetic resonance imaging for evaluation of inner ear and temporal bone anomalies. In subjects with unilateral or bilateral enlarged vestibular aqueduct (EVA), all of the exons of *SLC26A4* were sequenced using direct Sanger sequencing. In subjects without EVA, *GJB2* was sequenced using direct Sanger sequencing. The primer sequences for *SLC26A4* and *GJB2* are listed in Table S1. The genotypes of individuals with no causative mutations in *SLC26A4* or *GJB2* were analyzed using WES. In case of subjects with cochlear implants, audiological outcomes at 6 months post-surgery were measured in terms of auditory performance (CAP) score and age equivalence based on the sequenced language scale for infants (SELSI)^21^,^22^.

### DNA preparation, whole exome sequencing, sequence alignment, and variant calling

Whole blood (3 ml) was collected from the participants and, when available, their siblings and parents for segregation analysis. Genomic DNA was extracted from peripheral leukocytes using red blood cell and cell lysis solutions and a protein precipitation solution (QIAGEN). Whole exome capture was performed using the Agilent SureSelect V5 enrichment capture kit (Agilent Technologies). The enriched library was then sequenced using the HiSeq 2500 sequencing system (Illumina; 101-base paired-end sequencing). Image analysis and base calling were performed with the Pipeline software (Illumina) using default parameters. Sequence reads were mapped to the human reference genome assembly (GRCh37/hg19) using the CLC Genomic Workbench (version 9.0.1) software (QIAGEN). Mapping was performed using the “Map Reads to Reference” function of the CLC Genomic Workbench software with the following settings: mismatch cost, 2; insertion cost, 3; deletion cost, 3; length fraction, 0.5; similarity fraction, 0.9; and map to nonspecific reads, random. Nonspecific reads were ignored for count and coverage. All variants with a minimum coverage of 2 were called using the “Basic Variant Caller” function of the CLC Genomic Workbench and annotated.

### Filtering and evaluation of variants

Variant ranking and calling for disease-causing mutations was performed according to the accepted standard in molecular diagnostics^23^,^24^. The variant filtering process is described in Fig. S1 and Table S2. In the first step, variants with minor allele frequencies >1% in the single nucleotide polymorphism (dbSNP; version 138) or 1000 genomes (2504 individuals; phase 3 data) databases were excluded. In the second step, variants present in the homozygous or hemizygous state in 32 healthy Korean individuals without hearing loss (internal control WES data) were excluded. In step 3, synonymous variants and intronic variants not located within splice site regions were excluded. In step 4, variants of all 72 genes known to be monogenic factors for NSHL were systematically evaluated^25^. In step 5, if there were no possible causative variant, a recessive inheritance pattern was assumed on the basis of the pedigree analysis results. Therefore, homozygous, bi-allelic, and de novo heterozygous variants were retained, while single heterozygous variants, except for de novo variants, were excluded from further evaluation. In case of male participants with hearing loss, hemizygous variants were also considered. In the final step, the remaining variants were ranked based on conservation of the mutated amino acid residue across species and their probable impact on the function of the encoded protein. Mutation calling was performed by geneticists and cell biologists with knowledge of clinical phenotypes and pedigree structure and experience with WES analysis. The remaining variants were confirmed in the original participant DNA samples by Sanger sequencing. Segregation analysis was performed whenever parental DNA was available.

### Copy-number variant (CNV) analysis

Analysis of CNV was performed using the paired-end WES data using the EXCAVATOR version 2.2^26^ and ExomeDepth version 1.1.10^27^ tools with default settings. The GRCh37/hg19 database was used as the reference assembly for calculation of GC content. The WES dataset of 32 internal control subjects was compared with that of the study participants. Copy number variations at specific target regions were estimated according to different CNV detection algorithms using the Agilent SureSelect V5 kit.

## Results

### Family recruitment and clinical assessment

**Figure 1:**
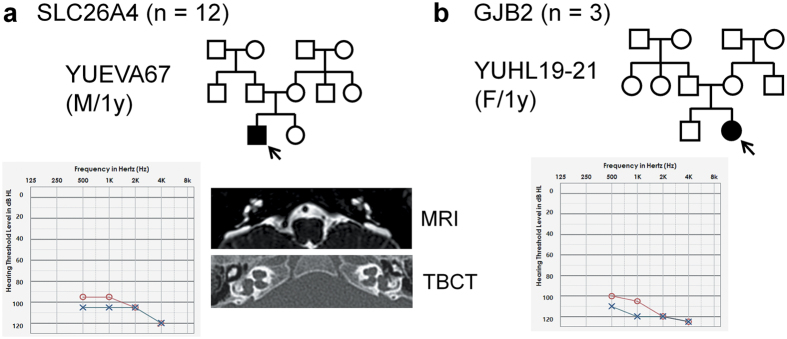
Family pedigree and audiological phenotype of families with causative mutations in *SLC26A4* or *GJB2*. (**a**) The family pedigree of a representative patient with sporadic congenital hearing loss (YUEVA67) from among twelve patients with bi-allelic mutations of *SLC26A4* is shown. The estimated thresholds for auditory steady-state response (ASSR) are >90 dB HL at 500, 1000, 2000, and 4000 Hz (red color, right ear; blue color, left ear). Internal auditory canal magnetic resonance imaging and temporal bone computed tomography images reveal bilateral enlarged vestibular aqueduct and endolymphatic sac. (**b**) The family pedigree and ASSR findings of a representative patient with sporadic congenital hearing loss (YUHL19-21) from among three patients with bi-allelic mutations of *GJB2* are depicted. YUHL, Yonsei University hearing loss; YUEVA, Yonsei University enlarged vestibular aqueduct.

This study included 28 unrelated children (male, 15; female, 13; mean age, 2.6 ± 1.8 years; age range, 8–70 months) with sporadic severe-to-profound NSHL and without syndromic features or familial history of hearing impairment. The average thresholds for ASSR in the right and left ears were 103.0 ± 24.5 dB and 97.4 ± 23.0 dB, respectively. Of the 28 children, 12 exhibited EVA and/or Mondini’s deformity (cochlear anomaly where the cochlea has only 1.5 turns), representing DFNB4 hearing impairment with bi-allelic mutations in *SLC26A4* (Fig. 1a). Direct Sanger sequencing for all of the exons of *SLC26A4* revealed bi-allelic mutations in *SLC26A4* in all 12 of these subjects (Table 1); one of the subjects, Yonsei University enlarged vestibular aqueduct (YUEVA)-65, exhibited a novel variant—a nonsense mutation of p.Y27*. Apart from EVA and/or Mondini’s deformity, these subjects exhibited no other anatomical inner/middle ear anomalies. Direct Sanger sequencing for *GJB2* in the 16 children without EVA revealed pathogenic bi-allelic mutations in *GJB2* in 3 subjects (Fig. 1b and Table 1).

### Identification of causative mutations by whole exome sequencing

The genetic characteristics of 13 individuals without causative mutations in *GJB2* and *SLC26A4* were further analyzed using WES. On an average, the WES datasets of each of the 13 individuals had 99.2%, 97.7%, and 92.4% of mappable bases represented by coverage of at least 1, 10, and 20 reads, respectively. The mean exome coverage was 71.6.

The 72 known NSHL-associated genes were first examined for the presence of variants (Table S3)^25^. Whole exome sequencing of these genes achieved an average coverage of 65.8 (Table S4). Variants that existed in homozygous or hemizygous states in 32 healthy Korean control subjects were excluded, as were common variants with minor allele frequencies >1%, as described in Materials and Methods. We also examined the most recent version of dbSNP (http://www.ncbi.nlm.nih.gov/SNP/), exome variant server (EVS; http://evs.gs.washington.edu/EVS/), exome aggregation consortium (ExAC; http://exac.broadinstitute.org/), and the National Biobank of Korea control databases (NBK) to evaluate the filtered variants. The remaining variants had minor allele frequencies <0.01, and were ranked based on conservation of the affected amino acid residue and the predicted pathogenicity. In addition, we investigated whether the surviving variants had minor allele frequencies <0.005 and 0.0005 for AR and AD genes, respectively; these thresholds were consistent with the minor allele frequency thresholds that Shearer *et al*. suggested for pathologic variants in NSHL^28^.

Compound-heterozygous mutations were identified in two known AR NSHL-linked genes, *MYO15A* (YUHL8-21 and YUHL13-21) and *CDH23* (YUHL24-21), in three individuals with NSHL; in two of these families, where parental DNA available, segregation was confirmed by Sanger sequencing (Table 2 and Figs S2, S3). Thus, NSHL in these three individuals most likely resulted from these recessive mutations.

Two individuals with profound congenital NSHL exhibited variants in two AD genes, *MYO7A* (YUHL26-21) and *DFNA5* (YUHL44-21) (Table S5). Parental DNA was available in both cases, and segregation analysis by Sanger sequencing revealed that the unaffected mothers (YUHL26-12 and YUHL44-12, respectively) carried the variant in both families (Table S5 and Fig. S4), indicating that these variants were not segregated with the affected status.

As shown in Table S6, heterozygous variants in AR NSHL-linked genes were also identified in six individuals. However, these variants were probably not the causative factors for NSHL in these cases since bi-allelic mutations in AR genes are required to be in trans configuration in order to be causative.

Whole exome sequencing did not cover all 1340 coding exons of the 72 known NSHL-linked genes evaluated in this study. Approximately 2.8% of these exons (38/1340; 17 of 72 genes) were not covered (read depth <1; Table S4). Of the 38 uncovered exons, 24 were not targeted by the Agilent SureSelect V5 kit, while the remaining 14 were targeted but poorly covered. Since seven of the uncovered exons belonged to five AD genes, they were analyzed by Sanger sequencing. However, no causative mutations were identified in these exons in 13 individuals with NSHL. Of the 12 AR genes with uncovered exons, only those with heterozygous mutations detected by WES—*PCDH15* in YUHL14-21, *MYO3A* in YUHL26-21, and *TSPEAR* in YUHL36-21 (Table S6)—were analyzed by Sanger sequencing of the uncovered exons. However, no additional mutations were detected, which left the genetic etiology of NSHL in these individuals unresolved.

### Identification of candidate genes for non-syndromic hearing loss by whole exome sequencing

The WES data of 10 individuals who did not exhibit definite causative mutations in known NSHL-linked genes were analyzed for mutations in monogenic candidate genes. Since NSHL in these 10 individuals was sporadic and had congenital or prelingual onset, and the most frequent mode of inheritance of NSHL-linked mutations is AR inheritance, we assumed the following inheritance patterns: (1) bi-allelic variants in recessive genes, (2) hemizygous variants in X-chromosome genes in affected male subjects, and (3) de novo heterozygous variants in families in which trio analysis was possible. Variant filtering reduced the number of candidate genes to one to 13 in 10 families, as outlined in Fig. S1. In case of YUHL44-21 (Fig. S2c), variant filtering was begun with 170,215 variants from the normal reference sequence. This number was reduced to 929 upon exclusion of homozygous and hemizygous variants in healthy Korean individuals, common variants (minor allele frequencies >1% in public databases), and synonymous variants. Upon considering only those genes with de novo heterozygous variants, hemizygous variants or more than two variants in the same gene, the number of variants was further reduced to 35 variants (28 genes). Exclusion of artefacts by direct inspection of sequence alignment and exclusion of variants with minor allele frequencies <0.005 in public databases left nine variants in eight candidate genes — *HEATR1, SLC25A14, SSX3, ADAMTSL3, CEND1, DOPEY2, SWI5*, and *CAND2*. These variants were subsequently ranked based on their extent of amino acid conservation across species and predicted likelihood to be deleterious for the function of the encoded protein. The WES datasets of other individuals with NSHL in the present study were analyzed in the same manner to identify candidate genes (Table S7). All mutations in novel candidate genes were confirmed by Sanger sequencing of the DNA of the affected individuals and their parents and were found to segregate with the affected status.

In the 10 individuals without causative mutations in known NSHL-linked genes, the EXCAVATOR and ExomeDepth tools detected 126 and 158 CNVs, respectively (Table S8). We specifically focused on deletion or duplication of alleles in an AR pattern and identified a bi-allelic deletion in YUHL10-21 (Figs S2c and S5). The deleted region was located between 30,995,157 and 30,996,641 on chromosome 6 (hg19) and corresponded to a part of the coding sequence of the third exon of *MUC22* (Fig. S5c). This deletion is expected to result in the truncation of the MUC22 protein. However, further study is required to determine whether this deletion is associated with hearing loss.

### Audiological performance after cochlear implantation according to genotype

**Figure 2:**
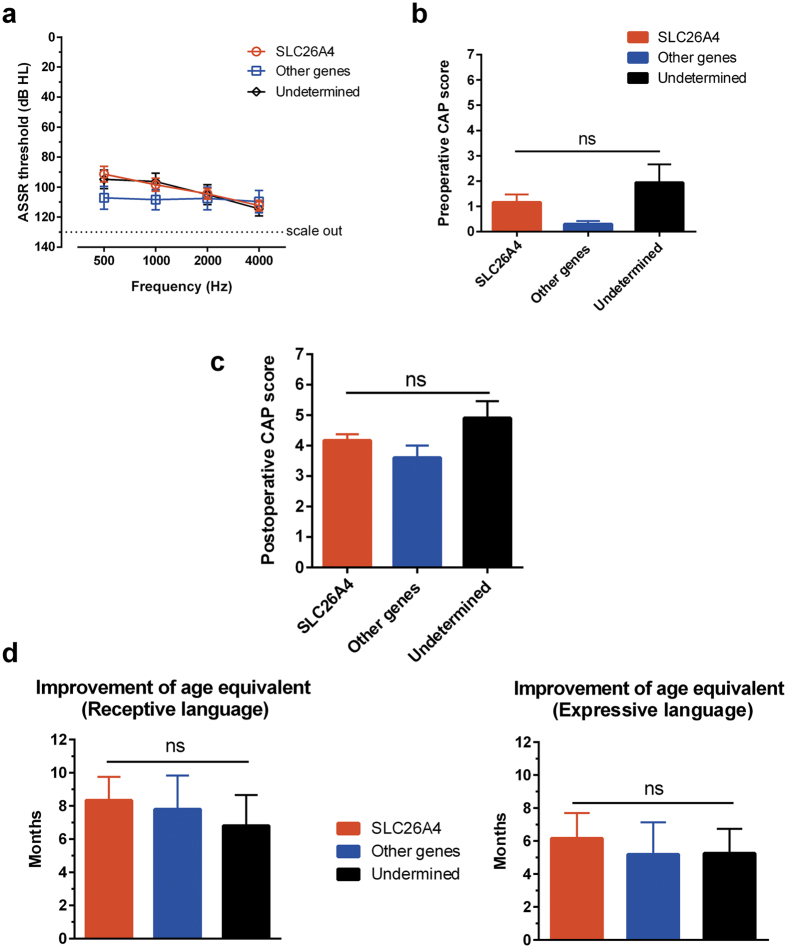
Audiological performance of cochlear implantation. Twenty-five children received cochlear implants. They were sub-divided into three groups—the *SLC26A4* mutation, other gene mutations (including *GJB2, MYO15A*, and *CDH23*), and unidentified etiology groups—according to genotype. (**a**) Preoperative hearing thresholds for auditory steady-state response (ASSR). (**b**) Comparison of preoperative categories of auditory performance (CAP) scores among the three groups. (**c**) Comparison of postoperative CAP scores among the three groups. (**d**) Comparison of improvement in age equivalence in terms of receptive and expressive language among the three groups. ns, not significant (one-way analysis of variance).

Of the 28 subjects included in the present study, 25 received cochlear implants for hearing rehabilitation. The mean age at operation was 26.0 ± 15.8 months. The type of implanted device was CI24RE (Cochlear^TM^, Australia) or Concerto (Med-El^TM^, Austria), depending on the decision of the patients. These 25 subjects were subdivided into three groups—the *SLC26A4* mutation, other gene mutations (including *GJB2, MYO15A*, and *CDH23*), and unidentified etiology groups—according to genotype, and preoperative hearing thresholds for ASSR were compared among these groups (Fig. 2a). Although the group of individuals with mutations in other genes appeared to exhibit comparatively worse hearing thresholds at 500 and 1000 Hz, there were no significant differences in preoperative threshold among the three groups at any of the frequencies (two-way analysis of variance; P > 0.05). All three groups exhibited average preoperative CAP scores <2, where a score of 2 indicated response to spoken signals (e.g., “go” or “boo”). There were no statistically differences in the average preoperative or 6-month postoperative CAP scores among the groups (Fig. 2b and c)^21^. Age equivalence in terms of receptive and expressive language was evaluated according to the SELSI^22^. As shown in Fig. 2d, there were no significant differences in the improvement of receptive or expressive language among the groups.

## Discussion

In the present study, we comprehensively evaluated the contribution of genetic predisposition to severe-to-profound sporadic congenital hearing loss by Sanger sequencing and WES. Of the 28 included subjects, 18 (64.3%) carried pathogenic bi-allelic mutations in 1 of 72 genes known to be associated with NSHL. This diagnostic rate is exceptionally higher compared with that reported in a previous study (37%) involving sporadic NSHL^11^. However, upon exclusion of subjects with *SLC26A4* mutations from the present cohort, the diagnostic rate of NSHL in the present study decreases to 37.5% (6 of 16 subjects), which is comparable to that reported in the previous study^11^.

Interestingly, the frequency of *SLC26A4* mutations in subjects with sporadic congenital hearing loss in the present study is higher compared that reported in previous studies. Pathogenic variants of *SLC26A4* have been previously reported in approximately 10% of cases of severe-to-profound hearing loss in multi-ethnic cohorts^11^,^29^. Similarly, the frequency of *SLC26A4* mutation in Japanese individuals with genetically diagnosed NSHL was reported to be 7.5%, which is comparable to that reported in the two previous studies^30^. In Korea, approximately 15–26% of cases of profound hearing loss were found to be attributable to *SLC26A4* mutations regardless of familial history^9^,^31^. There are several possible reasons for the high frequency of *SLC26A4* mutations observed in the present study. First, the present cohort only comprised individuals with congenital hearing loss, which usually results from genetic defects. Second, genetic evaluation is convincing and easily agreed to by patients in cases where TBCT findings reveal EVA, which might have resulted in patient selection. Nevertheless, the high rate of bi-allelic mutations in patients with sporadic profound hearing loss implies that sporadic congenital hearing loss is caused in a majority of cases in Korea by *SLC26A4* mutations and not by *GJB2* mutations. *GJB2* mutations account for just 9% of the total frequency observed in this study. Therefore, Korean patients should first be evaluated for *SLC26A4* mutations prior to comprehensive genetic analyses such as TES or WES. Because *SLC26A4* comprises 21 exons and requires more than 20 primers for Sanger sequencing, TBCT can be more cost-effective for ruling out *SLC26A4* mutations. Given that the frequency of *SLC26A4* mutations in Korean patients with EVA is >90%, it is reasonable to perform TBCT for discriminating between patients with hearing loss due to *SLC26A4* mutations and those with NSHL due to other AR mutations^9^.

Since the genetic factors associated with hearing loss are extremely heterogeneous, and mutations in nearly 100 genes have been identified as causative factors for NSHL, next-generation sequencing approaches such as TES and WES have been employed for molecular diagnosis of NSHL. Targeted exon sequencing has been used as a cost effective method for genetic diagnosis^5^,^6^,^10^,^11^,^12^,^13^. It provides greater coverage and depth and is less expensive compared with WES. Over the past few years, mutations in several novel genes have been identified as causative factors for NSHL by WES^15^,^16^,^18^,^19^,^20^,^32^,^33^, which highlights the necessity for the updating and optimization of TES for inclusion of these new genes. Considering the over 70 known genes associated with NSHL and the genes that will be identified in the near future, TES might not actually be much cheaper than WES. In this regard, WES has an advantage over TES because the former method can be employed for evaluation of any gene. However, WES also has a few limitations as a diagnostic tool. As shown in Table S4, WES fails to capture certain regions of the exome, which vary depending on the exome capture kit used. In addition, there are mitochondrial genes and microRNAs associated with NSHL^25^ and these are not generally covered by WES. Therefore, WES users need to know the type of capture kit used and consider and resolve any uncovered regions. Improvements in exome capture kits are expected to reduce the extent of uncovered regions with WES.

A previous study involving WES identified causative mutations of NSHL in 60.0% (12 of 20) of the included families^14^, which is higher than the frequency of causative mutations of NSHL observed among individuals evaluated by WES in the present study (23.1%; 3 of 13 individuals). This discrepancy might be attributable to the following factors. First, in the previous study, the authors pre-screened only *GJB2*, which is the most common genetic cause of NSHL in the Middle East; in contrast, the present study involved pre-screening for both *GJB2* and *SLC26A4* mutations. Second, the present cohort only comprised individuals with sporadic hearing loss, whereas the previous study included multiplex cases with at least two affected members. Third, the Korean population is generally outbred, whereas the previous study included only consanguineous families, which exhibit an increased prevalence of AR disorders^34^.

Another study employing WES identified causative mutations of NSHL in 5 of 11 (45.4%) Korean patients with sporadic mild-to-moderate NSHL; the authors also suggested various modes of inheritance, including digenic inheritance (*GRP98*/*PDZ7*)^5^. Similar to the present cohort, the previous study cohort did not include individuals with *GJB2* mutations or EVA. Their reported frequency of causative mutations (45.4%) is higher compared with that observed in the present study (23.1%) despite the fact that mild-to-moderate hearing loss is less frequently diagnosed by molecular methods than severe-to-profound hearing loss. This discrepancy might be attributable to the relatively small sample sizes of both studies. In the present study, we identified two single heterozygous variants in two AR genes in three families — YUHL13-21, YUHL20-21, and YUHL44-21 (Table S6). Among these families, YUHL13-21 exhibited heterozygous variants in *GIPC3* and *LOXHD1* despite already possessing compound-heterozygous mutations in *MYO15A*. Therefore, the contribution of variants of *GIPC3* and *LOXHD1* to hearing loss is not clear. Heterozygous mutations in YUHL20-21 and YUHL44-21 corresponded to *GRXCR2*/*LOXHD1* and *FAM65B*/*KARS*, respectively. It is not clear whether hearing loss in these cases was a result of digenic inheritance, and it is difficult to prove this hypothesis without any experimental evidence. Incidental findings of heterozygous variants in known NSHL genes have also been reported by Diaz-Horta *et al*.^14^.

Two families, YUHL26 and YUHL44, exhibited variants in AD genes (*MYO7A* and *DFNA5*, respectively) that did not segregate with the affected status in the family (Table S5). In both families, the unaffected mothers carried the same variant (Fig. S4), indicating that the variants are not de novo. Incomplete penetrance is observed in AD inheritance; however, it is not clear whether non-segregation in these cases resulted from incomplete penetrance.

Language development after cochlear implantation depends on conditions such as age at implantation, duration of implant use, and preoperative residual hearing^35^. Additionally, genetic predisposition might have an effect on post-implantation outcomes. While a few previous studies have reported better performance in individuals with *GJB2* mutations compared with that in individuals with other genetic etiologies, a few others have demonstrated that individuals with *SLC26A4* mutations exhibit better outcomes than those with an unknown aetiology^36^,^37^,^38^,^39^,^40^. Recently, Wu *et al*. reported that *GJB2* and *SLC26A4* mutations were associated with good long-term post-implant outcomes when implantation was done before the age of 3.5 years. However, we found no significant differences in post-operative auditory outcomes among groups with *SLC26A4* mutations, mutation in other genes, and molecularly undetermined etiology, which might be because the present study only included short-term follow-up data. Thus, long-term outcomes may differ according to the genotype. Nevertheless, it is noteworthy that preoperative residual hearing was uniform across all groups in the present study (Fig. 2a). In previous studies that reported better post-implant outcomes in individuals with *SLC26A4* mutations as well, preoperative hearing and auditory function in individuals with *SLC26A4* mutations were better compared to those in control subjects^36^,^40^. This indicates that postoperative outcomes correlate with preoperative hearing function, and comparison between groups with dissimilar preoperative hearing functions is inappropriate. Thus, it is reasonable that, among individuals with similar preoperative hearing thresholds, specific genetic etiologies—at least, *GJB2* or *SLC26A4* mutations—do not result in better or worse postoperative auditory outcomes.

Sanger sequencing of top priority genes according to ethnicity renders the genetic evaluation process relatively cost-effective and simple. With the exception of *SLC26A4* and *GJB2* mutations, mutations in genes linked to hearing loss are extremely rare and can be effectively evaluated by WES^31^. Our findings indicate that direct sequencing of *SLC26A4* and *GJB2* followed by WES is a simple and precise protocol for molecular diagnosis of sporadic NSHL in a pediatric population.

## Additional Information

**How to cite this article:** Jung, J. *et al*. Genetic Predisposition to Sporadic Congenital Hearing Loss in a Pediatric Population. *Sci. Rep.* **7**, 45973; doi: 10.1038/srep45973 (2017).

**Publisher's note:** Springer Nature remains neutral with regard to jurisdictional claims in published maps and institutional affiliations.

## Supplementary Material

Supplementary Figures and Tables

## Figures and Tables

**Table 1:** Bi-allelic mutations of *SLC26A4* and *GJB2* in 15 families with sporadic congenital hearing loss.

Patient	Sex	Age	Gene	Genomic change	Protein change	Location	Mutation type	HGMD
YUEVA 38	M	4y	*SLC26A4*	c.2168 A > G	p.His723Arg	Exon 19	Missense	yes
				c.439 A > G	p.Met147Val	Exon 5	Missense	yes
YUEVA 59	M	2y	*SLC26A4*	c.2168 A > G	p.His723Arg	Exon 19	Missense	yes
				c.919-2 A > G	Splicing	Intron 7	Splicing variant	yes
YUEVA 60	M	3y	*SLC26A4*	c.2027_2028insT	p.Arg677Alafs*11	Exon 17	Insertion	yes
				c.2168 A > G	p.His723Arg	Exon 19	Missense	yes
YUEVA 61	F	2y	*SLC26A4*	c.2168 A > G	p.His723Arg	Exon 19	Missense	yes
				c.919-2 A > G	Splicing	Intron 7	Splicing variant	yes
YUEVA 65	M	2y	*SLC26A4*	c.1229 C > T	p.Thr410Met	Exon 10	Missense	yes
				c.81 C > A	p.Tyr27*	Exon 2	Nonsense	no
YUEVA 67	M	11 m	*SLC26A4*	c.2168 A > G	p.His723Arg	Exon 19	Missense	yes
				c.919-2 A > G	Splicing	Intron 7	Splicing variant	yes
YUEVA 69	M	2y	*SLC26A4*	c.2168 A > G	p.His723Arg	Exon 19	Missense	yes
				c.439 A > G	p.Met147Val	Exon 5	Missense	yes
YUEVA 72	M	4y	*SLC26A4*	c.2168 A > G	p.His723Arg	Exon 19	Missense	yes
				c.919-2 A > G	Splicing	Intron 7	Splicing variant	yes
YUEVA 73	M	2y	*SLC26A4*	c.2168 A > G	p.His723Arg	Exon 19	Missense	yes
				c.439 A > G	p.Met147Val	Exon 5	Missense	yes
YUEVA 74	F	2y	*SLC26A4*	c.2168 A > G	p.His723Arg	Exon 19	Missense	yes
				c.2168 A > G	p.His723Arg	Exon 19	Missense	yes
YUEVA 77	F	1y	*SLC26A4*	c.2168 A > G	p.His723Arg	Exon 19	Missense	yes
				c.2168 A > G	p.His723Arg	Exon 19	Missense	yes
YUEVA 111	M	4y	*SLC26A4*	c.916_917insG	p.Val306Glyfs*24	Exon 7	Insertion	yes
				c.919-2 A > G	Splicing	Intron 7	Splicing variant	yes
YUHL 6–21	F	5y	*GJB2*	c.605_606insAGAAG ACTGTCTTCACAG TGTTCATGATTGC AGTGTCTGGAATTTG	p.Cys202*	Exon2	Inserion	yes
				c.9 G > A	p.Trp3*	Exon2	Nonsense	yes
YUHL 11–21	M	10 m	*GJB2*	c.235delC	p.Leu79Cysfs*3	Exon2	Deletion	yes
				c.427 C > T	p.Arg143Trp	Exon2	Missense	yes
YUHL 19–21	F	11 m	*GJB2*	c.299_300delAT	p.His100Rfs*14	Exon2	Deletion	yes
				c.427 C > T	p.Arg143Trp	Exon2	Missense	yes

YUHL, Yonsei University hearing loss; YUEVA, Yonsei University enlarged vestibular aqueduct.

**Table 2:** Causative mutations detected by WES in three individuals with NSHL.

Gene Symbol	Indiv-idual	Sex	Age of onset	Nucleotide change^a^	Amino acid change	Exon (zygosity, segrega-tion)	Amino acid sequence conservation^b^	Frequencies in the dbSNP database^c^	Frequencies in the ExAC database^d^	Frequencies in the NBK database^e^	PP2^f^	MT^g^	PRO-VEAN^h^	SIFT^i^
*MYO15A*	YUHL 8–21	Fm	2 yr	c.7990 C > A	p.Pro2664Thr	42 (het, ND)	G. *gallus*	rs577657134 (MAF: A = 0.0002/1) rs564438028	12/118432 (no hom)	A = 0.00377834	Bn (0.026)	PM (0.914)	Del (−2.55)	Dam (0.028)
				c.9221 T > C	p.Met3074Thr	53 (het, ND)	*M. musculus*	(MAF: C = 0.0002/1)	1/120686 (no hom)	ND	Bn0.006	DC(0.952)	Neu (−2.08)	Tol (0.184)
*MYO15A*	YUHL 13–21	Ml	1 yr	c.4322 G > T	p.Gly1441Val	11 (het, F)	*D. rerio *	rs772995303 (NA)	1/116996 (no hom)	ND	PD (1.000)	DC (0.999)	Del (−8.66)	Dam (0.000)
				c.1651 G > A	p.Ala551Thr	1 (het, M)	*M. musculus*	rs747175448 (NA)	ND	ND	BN 0.004	PM (0.999)	Neu (−0.82)	Dam (0.012)
*CDH23*	YUHL 24–21	Fm	1 mo	c.7145 G > A	p.Arg2382Gln	49 (het, F)	*D. rerio *	rs759439688 (MAF: A = 0.00002/3))	3/120700 (no hom)	ND	PD (0.977)	N/	Neu (−0.77)	Dam (0.001)
				c.7361 C > T	p.Thr2454Met	50 (het,ND)	*D. rerio *	rs772949926 (MAF: T = 0.00010/12))	12/120752 (no hom)	ND	PD (0.998)	No	Del (−3.18)	Dam (0.001)

Bn, benign; Dam, damaging; DC, disease causing; Del, deleterious; ExAC, exome aggregation consortium; F, heterozygous mutation identified in father; Fm, female; het, heterozygous in affected individual; M, heterozygous mutation identified in mother; MAF, minor allele frequency; Ml, male; mo, month; NA, not applicable; ND, no data or DNA available; Neu, neutral; NSHL, nonsyndromic hearing loss; PD, probably damaging; PM, polymorphism; PP2, PolyPhen-2 prediction score Humvar; PROVEAN, protein variation effect analyzer; SIFT, sorting intolerant from tolerant; SNP, single nucleotide polymorphism; Tol, tolerant; WES, whole exome sequencing; yr, years; YUHL, Yonsei University hearing loss.

^a^cDNA mutations are numbered according to human cDNA reference sequences NM_016239.3 (*MYO15A*) and NM_022124.5 (*CDH23*); +1 corresponds to A of the translation initiation codon ATG. ^b^Amino acid residue is continually conserved throughout evolution, including in the indicated species. ^c^dbSNP database (http://www.ncbi.nlm.nih.gov/SNP). ^d^ExAC browser (http://exac.broadinstitute.org/). ^e^National Biobank of Korea, Centers for Disease Control and Prevention. ^f^PolyPhen-2 prediction score HumVar ranges from 0 to 1.0; 0 = benign, 1.0 = probably damaging (http://genetics.bwh.harvard.edu/pph2/). ^g^Mutation taster (http://www.mutationtaster.org/). ^h^PROVEAN (http://provean.jcvi.org/index.php). ^h^SIFT (http://sift.jcvi.org/).
