# Supplementary material for: Genetic Predisposition to Sporadic Congenital Hearing Loss in a Pediatric Population
Source: Sci Rep. 2017 Apr 6;7:45973. doi: 10.1038/srep45973 (PMC5382691; doi:10.1038/srep45973)
Supplement: Supplementary Figures and Tables [file srep45973-s1.pdf]

Genetic Predisposition to Sporadic Congenital Hearing Loss in a Pediatric Population

Jinsei Jung[1,†], Joon Suk Lee[2, †], Kyeong Jee Cho[2], Seyoung Yu[2], Joo-Heon Yoon[1], Heon Yung Gee[2], and Jae Young Choi[1]

[1]Department of Otorhinolaryngology, Brain Korea 21 PLUS Project for Medical Sciences, Yonsei University College of Medicine, Seoul 03722, Korea

[2]Department of Pharmacology, Brain Korea 21 PLUS Project for Medical Sciences, Yonsei University College of Medicine, Seoul 03722, Korea

[†]these authors contributed equally to this work

Supplementary Information

Supplementary Figures S1-5

Supplementary Tables S1-8

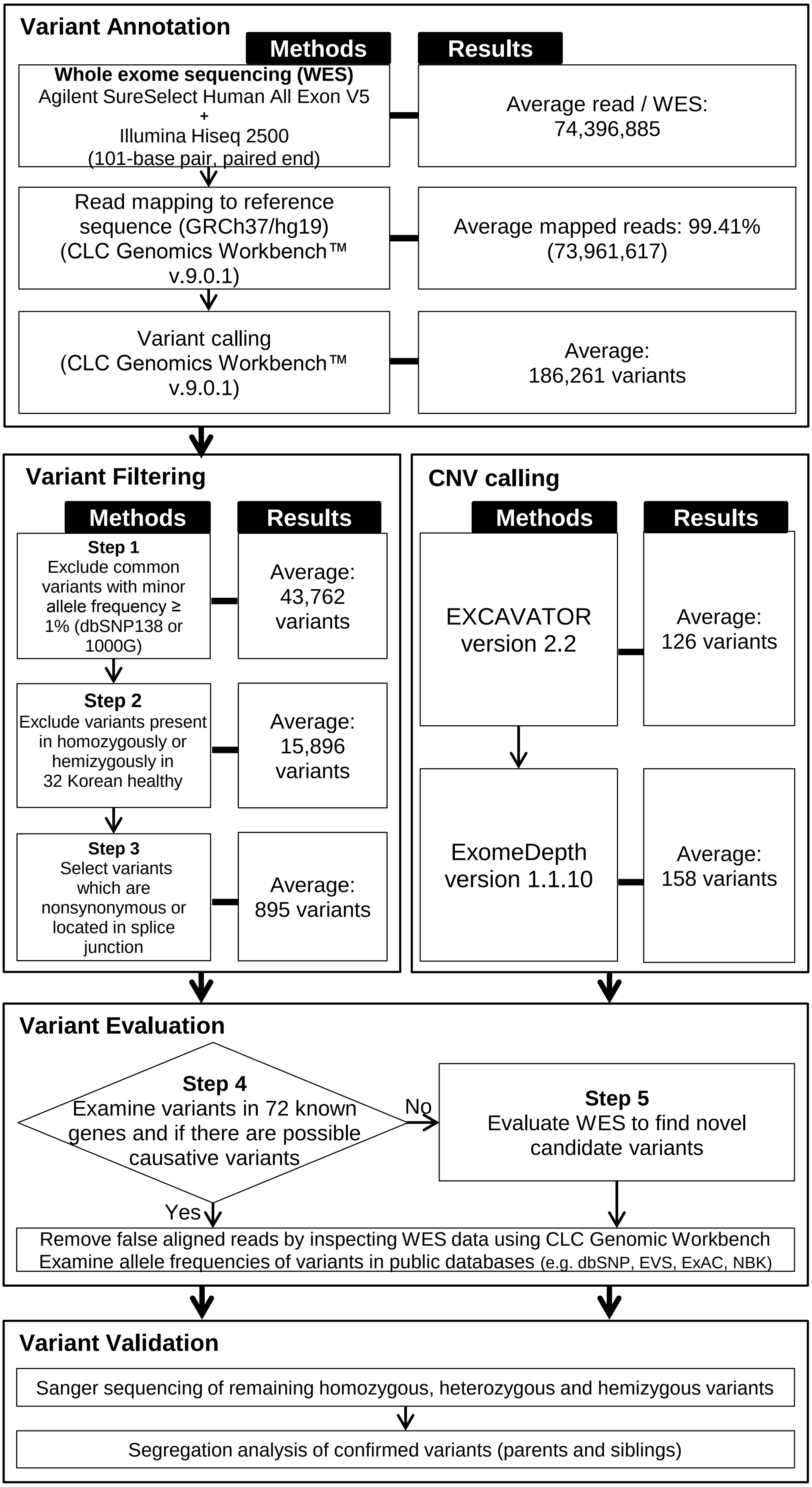

Figure S1. Variant filtering process for the identification of causative mutations.

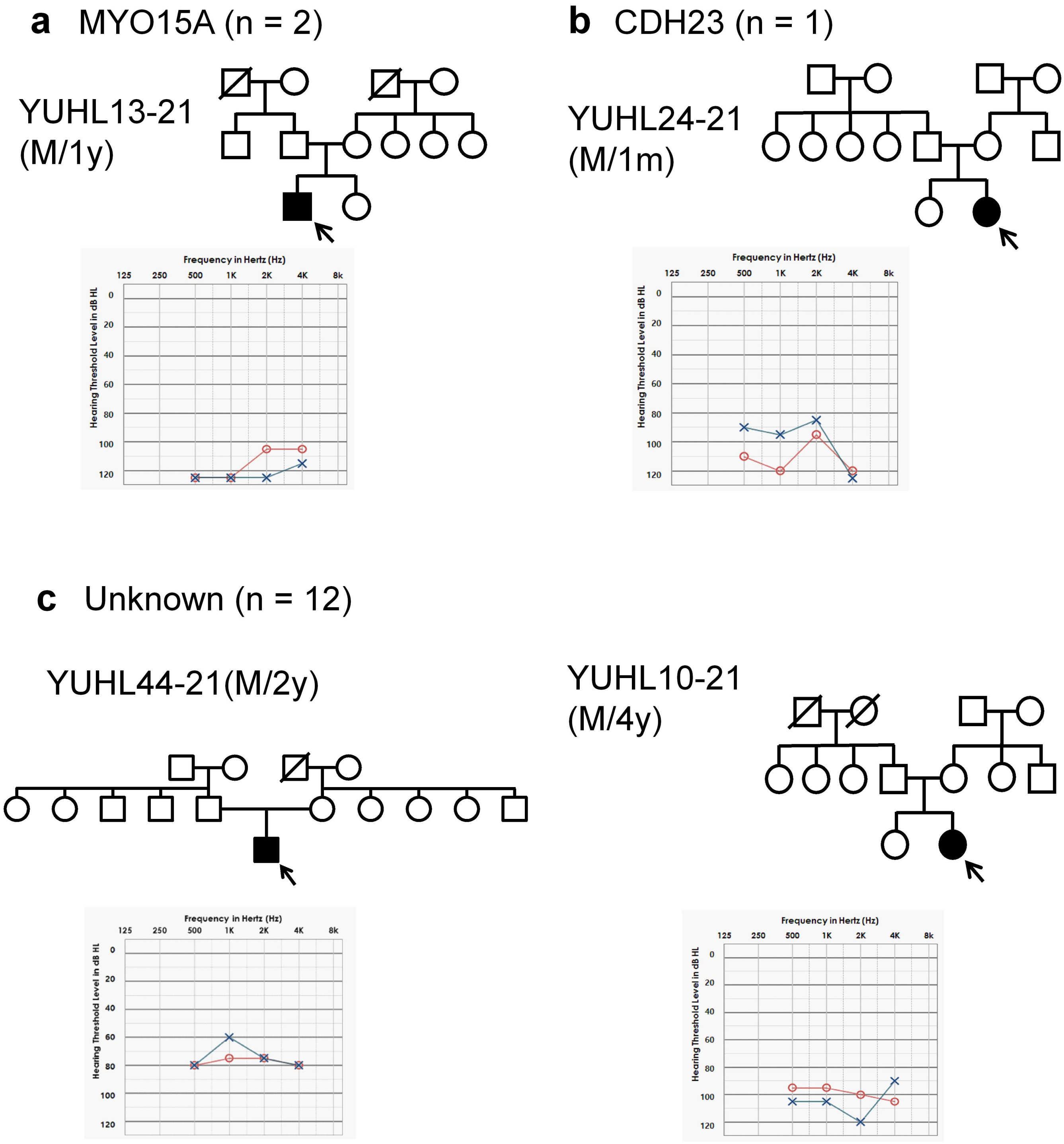

Figure S2. Family pedigree and audiologic phenotype of families included in WES study.

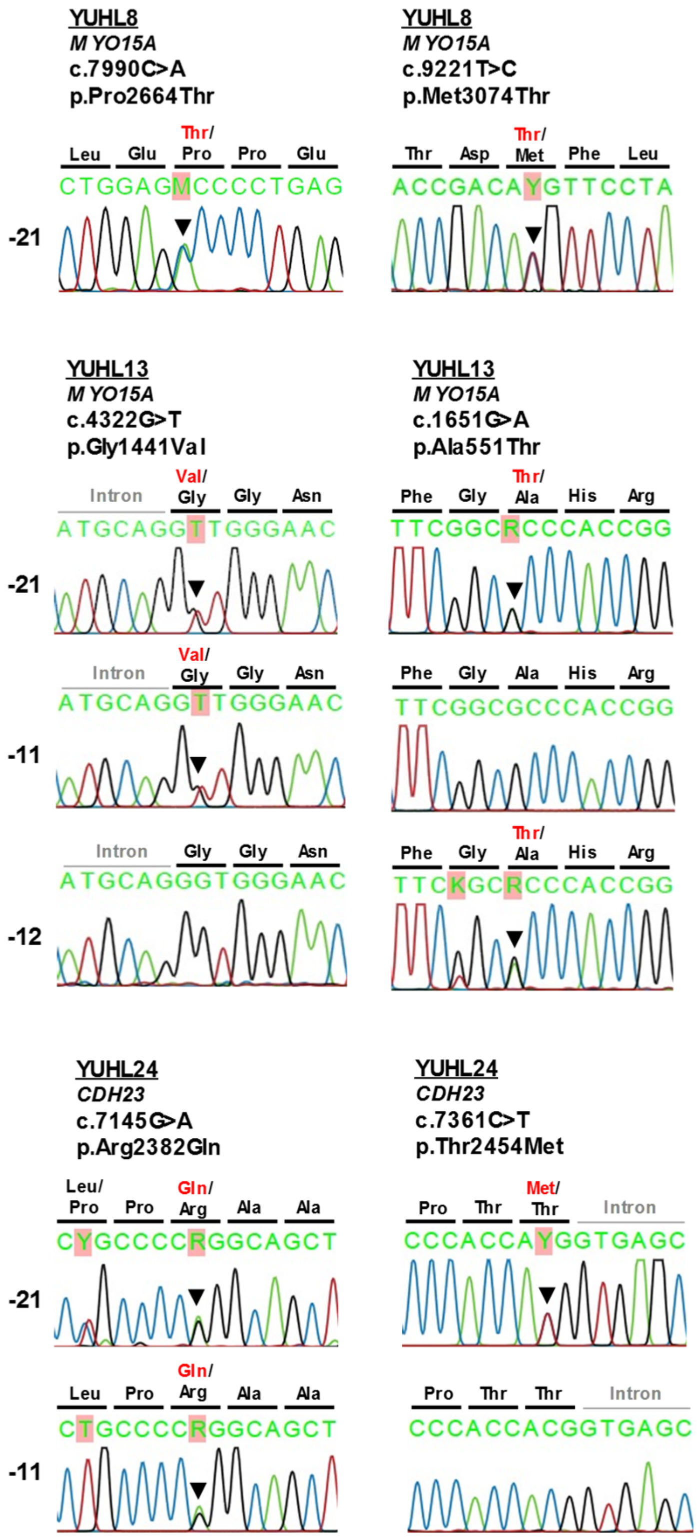

Figure S3. Sequencing traces of mutations detected in *MYO15A* and *CDH23*.

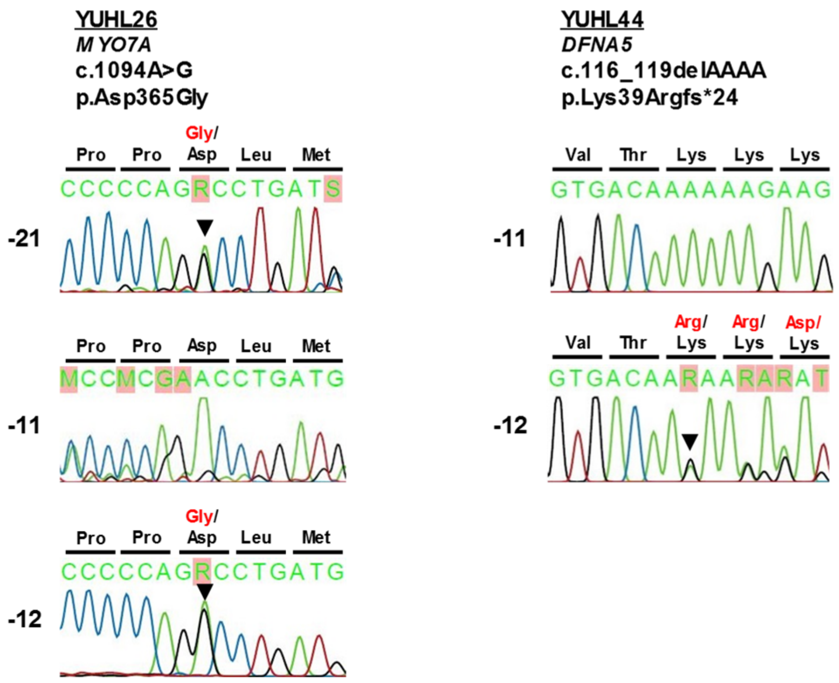

Figure S4. **Sequencing traces of mutations detected in *MYO7A* and *DFNA5*.**

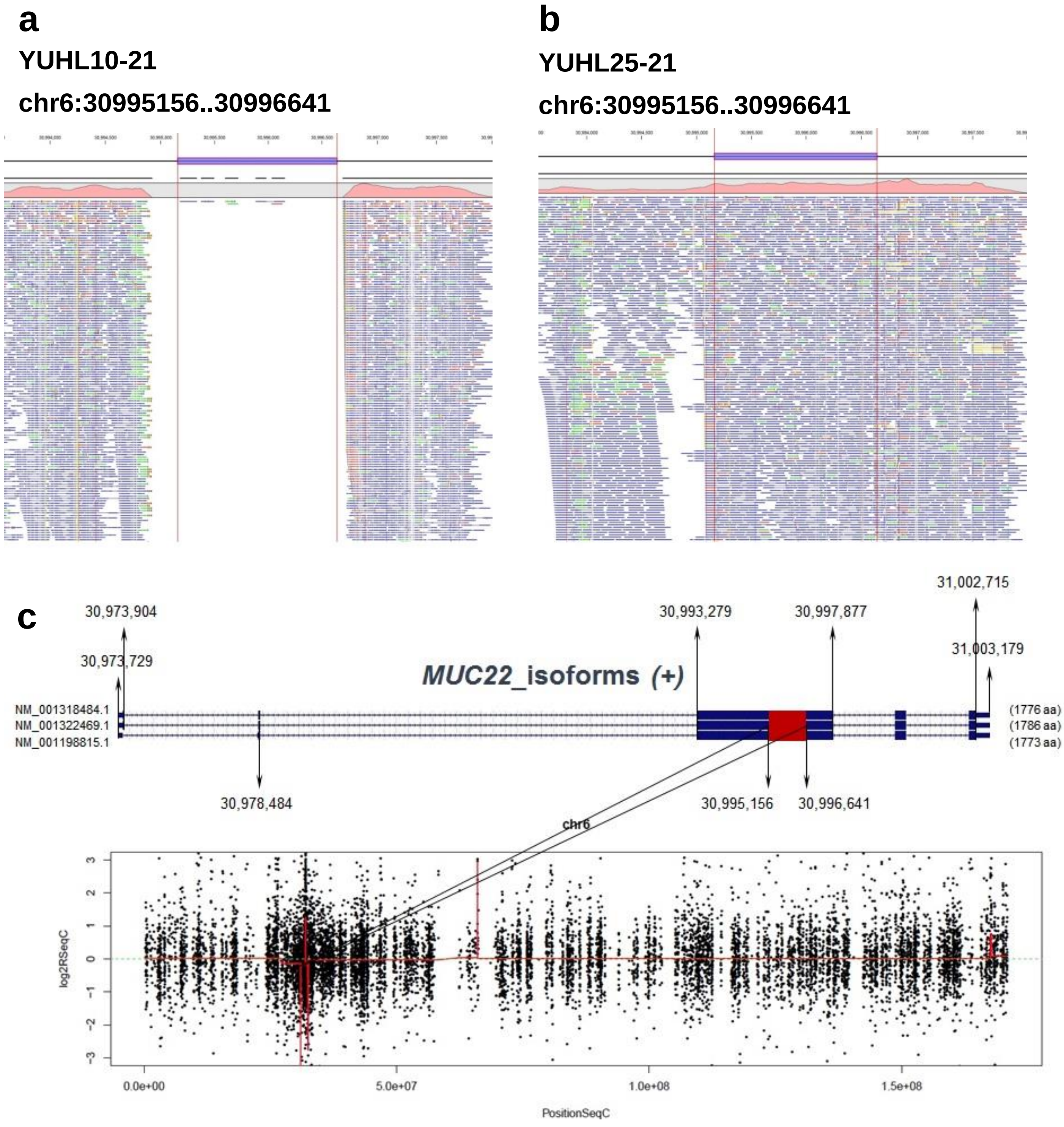

Figure S5. Copy number variation.

Table S1. Primer sequences for Sanger's sequencing of *SLC26A4* and *GJB2*

SLC26A4	Ex1-1	sense	5′-CCCCTGCGTGGCAGCC-3′
		anti-sense	5′-GTCTTTCCCAACCGATCCTATC-3′
	Ex1-2	sense	5′-TGCAGGAGGGGTAGGATTTCTTT-3′
		anti-sense	5′-TGTGGGCATCTTCAGGGCGAA-3′
	Ex2-1	sense	5′-CTCCCTCCTCGCTGTCCTC-3′
		anti-sense	5′-GCCGCGACACCATGTAGCT-3′
	Ex2-2	sense	5′-GCCGCAGCTCCCCGAGTA-3′
		anti-sense	5′-GCTCCGCTTCTCTCTACGC-3′
	Ex3	sense	5′-TTGGTTGTGACTGAGATTGGATTG-3′
		anti-sense	5′-GGAGACCAGAACTCTCAATCTG-3′
	Ex4-1	sense	5′-TGAATGTAATCACTTTGCATGTGC-3′
		anti-sense	5′-AACATGAGCAAATATAATTGTAAAATATAC-3′
	Ex4-2	sense	5′-TATCCTGACATACTTTATCTTTGGAA-3′
		anti-sense	5′-AACATGAGCAAATATAATTGTAAAATATAC-3′
	Ex5-1	sense	5′-TGATTAATAACTGATTAATTGTTAGAGAC-3′
		anti-sense	5′-TATCATAGTAGTATTTAATACAGTTCCATT-3′
	Ex5-2	sense	5′-CGAACACTTTCTCGTATCCAGCA-3′
		anti-sense	5′-TACATCCATCTATATTTTACTTGTAAGTTC-3′
	Ex6	sense	5′-AAACATTTAATTTTTCTTTCCTTTTCCTTATC-3′
		anti-sense	5′-CCCTGGAGCAAGAAGCAACAC-3′
	Ex7	sense	5′-AGCAGCAGGAAGTATATAAAATTATTTTC-3′
		anti-sense	5′-TGCTGAACTTTCTAACTACATATTCTAC-3′
	Ex8	sense	5′-ATTATTAAAACCAATGGAGTTTTTAACATC-3′
		anti-sense	5′-TATTAGTACTAAGAGGAACACCAC-3′
	Ex9-1	sense	5′-GTCAAATCTTCACAGCATTTTTCAC-3′
		anti-sense	5′-TGCAGCCAGCATCTCCGAGAA-3′
	Ex9-2	sense	5′-CTGAACTTCCACCTGTGAGCT-3′
		anti-sense	5′-TTCCAGCCCTATAAAACCAGTTC-3′
	Ex10	sense	5′-ATCGTTGTCATCCAGTCTCTTCC-3′
		anti-sense	5′-ATGGATATCATAAGGCTGTTGTTCC-3′

	Ex11	sense	5′-GAGAAGGACGAATCCTTTTCATAG-3′
		anti-sense	5′-AATCGGTATGCAGAGAAGCAGG-3′
	Ex12	sense	5′-TTCCCTGAATAACACAGCCTTCTC-3′
		anti-sense	5′-GAAGCATATAAGAACCAAATCTTATTTC-3′
	Ex13	sense	5′-ACATTAATATAATTCTTTTCATTTCTATTTTTTTCC-3′
		anti-sense	5′-AGTGTGTTGTTCTGTTTATAGGAAATC-3′
	Ex14	sense	5′-CGGCTGTTCCAAAAAATCTTGAC-3′
		anti-sense	5′-CCAAGTCCAGCAAATGTCTCAC-3′
	Ex15	sense	5′-TTGAAATTATTTAATCCCAGACAATTTC-3′
		anti-sense	5′-TCTCAAAAGAGGTTAGAAAACAAATTTC-3′
	Ex16	sense	5′-CATTTTAAGTAACTTGACATTTATTTCC-3′
		anti-sense	5′-TAAGGGGGAAAAGAAAGATGTCATC-3′
	Ex17-1	sense	5′-AAATCTTTGACAATTAAGTTGACAGTG-3′
		anti-sense	5′-GGAACGTTCACTTTGACTGGAAG-3′
	Ex17-2	sense	5′-ATAGAGATTCAAGTGGATTGGAACTC-3′
		anti-sense	5′-CAAATGTATAATTCAGAAAACCAGAACC-3′
	Ex18	sense	5′-AGGTTGTTAATTGTTACAAACTCTCC-3′
		anti-sense	5′-AAGGGCTTACGGGAAAGTCTTAC-3′
	Ex19-1	sense	5′-ACTAACAAAACATTGTGTCTTTCTTTTG-3′
		anti-sense	5′-TAGAAAAGATACATCTGTAGAAAGGTTG-3′
	Ex19-2	sense	5′-ACTAACAAAACATTGTGTCTTTCTTTTG-3′
		anti-sense	5′-CCGTCAAAAAGAATGTGTCCTTTCT-3′
	Ex20	sense	5′-TGCTATTCTATTTCTACCCTGTGTTC-3′
		anti-sense	5′-ACTCTAATTATTCAAAATGTAATTCAAATATTTTC-3′
	Ex21	sense	5′-TTCAGTTGTATCAACACTTTGTTTTCC-3′
		anti-sense	5′-CATTGAGGAAGTTTTGTCTTGTATTC-3′
GJB2	Ex2-1	sense	5'-TGGTGTTTGCTCAGGAAGAG-3'
		anti-sense	5'-TTGTGTAGGTCCACCACAGG-3'
	Ex2-2	sense	5'-GCCTACCGGAGACATGAGAA-3'
		anti-sense	5'-GGCCTACAGGGGTTTCAAAT-3'

Table S2. Filtering process for variants from normal reference sequence (VRS) following whole exome sequencing (WES) in YUHL.

Individual	Total sequence reads	Matched Reads (percentage of reads)	Total number of variants detected	[a]dbSNP138 (MAF<1%)	[b]Variants filtered by 32 control	[c]Nonsynoy-mous and splice variants	% c / a	Located within splice site	Deletion or Insertion	Stop codon gained or lost	Missense
YUHL8-21	56,886,008	56,772,683 (99.80%)	179,970	49,441	16,540	1,035	2.09%	76	46	12	901
YUHL10-21	58,952,558	58,843,748 (99.82%)	175,270	46,130	15,338	996	2.16%	66	31	11	888
YUHL13-21	88,416,680	88,248,060 (99.81%)	206,699	53,182	17,689	762	1.43%	44	38	15	665
YUHL14-21	66,659,216	66,531,054 (99.81%)	194,398	52,428	17,883	909	1.73%	55	37	9	808
YUHL20-21	71,029,998	70,889,041 (99.80%)	190,036	51,104	17,041	891	1.74%	70	37	8	776
YUHL21-21	78,198,704	77,247,208 (98.78%)	194,277	53,546	22,538	874	1.63%	38	55	17	764
YUHL24-21	85,635,052	85,401,914 (99.73%)	191,531	38,741	9,997	565	1.46%	38	30	8	489
YUHL25-21	108,081,258	107,763,230 (99.71%)	195,767	39,273	15,217	898	2.29%	54	77	16	751
YUHL26-21	96,145,100	95,832,419 (99.67%)	200,817	41,451	16,802	912	2.20%	74	64	5	769
YUHL36-21	96,689,608	94,539,588 (97.78%)	194,569	39,346	15,327	910	2.31%	61	73	17	759
YUHL40-21	58,508,176	58,080,725 (99.27%)	163,533	34,035	12,587	910	2.67%	67	65	13	765
YUHL43-21	50,266,852	49,975,337 (99.42%)	164,309	34,646	16,010	1,040	3.00%	95	92	11	842
YUHL44-21	51,690,290	51,376,008 (99.39%)	170,215	35,587	13,677	929	2.61%	82	63	17	767
Mean value / [d]SD	74,396,885 / 19,167,152	73,961,617 (99.41%)	186,261 / 14,076	43,762 / 7,450	15,896 / 2,967	895 / 122	2.04%	63 / 17	54 / 20	12 / 4	765 / 103

[a]Variants which are not common dbSNP138 (MAF<1%)
[b]Variants which are not shared by 32 control of healthy individuals.
[c]Variants which are located in nonsynonymous and splice site
[d]Standard deviation

Table S3. 72 known NSHL genes examined in this study.

#	Gene symbol	Gene Name	Accession #	MIM-phenotype #	Mode	Coding Exons
1	*ACTG1*	actin gamma 1	NM_001199954	102560	AD	6
2	*ADCY1*	adenylate cyclase 1	NM_021116	103072	AR	20
3	*BSND*	barttin CLCNK type accessory beta subunit	NM_057176	606412	AR	4
4	*CCDC50*	coiled-coil domain containing 50	NM_178335	611051	AD	12
5	*CDH23*	cadherin related 23	NM_022124	605516	AR	68
6	*CEACAM16*	carcinoembryonic antigen related cell adhesion molecule 16	NM_001039213	614591	AD	7
7	*CIB2*	calcium and integrin binding family member 2	NM_006383	605564	AR	6
8	*CLDN14*	claudin 14	NM_144492	605608	AR	3
9	*COCH*	cochlin	NM_004086	603196	AD	12
10	*COL11A2*	collagen type XI alpha 2 chain	NM_080680	120290	AD	66
11	*DFNA5*	DFNA5, deafness associated tumor suppressor	NM_004403	608798	AD	10
12	*DFNB59*	deafness, autosomal recessive 59	NM_001042702	610219	AR	7
13	*DIABLO*	diablo IAP-binding mitochondrial protein	NM_019887	605219	AD	7
14	*DIAPH1*	diaphanous related formin 1	NM_001314007	602121	AD	29
15	*ELMOD3*	ELMO domain containing 3	NM_001135021	615427	AR	15
16	*EPS8*	epidermal growth factor receptor pathway substrate 8	NM_004447	600206	AR	21
17	*ESPN*	espin	NM_031475	606351	AR	13
18	*ESRRB*	estrogen related receptor beta	NM_004452	602167	AR	11
19	*EYA4*	EYA transcriptional coactivator and phosphatase 4	NM_004100	603550	AD	20
20	*FAM65B*	family with sequence similarity 65 member B	NM_014722	611410	AR	23

21	*GIPC3*	GIPC PDZ domain containing family member 3	NM_133261	608792	AR	6
22	*GJB2*	gap junction protein beta 2	NM_004004	121011	AD	2
23	*GPSM2*	G-protein signaling modulator 2	NM_001321039	609245	AR	16
24	*GRHL2*	grainyhead like transcription factor 2	NM_024915	608576	AD	16
25	*GRXCR1*	glutaredoxin and cysteine rich domain containing 1	NM_001080476	613283	AR	4
26	*GRXCR2*	glutaredoxin and cysteine rich domain containing 2	NM_001080516	615762	AR	3
27	*HGF*	hepatocyte growth factor	NM_000601	142409	AR	18
28	*HOMER2*	homer scaffolding protein 2	NM_199330	604799	AD	9
29	*ILDR1*	immunoglobulin like domain containing receptor 1	NM_001199799	609739	AR	8
30	*KARS*	lysyl-tRNA synthetase	NM_001130089	601421	AR	15
31	*KCNQ4*	potassium voltage-gated channel subfamily Q member 4	NM_004700	603537	AD	14
32	*LHFPL5*	lipoma HMGIC fusion partner-like 5	NM_182548	609427	AR	4
33	*LOXHD1*	lipoxygenase homology domains 1	NM_144612	613072	AR	40
34	*LRTOMT*	leucine rich transmembrane and O-methyltransferase domain containing	NM_001145309	612414	AR	9
35	*MARVELD2*	MARVEL domain containing 2	NM_001038603	610572	AR	7
36	*MET*	MET proto-oncogene, receptor tyrosine kinase	NM_001127500	164860	AR	21
37	*MIR96*	microRNA 96	NR_029512	611606	AD	1
38	*MSRB3*	methionine sulfoxide reductase B3	NM_001193460	613719	AR	8
39	*MYH14*	myosin heavy chain 14	NM_001145809	608568	AD	43
40	*MYH9*	myosin heavy chain 9	NM_002473	160775	AD	41
41	*MYO15A*	myosin XVA	NM_016239	602666	AR	65
42	*MYO3A*	myosin IIIA	NM_017433	606808	AR	35

43	*MYO6*	myosin VI	NM_004999	600970	AD	35
44	*MYO7A*	myosin VIIA	NM_000260	276903	AD	49
45	*OSBPL2*	oxysterol binding protein like 2	NM_144498	606731	AD	14
46	*OTOA*	otoancorin	NM_144672	607038	AR	28
47	*OTOF*	otoferlin	NM_194248	603681	AR	47
48	*OTOG*	otogelin	NM_001292063	604487	AR	56
49	*OTOGL*	otogelin like	NM_173591	614925	AR	58
50	*P2RX2*	purinergic receptor P2X 2	NM_174873	600844	AD	12
51	*PCDH15*	protocadherin related 15	NM_001142769	605514	AR	36
52	*POU3F4*	POU class 3 homeobox 4	NM_000307	300039	XR	1
53	*POU4F3*	POU class 4 homeobox 3	NM_002700	602460	AD	2
54	*PRPS1*	phosphoribosyl pyrophosphate synthetase 1	NM_002764	311850	XR	7
55	*RDX*	radixin	NM_001260492	179410	AR	16
56	*S1PR2*	sphingosine-1-phosphate receptor 2	NM_004230	605111	AR	2
57	*SERPINB6*	serpin family B member 6	NM_001195291	173321	AR	8
58	*SLC26A4*	solute carrier family 26 member 4	NM_000441	605646	AR	21
59	*SMPX*	small muscle protein, X-linked	NR_045617	300226	XR	6
60	*STRC*	stereocilin	NM_153700	606440	AR	29
61	*SYNE4*	spectrin repeat containing nuclear envelope family member 4	NM_001039876	615535	AR	8
62	*TBC1D24*	TBC1 domain family member 24	NM_001199107	613577	AR	8
63	*TECTA*	tectorin alpha	NM_005422	602574	AD	23
64	*TMC1*	transmembrane channel like 1	NM_138691	606706	AD	24
65	*TMIE*	transmembrane inner ear	NM_147196	607237	AR	4

66	*TMPRSS3*	transmembrane protease, serine 3	NM_024022	605511	AR	13
67	*TPRN*	taperin	NM_001128228	613354	AR	4
68	*TRIOBP*	TRIO and F-actin binding protein	NM_001039141	609761	AR	24
69	*TSPEAR*	thrombospondin type laminin G domain and EAR repeats	NM_001272037	612920	AR	13
70	*USH1C*	USH1 protein network component harmonin	NM_153676	605242	AR	27
71	*WFS1*	wolframin ER transmembrane glycoprotein	NM_001145853	606201	AD	8
72	*WHRN*	whirlin	NM_001083885	607928	AR	12

Table S4. Mean coverage of target regions in 72 known NSHL genes

Chr	Start	End	Name	YUHL 8-21	YUHL 10-21	YUHL 13-21	YUHL 14-21	YUHL 20-21	YUHL 21-21	YUHL 24-21	YUHL 25-21	YUHL 26-21	YUHL 36-21	YUHL 40-21	YUHL 43-21	YUHL 44-21	Average (highlight<=1)
chr1	6485017	6485309	ESPN	4.32	8.37	1.77	8.22	12.97	4.60	9.74	7.37	5.18	15.29	5.16	2.09	7.90	7.09
chr1	6488287	6488479	ESPN	53.11	64.49	53.84	61.27	91.08	47.43	72.31	81.97	71.88	136.20	63.95	29.79	65.74	68.94
chr1	6500315	6500500	ESPN	22.47	19.46	15.26	22.92	39.44	16.26	36.15	32.63	22.42	47.45	13.95	13.61	24.87	25.17
chr1	6500687	6500868	ESPN	9.91	9.49	4.53	6.04	20.30	3.18	8.57	10.48	6.48	14.45	10.35	5.36	7.42	9.10
chr1	6500995	6501125	ESPN	21.04	35.37	25.54	25.40	43.39	23.56	34.50	47.74	37.76	83.85	28.19	16.85	32.99	35.27
chr1	6504542	6504742	ESPN	53.03	64.86	48.44	57.90	110.37	48.33	70.69	93.09	77.31	171.47	78.70	45.57	72.42	76.65
chr1	6505725	6505995	ESPN	14.68	16.43	12.06	16.72	30.31	13.67	17.92	25.66	18.12	43.40	17.62	12.70	16.31	19.94
chr1	6508702	6509151	ESPN	6.24	9.54	5.74	7.26	14.90	8.29	8.09	9.10	8.10	17.04	6.51	7.11	7.79	8.99
chr1	6511664	6511808	ESPN	38.51	58.52	32.48	56.30	78.68	40.51	54.37	82.44	77.10	108.56	51.81	39.66	60.95	59.91
chr1	6511894	6512156	ESPN	19.97	28.75	17.22	25.63	34.45	22.19	27.82	42.08	39.09	62.75	31.41	15.57	27.13	30.58
chr1	6517245	6517323	ESPN	41.35	36.62	36.81	42.63	70.24	26.11	34.97	51.90	43.90	83.86	54.58	22.97	45.85	45.50
chr1	6517422	6517432	ESPN	65.00	34.55	50.18	60.73	90.36	32.82	51.73	68.09	59.73	99.00	75.36	26.82	66.82	59.53
chr1	6520060	6520206	ESPN	21.48	27.75	14.61	22.86	47.64	21.67	27.82	35.46	31.37	66.41	28.35	15.61	29.19	30.09
chr1	41249767	41250079	KCNQ4	54.51	88.40	55.94	47.58	79.25	57.72	82.20	93.75	87.29	93.98	72.33	49.06	62.84	71.84
chr1	41282938	41283027	KCNQ4	32.91	49.71	28.29	31.47	59.63	27.77	42.36	81.54	42.43	62.56	43.01	31.69	37.82	44.45
chr1	41283837	41283962	KCNQ4	58.47	89.85	65.71	86.00	121.60	66.21	98.89	130.98	106.97	186.26	78.10	48.43	113.16	94.79
chr1	41284178	41284352	KCNQ4	41.83	41.38	36.83	54.06	82.97	29.50	47.20	78.50	55.55	104.69	48.13	31.67	56.70	54.36
chr1	41285020	41285144	KCNQ4	69.63	76.65	55.10	117.61	145.62	69.78	80.76	121.08	88.90	218.22	104.55	53.00	106.25	100.08
chr1	41285548	41285657	KCNQ4	44.76	71.67	44.89	59.15	81.86	49.58	52.85	84.05	71.65	117.52	57.86	39.94	53.18	64.65
chr1	41285838	41285932	KCNQ4	69.72	87.15	52.39	78.02	118.19	64.05	83.47	138.03	99.77	180.43	81.36	54.67	88.14	92.27
chr1	41287987	41288074	KCNQ4	14.69	21.49	10.16	15.78	30.64	14.44	22.42	35.25	23.35	48.98	17.32	12.56	24.43	22.26
chr1	41289770	41289930	KCNQ4	22.19	40.43	29.80	19.89	43.68	31.70	42.45	44.76	39.96	57.48	23.65	26.91	33.01	35.24
chr1	41296757	41296976	KCNQ4	84.63	92.92	73.66	91.49	138.45	72.24	91.63	130.07	90.92	232.00	84.60	63.18	111.77	103.82
chr1	41298687	41298785	KCNQ4	115.93	107.31	62.54	112.23	191.09	93.36	133.40	169.25	128.10	244.04	109.85	74.75	136.14	128.49
chr1	41300640	41300770	KCNQ4	64.18	46.59	33.56	72.15	104.79	40.37	57.95	76.87	57.57	144.08	77.19	34.50	79.46	67.48
chr1	41303338	41303466	KCNQ4	36.08	60.40	31.68	38.02	56.98	35.36	57.48	61.55	65.56	113.04	49.51	29.24	49.86	52.91
chr1	41303984	41304195	KCNQ4	99.68	115.32	81.13	123.14	189.49	95.54	102.07	178.74	122.83	295.86	110.83	78.82	128.02	132.79
chr1	55464861	55465036	BSND	45.79	37.15	28.07	39.76	67.09	40.20	51.34	81.22	48.14	100.59	33.56	29.77	47.51	50.22
chr1	55470696	55470789	BSND	73.54	77.04	102.35	78.01	88.48	165.74	121.50	151.51	118.04	111.04	78.01	71.41	76.65	103.06
chr1	55472671	55472945	BSND	37.13	40.87	37.24	47.13	52.21	24.14	44.20	47.80	48.93	97.49	41.49	34.73	39.76	46.11
chr1	55473888	55474301	BSND	95.18	120.58	96.75	121.09	173.49	96.25	127.33	164.75	140.45	255.33	117.21	82.74	120.90	132.60
chr1	109427898	109428200	GPSM2	13.25	25.29	22.89	11.03	19.73	14.83	26.64	27.94	31.47	0.33	19.20	21.73	13.90	19.53
chr1	109439487	109439707	GPSM2	39.86	57.24	43.52	38.66	59.68	36.22	57.07	94.51	60.17	20.25	40.37	38.25	47.30	48.82
chr1	109440115	109440249	GPSM2	83.90	142.94	88.55	72.36	99.04	81.87	141.57	182.66	151.18	47.14	101.15	72.57	73.06	105.41
chr1	109440582	109440723	GPSM2	23.73	28.92	20.86	19.44	38.27	23.33	28.28	56.09	40.35	15.08	34.43	15.78	29.58	28.71
chr1	109441265	109441387	GPSM2	73.70	94.22	57.82	50.41	84.40	60.66	98.44	159.54	112.54	36.00	75.80	48.13	68.58	79.30
chr1	109441502	109441616	GPSM2	25.75	45.35	25.46	20.63	35.70	23.16	44.56	68.18	55.33	1.30	19.18	27.70	24.17	32.69
chr1	109444413	109444567	GPSM2	72.06	113.05	71.83	55.69	105.33	65.51	116.08	144.05	123.55	2.24	84.26	66.81	67.94	85.04
chr1	109445749	109445856	GPSM2	50.74	80.06	45.40	27.53	56.97	36.27	64.32	94.09	81.81	8.49	49.44	44.29	55.02	53.28
chr1	109446748	109446876	GPSM2	11.62	27.97	13.98	10.17	22.83	14.59	27.25	42.31	33.60	0.00	13.97	16.81	13.27	19.59
chr1	109456961	109457030	GPSM2	23.67	53.64	31.23	19.01	20.40	27.94	49.66	54.39	52.84	9.71	31.97	36.17	22.91	34.22
chr1	109461236	109461411	GPSM2	77.29	136.08	73.08	77.32	95.19	87.15	134.83	182.21	151.27	19.65	86.71	73.61	75.30	99.53
chr1	109465040	109465198	GPSM2	74.00	151.08	97.71	72.55	119.45	77.07	156.04	213.44	151.68	2.82	100.14	95.91	87.84	109.32
chr1	109466623	109466836	GPSM2	113.86	152.74	109.36	99.91	148.16	90.99	161.04	218.47	190.28	115.04	130.00	100.88	122.48	135.89
chr1	109472324	109472597	GPSM2	60.70	103.64	67.56	60.58	94.06	52.11	91.53	130.44	121.32	18.98	70.54	61.80	67.28	77.77
chr2	26680909	26681088	OTOF	110.01	137.12	66.88	121.77	177.34	111.97	128.93	151.02	138.58	281.93	129.42	87.21	148.09	136.85
chr2	26682877	26683073	OTOF	35.18	24.03	26.23	43.94	48.39	20.61	34.11	59.74	29.85	70.26	38.45	17.88	42.59	37.39
chr2	26683516	26683615	OTOF	57.85	68.47	49.11	68.68	100.45	47.20	68.45	87.04	78.86	117.48	59.59	40.60	64.16	70.32
chr2	26683721	26683898	OTOF	110.89	135.52	98.13	127.43	212.56	103.40	140.31	209.85	151.76	277.80	119.96	87.93	148.50	147.96
chr2	26684565	26684805	OTOF	74.99	76.64	58.90	70.53	132.67	74.22	86.05	129.81	96.68	190.63	83.18	63.30	90.93	94.80
chr2	26684952	26685049	OTOF	65.05	49.01	44.36	59.10	91.85	55.08	60.30	109.02	67.73	131.09	53.68	39.66	76.18	68.83
chr2	26686352	26686439	OTOF	47.65	49.57	33.15	54.75	84.61	38.53	61.97	84.28	49.88	72.45	39.99	32.58	38.48	54.12
chr2	26686833	26686974	OTOF	53.69	75.89	51.61	72.11	95.57	52.36	61.65	89.85	66.82	169.32	84.31	52.83	65.73	77.17
chr2	26687738	26687897	OTOF	37.79	50.69	29.22	46.93	69.80	41.29	47.23	50.23	53.13	110.22	39.32	31.28	41.99	50.59
chr2	26688541	26688710	OTOF	63.54	78.12	64.28	84.46	113.89	76.68	85.88	119.50	103.00	174.82	62.34	58.36	76.65	90.41
chr2	26688818	26688944	OTOF	60.87	92.29	64.58	70.57	108.78	52.72	97.75	143.86	92.08	164.76	65.53	63.07	78.60	89.74
chr2	26689583	26689719	OTOF	73.99	125.43	74.09	76.61	131.09	79.49	111.80	161.66	125.63	207.58	113.15	74.67	102.47	112.93
chr2	26689968	26690101	OTOF	91.04	103.92	74.96	83.34	158.15	97.95	136.01	168.11	121.63	234.78	85.62	74.48	113.68	119.17
chr2	26690234	26690369	OTOF	34.44	39.10	27.37	25.44	50.06	44.94	40.59	59.15	54.81	72.66	44.19	36.84	35.51	44.13
chr2	26691277	26691342	OTOF	18.41	42.92	30.26	24.89	41.41	22.80	36.77	47.36	46.77	46.61	38.09	23.85	30.08	35.01
chr2	26693462	26693589	OTOF	33.80	36.27	20.23	30.34	45.58	29.48	31.26	45.23	38.64	56.40	34.73	16.60	33.21	34.88
chr2	26693990	26694018	OTOF	54.24	60.55	59.10	67.21	104.86	44.10	69.90	68.31	78.38	154.28	68.24	46.90	93.21	73.01
chr2	26695388	26695517	OTOF	59.54	62.78	41.43	70.00	109.88	47.23	73.15	103.79	72.42	158.98	63.92	42.15	67.09	75.44
chr2	26696001	26696162	OTOF	47.79	49.01	36.44	64.52	80.38	30.54	55.76	61.95	59.96	117.14	47.55	36.02	51.93	57.26
chr2	26696275	26696435	OTOF	47.55	73.28	39.51	71.86	96.76	47.11	73.87	89.83	68.58	144.04	72.70	45.23	74.57	72.53
chr2	26696860	26696978	OTOF	35.88	49.34	42.77	48.29	71.11	30.76	46.85	79.36	58.53	87.32	39.97	30.64	45.55	51.73
chr2	26697382	26697542	OTOF	80.02	80.82	55.91	75.10	124.30	63.10	76.17	105.25	82.81	206.00	75.53	62.76	69.81	90.65
chr2	26698228	26698361	OTOF	36.94	51.26	24.74	35.77	61.09	30.52	52.85	81.78	55.28	93.40	58.32	23.65	58.70	50.47
chr2	26698783	26698906	OTOF	74.14	56.94	55.38	85.48	116.90	66.80	72.54	98.23	91.38	217.06	78.49	52.12	91.19	88.79
chr2	26698997	26699185	OTOF	26.27	31.63	20.17	27.17	73.48	33.61	35.04	53.13	42.66	76.98	31.99	23.94	43.03	39.67
chr2	26699760	26699911	OTOF	39.14	37.01	24.05	42.57	68.53	31.83	48.17	57.27	51.95	106.76	59.16	36.34	48.57	50.23
chr2	26700041	26700156	OTOF	32.76	39.48	28.97	38.34	68.54	28.45	42.24	52.56	48.61	84.36	30.20	29.63	37.79	43.68
chr2	26700285	26700374	OTOF	28.99	24.93	17.96	29.81	55.41	21.70	24.97	44.37	35.70	66.96	32.92	19.74	45.52	33.62
chr2	26700518	26700761	OTOF	48.41	78.59	48.11	67.36	97.75	55.76	94.24	129.83	99.82	126.55	66.73	65.63	66.98	81.57
chr2	26702133	26702252	OTOF	30.37	49.75	26.06	38.98	71.89	37.66	47.98	56.44	51.98	102.71	29.91	23.17	47.43	47.24
chr2	26702342	26702521	OTOF	45.90	65.87	33.05	45.72	65.61	40.57	57.24	74.17	69.83	107.23	41.87	32.74	55.07	56.65

chr2	26703072	26703179	OTOF	24.91	46.15	24.32	29.39	63.54	28.94	35.69	70.97	48.33	86.10	45.54	21.51	39.13	43.78
chr2	26703655	26703877	OTOF	46.10	53.43	35.28	57.29	113.91	48.04	68.26	87.23	78.63	146.52	44.54	40.89	67.43	68.34
chr2	26705275	26705460	OTOF	32.79	38.87	33.97	37.77	57.37	37.52	43.64	71.18	50.28	112.99	43.02	27.11	49.89	48.88
chr2	26706331	26706516	OTOF	29.22	23.44	14.45	19.32	38.08	19.41	24.82	35.09	21.41	46.95	24.83	17.90	24.51	26.24
chr2	26707343	26707501	OTOF	62.67	60.52	38.87	55.84	114.83	55.75	68.71	95.25	69.09	154.48	72.13	44.98	52.55	74.43
chr2	26712080	26712163	OTOF	48.88	47.79	33.11	57.79	93.35	42.43	57.32	86.67	58.26	149.88	48.50	28.21	63.39	62.68
chr2	26712547	26712608	OTOF	80.21	118.02	66.44	112.11	167.26	67.94	97.42	174.61	127.16	205.40	88.61	55.18	89.60	113.36
chr2	26717811	26717941	OTOF	59.42	69.41	41.27	73.76	102.60	54.59	75.94	100.21	81.05	145.96	89.08	55.64	72.21	79.08
chr2	26724623	26724676	OTOF	32.09	55.76	40.19	40.93	72.57	26.11	59.85	70.31	81.07	54.65	44.91	33.89	52.78	51.03
chr2	26725169	26725294	OTOF	27.67	39.25	23.57	25.06	44.40	19.00	36.98	47.62	28.12	53.63	30.49	23.30	37.84	33.26
chr2	26726641	26726713	OTOF	59.07	83.21	51.34	78.32	103.11	49.38	85.73	112.49	91.12	125.56	75.52	54.64	63.42	80.79
chr2	26739287	26739467	OTOF	39.72	47.91	34.97	44.12	72.42	38.89	53.94	65.48	59.72	113.20	60.38	31.75	51.14	55.21
chr2	26741879	26741977	OTOF	64.58	72.98	50.41	84.06	106.14	60.96	66.32	109.06	81.69	169.17	74.76	47.92	71.38	82.34
chr2	26750701	26750788	OTOF	49.84	62.56	51.11	48.48	96.40	54.26	59.80	104.28	79.61	108.45	49.91	44.52	65.57	67.44
chr2	26760585	26760642	OTOF	37.78	38.69	31.40	24.09	58.17	26.93	28.64	65.02	32.03	66.81	40.53	34.43	34.07	40.38
chr2	26781362	26781439	OTOF	46.22	55.79	39.58	58.77	115.01	63.04	68.77	92.50	74.22	157.97	75.15	44.24	64.79	74.27
chr2	85582200	85582293	ELMOD3	0.00	0.07	0.82	0.00	0.00	4.40	0.00	0.50	1.00	0.00	0.00	0.00	0.00	0.57
chr2	85582679	85582839	ELMOD3	0.45	0.78	0.00	0.00	1.63	0.63	0.46	0.00	0.53	0.69	0.00	0.00	0.00	0.43
chr2	85583726	85583790	ELMOD3	0.32	0.63	0.88	0.00	0.71	1.94	0.00	1.00	0.00	0.18	0.78	0.66	0.00	0.59
chr2	85584091	85584375	ELMOD3	50.50	78.88	50.30	48.39	71.99	39.68	74.22	104.23	88.19	24.76	62.27	49.69	60.84	61.92
chr2	85589310	85589383	ELMOD3	64.74	141.18	86.66	62.36	101.01	70.27	116.24	165.53	144.41	32.49	82.32	70.04	69.55	94.77
chr2	85590221	85590289	ELMOD3	13.75	17.75	16.78	21.68	27.62	17.13	23.74	49.49	32.43	33.58	22.09	24.67	12.01	25.06
chr2	85595810	85595877	ELMOD3	20.68	35.63	24.93	18.71	30.19	29.65	33.26	45.10	29.32	41.88	35.07	25.10	18.35	30.79
chr2	85596863	85596953	ELMOD3	39.30	39.66	27.90	38.75	58.33	21.52	54.07	59.60	52.11	90.93	53.13	31.60	42.84	47.24
chr2	85598210	85598332	ELMOD3	57.02	63.14	55.34	59.26	106.52	46.74	64.22	97.69	81.96	145.43	76.52	39.10	72.79	74.41
chr2	85598564	85598685	ELMOD3	36.39	38.95	37.04	42.48	56.31	35.81	42.52	59.78	53.07	87.36	58.23	27.14	42.84	47.92
chr2	85604468	85604597	ELMOD3	66.62	62.13	45.42	67.69	88.62	51.48	73.06	99.34	58.43	171.28	72.08	45.19	66.89	75.11
chr2	85616875	85616950	ELMOD3	62.67	73.64	59.01	71.01	89.34	61.62	96.16	126.36	91.51	164.46	84.30	54.53	82.41	86.22
chr2	85617262	85617621	ELMOD3	85.01	104.72	83.17	93.24	121.44	81.56	119.29	175.77	119.54	167.15	106.13	75.51	97.27	111.04
chr2	85617884	85618085	ELMOD3	44.41	54.78	89.33	53.23	53.23	119.08	50.73	78.49	58.91	54.04	39.20	28.36	34.27	60.32
chr2	179318116	179318347	DFNB59	58.27	109.11	78.03	53.19	68.25	55.81	93.56	128.72	108.33	17.15	80.81	55.92	72.44	75.60
chr2	179319060	179319254	DFNB59	12.11	39.81	25.92	16.33	21.41	25.38	35.86	69.78	44.86	0.52	29.08	20.98	25.96	28.50
chr2	179320738	179320878	DFNB59	54.65	82.65	57.90	56.07	95.79	45.55	87.98	123.63	95.27	54.84	64.82	52.60	55.72	72.64
chr2	179323238	179323354	DFNB59	77.74	124.09	98.20	79.50	102.97	82.23	141.38	171.14	144.07	7.62	90.71	82.91	86.74	100.21
chr2	179325076	179325173	DFNB59	29.78	67.53	36.67	43.82	51.76	45.04	69.77	88.84	74.96	4.43	33.45	28.61	38.70	47.89
chr2	179325710	179326001	DFNB59	120.19	190.67	113.50	97.11	136.12	103.29	178.67	284.02	228.10	24.02	127.10	111.60	100.29	142.87
chr3	46742979	46743070	TMIE	30.79	43.14	20.76	34.11	58.20	38.79	45.67	49.32	48.24	68.60	36.71	39.72	40.68	42.84
chr3	46747281	46747397	TMIE	53.18	58.30	48.50	63.12	76.32	52.26	61.71	90.76	77.47	133.20	50.06	46.16	60.60	67.59
chr3	46750617	46750765	TMIE	57.15	57.56	43.72	66.01	108.56	48.38	60.97	81.72	84.70	144.58	77.50	46.09	75.26	73.08
chr3	46751070	46751178	TMIE	36.66	21.85	21.89	34.58	59.75	19.12	21.61	27.54	31.88	48.97	37.97	21.11	34.89	31.91
chr3	121707215	121707255	ILDR1	54.78	74.00	41.00	51.66	78.63	36.80	76.51	115.51	90.02	48.80	69.07	41.46	52.39	64.86
chr3	121711998	121712817	ILDR1	52.33	66.72	43.45	53.96	95.77	49.49	69.96	90.05	73.58	136.41	57.43	47.77	69.88	69.74
chr3	121713030	121713160	ILDR1	40.27	61.67	44.69	51.38	100.43	49.75	70.95	78.90	75.71	101.90	65.59	41.59	65.96	65.24
chr3	121720156	121720301	ILDR1	21.68	26.99	16.42	34.16	49.20	17.46	25.22	41.81	20.92	41.47	37.21	25.71	21.55	29.85
chr3	121720593	121720711	ILDR1	25.89	33.50	17.56	19.47	43.42	24.19	37.09	41.92	43.85	29.25	25.30	19.18	27.06	30.05
chr3	121724092	121724240	ILDR1	57.54	88.05	55.56	60.50	118.52	71.37	96.56	116.68	105.07	172.66	73.74	81.59	80.82	91.49
chr3	121725839	121726008	ILDR1	29.61	29.61	24.06	21.99	41.58	24.32	34.88	55.28	34.07	56.75	27.30	17.16	30.51	33.05
chr3	121740868	121740924	ILDR1	24.12	24.93	19.07	30.65	57.35	26.33	32.47	44.16	28.60	71.88	39.81	22.46	37.05	35.15
chr3	191047465	191047512	CCDC50	58.69	81.92	37.92	69.25	137.46	53.54	73.44	99.63	77.65	171.73	56.33	43.88	84.48	80.12
chr3	191074878	191074939	CCDC50	40.98	85.19	59.42	51.37	82.34	62.19	76.40	113.73	84.47	33.66	71.61	49.61	54.03	67.58
chr3	191075788	191075913	CCDC50	82.56	148.30	101.23	93.64	128.12	72.14	157.88	230.46	162.02	91.06	107.47	96.83	110.11	122.64
chr3	191078859	191078948	CCDC50	84.91	117.74	75.34	90.90	122.96	66.54	105.42	128.87	126.38	30.18	106.12	78.34	98.67	94.48
chr3	191087709	191087825	CCDC50	76.21	130.74	86.81	79.90	109.79	67.10	138.76	176.80	135.88	12.36	98.96	69.68	94.29	98.58
chr3	191092852	191093378	CCDC50	62.58	76.03	54.46	64.23	93.65	53.51	83.80	96.92	88.49	92.28	67.04	53.15	68.49	73.84
chr3	191097949	191098063	CCDC50	55.39	66.34	51.86	37.97	70.36	49.21	56.62	71.64	93.07	17.68	65.53	48.75	53.87	57.03
chr3	191098477	191098520	CCDC50	33.07	42.00	37.50	29.57	46.45	31.50	33.75	51.14	41.77	6.02	52.25	28.32	32.27	36.11
chr3	191098618	191098721	CCDC50	65.33	81.25	61.63	35.53	80.18	48.23	57.45	73.78	89.22	10.73	83.60	60.85	51.02	62.31
chr3	191100536	191100614	CCDC50	67.66	87.62	57.28	50.56	102.95	48.80	91.70	116.39	97.06	29.53	84.90	54.96	75.65	74.12
chr3	191107286	191107391	CCDC50	85.95	121.19	86.60	77.06	137.67	80.61	138.66	210.26	179.20	42.74	100.12	89.92	112.03	112.50
chr3	191109531	191109549	CCDC50	29.37	47.05	34.68	22.32	39.32	31.79	54.63	56.26	58.42	3.00	28.21	30.58	18.05	36.30
chr4	6279179	6279414	WFS1	39.96	44.56	30.08	36.85	73.73	34.21	37.27	43.84	44.62	92.65	54.31	27.43	52.41	46.63
chr4	6288821	6288902	WFS1	15.37	21.52	21.21	15.74	47.65	26.43	27.72	52.07	42.18	47.68	25.67	17.96	29.06	30.10
chr4	6290715	6290858	WFS1	35.78	48.33	26.24	41.59	65.45	26.81	38.31	67.33	38.56	96.15	48.07	25.80	44.99	46.54
chr4	6292925	6293094	WFS1	64.61	102.65	61.40	95.76	125.01	73.06	116.14	143.51	101.02	194.85	110.82	72.15	101.35	105.08
chr4	6293645	6293724	WFS1	31.73	34.64	20.84	33.94	63.78	30.51	34.46	63.69	42.35	88.09	35.01	19.86	34.66	41.57
chr4	6296769	6296916	WFS1	17.51	29.13	19.16	26.48	45.87	24.44	28.29	50.10	42.47	61.32	26.97	24.04	31.78	32.98
chr4	6302385	6304195	WFS1	99.94	122.16	84.99	114.86	184.91	91.05	130.19	195.02	137.48	272.09	116.13	81.36	130.87	135.85
chr4	42895285	42895667	GRXCR1	87.14	114.80	85.54	85.44	113.55	75.96	133.67	187.37	142.34	111.11	106.68	74.89	102.23	109.87
chr4	42964910	42965151	GRXCR1	117.11	174.57	130.95	109.33	150.92	108.08	179.38	253.52	212.79	129.42	118.93	101.69	129.89	148.89
chr4	43022372	43022436	GRXCR1	20.86	44.95	31.37	11.71	30.46	14.31	34.34	68.14	49.14	1.69	29.97	22.55	26.77	29.96
chr4	43032379	43032557	GRXCR1	39.92	66.89	38.79	39.22	58.16	39.46	69.67	84.25	80.83	51.30	58.44	41.68	55.93	55.72
chr5	68715199	68716358	MARVELD2	76.02	114.41	87.48	85.27	117.05	82.33	127.71	167.63	127.49	126.05	94.88	88.29	88.83	107.88
chr5	68720429	68720463	MARVELD2	36.60	62.83	39.14	28.69	62.14	39.00	61.60	101.77	88.66	6.23	46.89	48.09	30.71	51.80
chr5	68728355	68728502	MARVELD2	59.90	76.09	67.20	69.09	97.61	53.84	95.85	150.03	91.42	55.91	81.76	65.03	73.39	80.31
chr5	68728750	68728920	MARVELD2	50.10	103.30	53.02	57.98	95.37	43.82	103.98	145.79	128.01	27.50	76.02	53.45	53.86	78.20
chr5	68736278	68736327	MARVELD2	18.08	44.16	27.38	24.26	43.64	27.54	42.30	44.42	53.86	0.00	23.38	21.84	20.14	30.91
chr5	68737360	68737481	MARVELD2	18.98	32.75	23.81	12.89	17.83	17.02	32.48	34.10	36.62	0.80	18.30	25.75	19.61	22.61
chr5	140896419	140896575	DIAPH1	31.98	34.45	20.52	29.24	42.20	18.09	40.25	48.90	51.25	49.89	31.71	27.07	34.94	35.46
chr5	140898512	140898563	DIAPH1	31.37	36.44	24.00	36.13	38.35	21.83	40.92	39.83	29.79	11.65	27.54	24.42	37.87	30.19
chr5	140903711	140903796	DIAPH1	29.72	31.12	25.71	47.38	56.44	27.13	37.33	66.35	55.43	58.33	44.90	38.50	44.26	43.19

chr5	140905606	140905740	DIAPH1	44.81	84.16	43.67	50.71	67.13	45.21	87.84	110.53	108.23	71.84	52.30	49.82	58.02	68.02
chr5	140905865	140906028	DIAPH1	79.39	128.77	67.89	83.54	111.61	84.49	129.67	174.11	124.28	83.55	86.43	81.49	86.40	102.94
chr5	140907141	140907264	DIAPH1	51.90	81.51	73.13	57.65	61.97	49.16	106.54	122.53	104.26	18.42	73.02	56.49	53.01	71.38
chr5	140908021	140908149	DIAPH1	38.05	46.99	42.16	35.48	57.97	35.12	59.65	87.60	71.57	49.57	58.05	34.71	52.02	51.41
chr5	140908270	140908508	DIAPH1	78.24	111.11	87.20	78.79	115.29	70.60	120.41	181.94	145.58	79.10	103.40	81.42	91.91	104.42
chr5	140908740	140908840	DIAPH1	54.78	93.90	60.27	63.24	99.75	59.80	100.70	139.51	125.97	71.64	80.28	55.80	71.79	83.80
chr5	140909171	140909264	DIAPH1	72.87	67.33	80.18	71.83	113.31	76.02	81.13	149.97	111.33	70.51	82.38	79.60	83.80	88.04
chr5	140913903	140914000	DIAPH1	86.48	138.30	90.48	76.89	103.27	102.67	165.05	171.57	178.84	10.34	97.08	99.08	83.41	110.00
chr5	140915622	140915629	DIAPH1	24.50	66.88	39.75	36.75	56.50	38.38	51.38	86.75	75.00	33.38	58.88	38.00	39.13	50.51
chr5	140951494	140951607	DIAPH1	48.22	75.32	52.45	46.90	67.64	45.15	73.95	100.98	61.26	28.91	72.86	53.24	52.88	60.57
chr5	140953060	140953775	DIAPH1	25.48	37.39	28.02	32.22	36.46	29.70	44.25	52.58	45.72	58.16	38.44	27.89	33.07	38.03
chr5	140954535	140954713	DIAPH1	45.29	78.22	53.49	68.53	85.90	62.81	94.14	116.63	108.96	52.52	85.45	62.82	71.54	76.23
chr5	140955798	140955861	DIAPH1	83.19	151.61	92.44	77.56	127.44	79.41	140.91	210.88	166.94	8.14	102.94	91.50	73.13	111.08
chr5	140956324	140956438	DIAPH1	47.17	71.27	44.12	57.76	64.63	39.62	68.60	105.37	70.62	10.49	60.78	43.43	57.51	56.99
chr5	140957043	140957158	DIAPH1	61.47	75.46	49.16	53.18	87.62	41.51	79.70	134.38	92.81	7.12	71.13	29.40	60.84	65.24
chr5	140957793	140957910	DIAPH1	103.27	172.84	139.35	91.60	145.13	126.65	208.19	264.64	212.24	34.42	126.48	134.01	126.93	146.57
chr5	140958083	140958192	DIAPH1	19.82	23.15	25.55	16.28	33.67	25.34	30.55	56.75	36.80	12.47	22.20	23.67	28.25	27.19
chr5	140958656	140958763	DIAPH1	19.26	61.47	38.05	44.36	54.44	33.67	62.12	91.69	81.65	31.40	46.37	32.86	58.83	49.78
chr5	140960312	140960450	DIAPH1	40.22	44.88	32.85	49.99	68.25	35.68	45.53	56.29	58.38	29.31	39.16	28.67	41.91	44.10
chr5	140961880	140961942	DIAPH1	80.62	133.75	86.78	93.83	116.79	99.05	154.21	191.08	144.60	22.71	114.11	78.32	97.92	109.65
chr5	140962774	140962859	DIAPH1	48.95	64.45	57.90	63.85	80.97	58.80	79.66	121.60	98.86	36.43	78.62	73.21	67.76	71.94
chr5	140963053	140963182	DIAPH1	22.58	36.74	22.96	24.98	33.68	23.72	35.25	57.00	42.96	16.52	41.28	25.91	27.05	31.97
chr5	140963692	140963792	DIAPH1	18.87	16.64	24.80	14.67	31.36	22.10	32.14	47.18	35.12	5.61	21.42	22.95	22.00	24.41
chr5	140966610	140966764	DIAPH1	113.54	170.79	108.71	96.15	140.68	97.37	171.89	257.26	201.14	33.28	140.07	105.21	105.93	136.34
chr5	140967792	140967817	DIAPH1	69.23	87.77	41.38	54.92	71.08	37.46	59.96	81.96	71.65	27.88	64.96	34.88	76.08	58.60
chr5	140998366	140998481	DIAPH1	47.25	82.03	39.18	24.83	42.53	56.79	51.39	49.76	69.23	69.90	49.08	40.44	65.05	51.87
chr5	145239297	145239478	GRXCR2	33.89	55.49	27.99	46.69	72.80	31.84	46.79	70.13	51.41	84.48	42.21	26.98	49.11	49.22
chr5	145246065	145246291	GRXCR2	76.41	108.48	90.44	67.64	107.79	73.44	130.32	170.46	151.43	38.17	84.47	96.25	98.74	99.61
chr5	145252197	145252531	GRXCR2	66.13	110.42	80.54	77.20	112.81	69.44	117.18	139.87	120.70	106.72	77.22	68.80	84.59	95.59
chr5	145718677	145718795	POU4F3	52.88	41.49	33.56	67.18	81.84	42.97	62.09	66.18	43.71	132.77	62.13	44.01	66.29	60.90
chr5	145719112	145720007	POU4F3	108.59	125.65	90.12	127.20	198.10	91.87	130.04	187.75	153.17	282.24	124.35	83.10	137.64	141.85
chr6	2948533	2948933	SERPINB6	84.52	83.34	68.12	80.23	112.58	64.66	95.14	130.91	95.68	177.46	75.44	67.54	84.03	94.64
chr6	2949149	2949303	SERPINB6	73.25	116.41	85.28	83.17	112.75	63.19	131.39	154.95	144.40	47.39	73.29	92.21	89.94	98.14
chr6	2953279	2953420	SERPINB6	73.05	89.80	60.25	63.62	96.08	66.15	100.26	138.00	102.43	134.97	74.89	76.01	89.25	89.63
chr6	2954827	2954943	SERPINB6	110.01	166.40	108.35	107.97	128.05	85.77	175.77	246.30	185.35	43.55	137.50	84.57	99.36	131.63
chr6	2955759	2955904	SERPINB6	66.36	89.61	64.23	77.18	84.36	66.02	98.91	128.77	109.60	96.42	79.67	84.90	69.27	86.34
chr6	2959403	2959576	SERPINB6	48.44	73.15	54.56	68.16	100.02	52.10	115.87	143.25	81.01	109.68	65.89	65.29	73.24	81.45
chr6	2969014	2969147	SERPINB6	0.00	0.00	0.00	0.00	0.71	0.56	0.00	0.34	0.00	0.00	0.00	0.00	0.00	0.13
chr6	2970993	2971038	SERPINB6	0.00	0.00	2.00	0.00	2.28	0.00	0.00	0.00	0.00	0.00	0.00	0.00	0.00	0.36
chr6	2971352	2971382	SERPINB6	1.00	2.65	3.29	2.23	0.00	2.55	1.23	0.00	0.00	1.00	0.00	0.00	0.00	1.16
chr6	24806602	24806701	FAM65B	86.47	97.89	67.22	71.31	108.36	65.82	112.46	142.58	127.11	60.20	78.50	77.31	81.63	91.27
chr6	24809946	24810035	FAM65B	92.63	112.11	87.07	82.07	115.01	73.46	103.34	171.78	135.09	38.00	80.59	70.12	68.07	96.77
chr6	24818771	24818853	FAM65B	30.27	52.02	32.06	30.94	41.60	38.00	55.00	59.51	52.81	29.99	31.16	31.36	48.46	40.39
chr6	24825455	24825656	FAM65B	47.59	77.62	42.78	41.65	76.93	47.55	63.23	103.78	80.53	28.98	53.85	39.01	44.75	58.63
chr6	24828366	24828523	FAM65B	48.25	47.41	37.92	39.35	72.42	53.46	57.94	75.61	55.96	101.32	61.02	33.25	58.07	56.99
chr6	24830738	24830898	FAM65B	19.19	24.99	18.49	21.24	28.08	16.54	25.53	52.57	32.85	43.35	20.84	14.57	27.35	26.52
chr6	24832485	24832619	FAM65B	101.91	138.47	97.47	89.00	133.10	95.22	143.87	235.33	187.99	59.62	128.38	74.01	113.40	123.70
chr6	24835932	24836099	FAM65B	26.11	34.93	24.82	23.96	44.52	27.53	33.50	50.92	49.77	74.53	35.39	18.25	34.95	37.02
chr6	24839320	24839500	FAM65B	52.53	101.66	71.65	62.50	81.12	72.60	123.76	167.09	128.82	57.67	80.47	80.38	62.43	90.02
chr6	24839823	24839827	FAM65B	49.00	61.20	42.20	45.80	71.20	32.00	66.60	66.80	65.20	27.80	54.40	51.60	58.00	52.82
chr6	24840933	24841003	FAM65B	26.07	37.27	31.01	30.23	53.79	29.25	33.69	63.82	43.30	36.48	48.66	35.28	42.80	39.07
chr6	24843091	24843782	FAM65B	62.76	85.32	58.55	69.79	103.63	63.49	99.49	117.82	105.15	82.82	73.28	58.29	76.92	81.70
chr6	24847771	24847919	FAM65B	31.89	35.32	29.32	37.37	50.26	32.30	37.56	53.84	37.17	63.64	37.38	31.21	39.12	39.77
chr6	24848254	24848382	FAM65B	33.09	34.18	33.91	34.36	52.52	26.74	50.39	77.76	56.80	65.84	37.98	26.29	35.58	44.16
chr6	24850031	24850178	FAM65B	31.18	59.36	31.87	57.39	70.84	40.76	55.97	94.68	66.34	71.34	70.96	43.85	56.16	57.88
chr6	24850826	24850950	FAM65B	49.23	89.53	44.71	63.46	72.04	63.03	83.50	97.98	99.10	90.71	69.79	65.03	69.43	74.01
chr6	24852804	24852846	FAM65B	88.47	123.09	102.56	89.63	124.02	100.07	127.40	153.16	168.56	73.93	119.70	82.72	89.58	112.78
chr6	24861202	24861264	FAM65B	57.97	96.13	47.52	66.10	78.89	36.94	100.13	124.83	113.33	51.79	77.33	49.60	69.62	75.05
chr6	24865530	24865678	FAM65B	49.71	69.42	50.32	50.72	85.34	58.42	69.44	95.85	75.80	51.95	50.62	40.38	45.38	62.33
chr6	24869323	24869375	FAM65B	40.53	85.53	56.40	36.85	77.43	33.19	78.00	115.11	97.60	6.19	62.62	52.77	44.45	61.85
chr6	24871095	24871117	FAM65B	109.78	173.48	81.17	112.30	159.70	98.70	161.04	223.22	190.48	90.70	115.91	117.91	124.39	136.20
chr6	24873110	24873187	FAM65B	69.78	118.17	75.88	73.82	88.17	70.00	129.86	193.00	120.21	31.33	82.78	72.99	72.45	93.83
chr6	24873873	24874027	FAM65B	97.01	148.73	93.34	97.03	152.37	98.44	173.07	251.45	186.96	98.83	126.41	99.57	131.03	135.27
chr6	24875920	24876045	FAM65B	36.33	37.24	24.17	44.73	56.91	30.40	46.73	56.25	48.98	40.61	42.13	27.29	37.57	40.98
chr6	24936067	24936126	FAM65B	35.72	45.37	35.70	55.05	61.22	35.78	42.10	65.27	64.62	89.83	62.05	35.23	42.83	52.33
chr6	25042080	25042154	FAM65B	49.75	83.69	77.37	65.72	83.17	42.55	75.85	95.79	88.77	117.91	72.93	53.89	75.53	75.62
chr6	33131456	33131595	COL11A2	39.39	39.86	34.56	46.34	61.49	33.43	52.57	56.09	51.46	103.70	49.46	24.74	55.69	49.43
chr6	33132045	33132250	COL11A2	17.72	31.86	22.08	17.66	38.46	23.22	22.94	29.55	31.98	42.13	37.01	13.09	25.35	27.31
chr6	33132630	33132741	COL11A2	57.95	73.16	55.42	58.08	95.95	55.74	80.49	91.79	94.49	137.95	77.06	48.86	61.02	77.24
chr6	33133327	33133593	COL11A2	63.63	71.14	44.73	80.62	104.51	58.65	63.25	106.70	87.88	163.09	67.12	43.32	73.37	79.55
chr6	33133709	33133761	COL11A2	49.77	57.96	44.21	49.55	76.53	46.49	62.83	80.94	82.57	110.62	54.21	52.00	49.34	63.97
chr6	33133949	33133983	COL11A2	25.00	40.89	45.46	41.09	65.83	32.66	60.29	55.46	57.51	83.54	52.43	36.03	47.20	49.68
chr6	33134291	33134343	COL11A2	55.89	49.81	32.09	41.55	62.23	33.02	46.47	81.26	50.66	102.40	45.79	36.70	49.15	53.16
chr6	33134498	33134604	COL11A2	21.83	29.15	11.80	28.64	48.08	18.05	28.20	37.24	36.84	62.50	27.75	33.35	35.26	31.95
chr6	33134848	33134900	COL11A2	14.57	15.58	14.66	14.98	26.57	16.66	21.62	21.70	13.19	53.34	19.62	14.19	21.96	20.56
chr6	33135042	33135094	COL11A2	33.51	39.28	24.77	39.92	80.58	38.79	39.75	59.13	33.34	97.51	38.06	26.09	49.68	45.90
chr6	33135204	33135310	COL11A2	47.15	40.94	24.56	42.66	66.03	33.68	60.25	80.45	68.09	110.12	36.20	35.93	62.96	53.84
chr6	33135577	33135629	COL11A2	27.13	26.85	25.66	27.28	44.58	19.49	43.26	46.98	35.45	71.68	36.49	20.32	39.34	35.43
chr6	33136297	33136349	COL11A2	24.96	37.30	20.60	37.64	53.15	23.06	35.23	47.38	32.77	92.21	24.23	23.87	36.32	37.70
chr6	33136484	33136536	COL11A2	14.64	23.15	15.74	31.58	33.96	14.49	22.53	34.72	28.13	55.06	28.98	17.13	26.04	26.68

chr6	33136733	33136785	COL11A2	21.49	28.66	19.77	22.45	34.36	21.96	34.89	39.91	39.98	63.58	29.28	25.30	35.04	31.80
chr6	33137161	33137267	COL11A2	30.10	52.18	43.71	45.35	68.35	39.32	45.05	58.05	69.79	94.94	59.63	45.10	52.35	54.30
chr6	33137609	33137661	COL11A2	44.51	69.58	45.21	43.11	90.72	45.26	63.62	108.08	84.42	105.66	49.87	43.81	59.96	66.15
chr6	33137825	33137877	COL11A2	54.79	65.40	51.00	60.68	86.49	50.38	86.87	117.64	85.02	127.40	75.53	49.40	53.83	75.88
chr6	33138104	33138156	COL11A2	38.26	59.47	43.38	54.77	81.68	39.36	57.09	94.19	55.87	107.60	43.43	42.43	67.92	59.80
chr6	33138335	33138387	COL11A2	27.72	38.83	32.42	34.21	52.40	34.60	40.34	54.68	41.94	54.51	42.77	32.51	49.96	40.58
chr6	33138588	33138694	COL11A2	52.13	72.49	66.88	70.21	112.96	72.23	68.22	101.49	91.47	156.58	68.88	58.08	72.18	82.64
chr6	33138892	33138944	COL11A2	32.45	44.11	31.43	34.17	57.28	31.57	43.53	59.74	47.64	81.57	37.08	30.68	40.72	44.27
chr6	33139049	33139101	COL11A2	43.94	46.74	49.51	45.04	61.83	35.89	64.06	103.08	59.60	101.87	48.34	40.75	44.77	58.39
chr6	33139245	33139351	COL11A2	32.36	44.03	32.73	47.22	63.25	47.33	46.18	71.35	50.49	84.44	32.10	27.98	37.77	48.29
chr6	33139491	33139597	COL11A2	67.52	85.47	52.19	70.75	109.65	55.72	91.05	116.69	100.79	147.07	75.06	57.21	62.32	85.76
chr6	33139828	33139880	COL11A2	50.43	43.89	24.81	40.75	74.36	34.26	50.75	73.30	65.81	81.42	44.53	26.87	45.83	50.93
chr6	33140067	33140155	COL11A2	36.55	59.63	38.08	49.49	56.37	40.20	65.03	67.38	70.45	80.06	57.97	42.11	53.11	55.28
chr6	33140308	33140414	COL11A2	37.23	49.79	49.81	50.27	69.65	43.96	53.79	64.78	64.70	110.15	47.43	46.26	52.72	57.32
chr6	33140838	33140890	COL11A2	48.36	63.42	41.15	54.62	82.45	38.09	54.89	86.79	59.47	134.68	56.57	41.53	72.75	63.50
chr6	33141126	33141178	COL11A2	28.81	22.87	18.64	29.34	39.51	22.70	31.89	30.23	26.64	61.74	30.15	21.74	27.04	30.35
chr6	33141280	33141332	COL11A2	21.83	20.45	17.62	19.30	24.34	19.89	27.13	21.66	22.15	43.19	27.42	22.55	14.91	23.96
chr6	33141479	33141522	COL11A2	36.61	43.82	41.00	49.02	63.64	44.30	55.25	68.91	47.64	92.34	26.32	38.75	47.25	50.63
chr6	33141651	33141703	COL11A2	81.58	93.43	69.15	74.36	120.26	70.15	102.36	109.89	114.70	170.11	72.13	58.79	87.23	94.74
chr6	33141789	33141832	COL11A2	94.34	94.48	74.25	66.89	118.61	66.36	96.00	94.91	113.41	184.89	109.84	54.86	121.75	97.40
chr6	33141943	33141995	COL11A2	28.70	49.15	31.91	29.83	51.51	37.89	46.15	45.36	52.66	71.47	40.38	27.19	59.15	42.68
chr6	33142305	33142357	COL11A2	25.89	50.17	29.40	30.58	59.49	24.96	31.09	47.09	51.89	52.26	36.85	24.26	33.68	38.66
chr6	33143352	33143458	COL11A2	44.98	49.16	42.90	46.48	69.52	46.63	44.71	73.28	52.99	105.30	45.47	25.35	45.90	53.90
chr6	33143794	33143846	COL11A2	54.04	69.30	41.36	65.34	74.11	40.36	56.21	99.68	75.19	116.66	60.32	36.08	63.26	65.72
chr6	33144037	33144080	COL11A2	17.09	33.93	14.80	11.16	21.32	16.70	29.77	36.16	33.61	37.02	25.95	22.23	34.16	24.98
chr6	33144211	33144263	COL11A2	26.17	26.04	18.66	20.32	28.94	27.92	29.96	34.38	28.55	40.09	22.68	18.94	24.98	26.89
chr6	33144499	33144542	COL11A2	11.36	10.18	22.55	21.36	13.86	25.05	22.55	23.66	13.05	12.45	17.43	11.09	13.75	17.05
chr6	33144770	33144822	COL11A2	34.28	38.28	38.53	48.94	59.58	37.87	40.89	60.91	49.38	72.30	32.58	26.25	48.06	44.98
chr6	33144959	33145002	COL11A2	62.61	84.75	57.27	60.59	100.05	62.05	94.52	105.89	125.59	124.73	95.23	56.02	88.68	85.77
chr6	33145190	33145242	COL11A2	46.30	58.40	29.58	43.26	67.66	28.19	56.47	76.38	65.92	75.62	43.00	39.43	48.60	52.52
chr6	33145440	33145483	COL11A2	40.43	71.80	51.98	57.30	84.86	40.89	77.50	92.84	85.18	138.14	63.05	49.20	61.98	71.10
chr6	33145910	33145962	COL11A2	54.83	64.85	41.57	49.58	69.75	42.53	72.43	97.66	71.94	125.30	55.75	42.74	63.92	65.75
chr6	33146086	33146129	COL11A2	67.00	80.34	62.50	59.36	108.84	53.30	87.73	107.68	102.57	146.14	66.95	75.00	75.61	84.78
chr6	33146213	33146265	COL11A2	67.87	68.13	47.15	54.64	89.28	69.45	76.89	87.87	88.40	138.08	64.28	75.47	71.85	77.29
chr6	33146457	33146509	COL11A2	75.38	107.21	83.08	84.74	108.58	72.53	115.04	151.11	118.89	164.72	87.34	80.62	95.75	104.10
chr6	33146698	33146750	COL11A2	43.40	61.85	40.06	52.21	82.36	42.40	48.92	70.64	65.83	98.04	46.49	34.83	51.42	57.25
chr6	33146851	33146903	COL11A2	35.38	46.38	70.83	50.55	38.13	111.94	45.25	71.51	61.36	56.32	28.51	29.60	38.13	53.81
chr6	33147009	33147061	COL11A2	69.13	70.26	51.43	60.42	90.02	45.08	70.85	88.74	78.53	138.72	72.47	60.43	82.17	74.67
chr6	33147209	33147264	COL11A2	88.55	81.70	73.63	104.75	125.05	66.43	104.52	125.64	102.75	234.55	94.04	68.13	108.16	105.81
chr6	33147497	33147582	COL11A2	33.01	42.91	26.65	37.09	71.13	34.33	48.17	53.36	52.37	67.64	52.02	43.35	34.08	46.84
chr6	33148036	33148109	COL11A2	26.80	31.00	16.82	18.11	41.08	19.20	37.28	36.85	51.58	39.86	38.61	19.59	31.69	31.40
chr6	33148446	33148507	COL11A2	16.34	29.21	15.29	24.13	34.19	20.40	25.65	41.44	29.60	44.26	26.15	15.87	23.47	26.88
chr6	33148742	33148782	COL11A2	32.78	48.34	31.63	41.00	40.20	46.95	43.07	60.05	46.41	67.63	56.27	32.63	42.05	45.58
chr6	33148889	33148947	COL11A2	65.73	67.76	53.93	67.85	83.02	62.44	69.53	106.00	83.86	111.73	72.85	50.31	77.58	74.58
chr6	33151923	33152101	COL11A2	65.03	84.05	54.42	47.74	85.67	58.65	75.97	96.16	83.58	102.58	66.70	50.30	74.30	72.57
chr6	33152770	33152831	COL11A2	22.03	26.85	23.19	26.31	45.47	19.23	25.00	36.92	29.44	65.35	22.02	15.16	21.02	29.75
chr6	33153479	33153555	COL11A2	17.83	20.43	12.01	10.13	20.45	13.51	23.01	41.78	20.58	23.57	29.25	11.18	20.78	20.31
chr6	33154330	33154595	COL11A2	52.24	69.54	45.35	49.50	70.30	47.11	85.00	106.23	76.73	96.53	58.18	43.33	71.08	66.67
chr6	33156140	33156301	COL11A2	32.59	39.19	34.10	36.98	52.25	25.54	49.75	59.91	50.59	61.64	42.94	25.91	36.22	42.62
chr6	33156756	33156965	COL11A2	42.40	62.28	36.37	52.14	74.74	40.81	57.41	84.61	66.76	85.34	50.20	47.85	43.77	58.41
chr6	33157098	33157246	COL11A2	54.80	87.27	57.80	82.81	123.54	58.59	72.83	110.48	88.44	153.28	76.22	51.70	70.26	84.81
chr6	33159937	33160017	COL11A2	16.75	15.31	17.58	17.53	43.96	16.22	16.51	19.40	21.30	57.28	18.11	14.25	21.56	22.85
chr6	35773449	35773859	LHFPL5	104.68	105.98	91.06	111.82	173.80	103.72	121.88	161.67	137.58	241.19	110.96	85.19	132.51	129.13
chr6	35782324	35782559	LHFPL5	83.32	83.47	55.28	78.02	126.50	51.31	86.97	123.22	86.65	198.32	93.43	56.54	88.05	93.59
chr6	35787215	35787240	LHFPL5	52.38	106.88	61.92	54.12	88.08	49.69	93.58	155.62	112.65	47.96	63.35	57.54	61.88	78.65
chr6	76527219	76527381	MYO6	46.12	92.55	50.85	45.37	84.23	53.69	93.82	99.25	106.83	45.21	66.28	48.86	53.09	69.42
chr6	76532500	76532568	MYO6	36.29	67.83	49.80	35.35	44.83	35.59	56.03	57.55	75.99	2.00	40.67	35.48	32.65	44.78
chr6	76538258	76538330	MYO6	22.07	51.68	40.66	16.63	32.77	25.62	41.56	68.86	55.77	0.53	24.41	27.64	22.40	34.02
chr6	76540134	76540262	MYO6	26.92	51.91	45.36	32.16	39.22	25.47	49.01	61.62	81.47	0.00	34.30	39.48	35.83	40.58
chr6	76542560	76542664	MYO6	52.31	98.46	60.69	27.28	57.10	66.21	77.46	86.44	104.32	5.67	67.42	49.16	49.06	62.71
chr6	76545619	76545673	MYO6	25.00	35.58	26.91	25.49	43.85	22.71	42.62	65.91	56.00	0.00	30.93	23.95	25.05	33.25
chr6	76550303	76550399	MYO6	35.34	52.96	35.03	28.76	45.40	37.37	49.54	84.72	74.35	18.37	37.59	42.84	29.30	45.19
chr6	76550932	76551095	MYO6	9.87	36.84	27.05	11.43	28.56	20.18	34.05	31.02	36.75	1.65	21.55	24.45	18.94	23.62
chr6	76554615	76554694	MYO6	36.68	73.40	47.56	30.63	43.80	35.50	71.13	75.93	79.31	1.00	48.49	43.91	37.74	48.94
chr6	76558069	76558248	MYO6	85.72	143.87	102.36	72.87	105.26	90.11	154.04	191.27	158.69	33.06	98.04	75.03	97.07	109.19
chr6	76564857	76565000	MYO6	78.85	87.03	82.67	72.62	88.79	72.81	111.82	129.03	117.23	11.97	90.87	73.60	91.42	84.77
chr6	76566815	76566971	MYO6	73.31	104.83	67.65	52.72	98.47	67.35	103.20	182.80	139.45	5.25	75.54	68.07	65.16	86.55
chr6	76568620	76568710	MYO6	23.86	52.02	28.92	25.44	22.25	33.53	47.98	60.41	44.09	3.11	26.95	19.37	24.04	32.33
chr6	76570741	76570812	MYO6	40.51	64.58	54.13	28.57	43.13	40.11	70.32	115.22	68.86	2.15	43.18	43.86	37.79	51.22
chr6	76572314	76572440	MYO6	101.02	164.98	94.46	96.76	139.96	91.66	190.83	248.09	210.67	30.01	113.87	93.88	104.41	131.35
chr6	76576244	76576338	MYO6	122.64	191.55	118.16	105.71	147.17	91.63	160.25	203.02	200.66	20.52	129.39	94.45	110.71	132.10
chr6	76576650	76576822	MYO6	24.74	38.23	39.72	14.83	28.92	21.91	43.35	44.84	43.29	2.59	26.71	18.73	27.06	28.99
chr6	76580365	76580402	MYO6	14.29	33.00	29.74	17.74	22.05	24.13	41.68	51.45	43.18	0.00	28.68	29.68	18.26	27.97
chr6	76582925	76583017	MYO6	62.92	144.46	89.32	78.39	85.44	74.06	139.27	188.27	152.39	5.94	96.17	95.17	68.00	100.98
chr6	76589538	76589667	MYO6	53.48	120.79	66.15	54.06	76.01	55.22	111.69	146.55	133.05	16.67	81.55	64.02	50.43	81.60
chr6	76589761	76589837	MYO6	3.75	24.01	15.00	11.03	8.62	16.92	27.26	24.68	25.78	1.88	13.25	21.01	14.73	16.10
chr6	76591407	76591535	MYO6	64.88	106.98	62.65	64.85	93.96	62.84	87.67	135.90	94.74	17.34	87.10	53.61	70.73	77.71
chr6	76595722	76595811	MYO6	16.06	28.09	18.61	16.79	15.07	18.88	19.63	25.31	33.56	0.00	14.23	14.77	19.09	18.42
chr6	76596562	76596711	MYO6	31.23	75.17	40.21	23.65	44.49	40.19	67.46	78.61	70.23	1.11	40.11	32.61	39.04	45.42
chr6	76599775	76599982	MYO6	43.21	52.26	46.50	50.33	66.03	38.28	52.81	46.12	50.36	31.75	50.53	33.64	63.03	46.82

chr6	76600946	76601023	MYO6	30.24	50.77	33.82	37.60	47.59	19.38	46.99	45.03	49.94	3.27	52.22	28.72	33.92	37.13
chr6	76602248	76602407	MYO6	24.83	24.60	22.37	23.76	38.98	20.81	34.94	36.29	31.73	42.04	23.62	21.58	31.21	28.80
chr6	76604949	76604977	MYO6	39.66	77.69	39.48	33.17	44.45	51.00	55.59	90.72	74.62	0.00	43.48	46.97	46.59	49.74
chr6	76608091	76608128	MYO6	23.68	38.05	27.37	20.71	26.89	22.92	34.97	58.58	40.34	3.00	25.39	26.66	26.47	29.05
chr6	76617323	76617425	MYO6	77.17	131.24	84.70	72.02	92.35	55.34	116.87	179.41	148.04	11.16	101.49	60.46	90.44	94.19
chr6	76618214	76618344	MYO6	50.48	102.84	66.11	50.47	76.60	52.46	86.54	145.44	114.38	0.86	57.93	55.97	53.86	71.67
chr6	76621390	76621415	MYO6	21.81	58.88	38.88	18.46	39.50	37.69	44.08	89.08	73.38	2.00	35.38	34.88	26.85	41.17
chr6	76623781	76623998	MYO6	38.03	59.68	31.14	40.94	44.00	38.36	54.22	58.73	53.69	58.70	45.76	31.93	43.61	46.27
chr6	76624531	76624729	MYO6	17.50	37.41	19.39	22.26	28.40	19.41	25.20	40.09	35.13	27.15	23.16	10.77	20.52	25.49
chr6	133595855	133595951	EYA4	38.18	52.90	42.48	41.68	56.97	42.37	63.06	77.77	60.15	1.53	54.95	34.95	53.61	47.25
chr6	133703531	133703579	EYA4	50.53	84.90	43.29	60.65	62.55	38.94	91.24	104.84	89.55	0.69	46.69	39.57	46.06	59.45
chr6	133767769	133767892	EYA4	47.61	77.67	56.12	42.58	77.50	38.16	72.55	114.53	100.61	5.46	51.16	48.73	44.66	61.06
chr6	133769250	133769317	EYA4	62.22	124.22	87.68	86.46	88.99	64.07	107.37	190.44	146.21	29.35	93.90	73.35	77.35	96.19
chr6	133777695	133777786	EYA4	79.52	122.40	69.76	70.67	133.73	48.84	100.75	146.23	112.51	79.55	101.53	80.24	90.91	95.48
chr6	133782253	133782318	EYA4	71.95	156.27	126.26	109.82	150.09	83.59	205.33	270.23	200.95	3.56	119.68	110.74	102.52	134.04
chr6	133783474	133783615	EYA4	96.11	171.09	100.83	108.11	149.52	106.86	137.70	214.28	187.95	92.94	113.46	95.87	97.82	131.23
chr6	133783760	133783902	EYA4	47.52	83.07	55.25	37.34	68.05	46.78	76.10	106.61	77.94	43.00	56.85	44.18	57.06	61.89
chr6	133785918	133785996	EYA4	31.49	80.38	50.56	23.96	54.82	49.82	77.80	106.65	80.44	1.00	42.47	53.54	35.73	54.41
chr6	133789705	133789887	EYA4	71.95	136.89	81.99	70.85	97.30	78.57	129.89	181.84	138.19	27.49	81.28	80.72	88.01	98.08
chr6	133802602	133802737	EYA4	32.26	32.13	24.00	25.36	24.62	19.76	36.84	42.42	42.56	3.77	29.22	25.46	30.35	28.20
chr6	133804171	133804253	EYA4	107.63	239.11	127.54	84.65	118.06	104.46	206.93	269.63	256.37	38.75	129.43	136.19	88.66	151.56
chr6	133827245	133827333	EYA4	34.09	67.19	49.92	34.08	55.01	49.47	64.19	65.87	81.64	7.81	38.51	37.82	41.94	48.80
chr6	133833860	133833917	EYA4	68.29	145.71	79.93	64.31	111.81	86.74	127.72	206.84	176.72	10.88	93.10	81.26	74.81	104.44
chr6	133834017	133834176	EYA4	86.91	144.49	84.97	72.53	115.00	64.64	139.95	187.41	147.73	50.57	96.88	88.62	78.28	106.64
chr6	133836460	133836573	EYA4	49.95	96.04	58.14	67.31	83.22	56.77	87.23	107.41	91.92	14.21	75.37	57.05	74.79	70.39
chr6	133844195	133844315	EYA4	53.78	94.35	53.85	43.44	86.82	72.21	88.90	109.26	119.85	0.62	73.03	59.61	67.66	71.31
chr6	133846154	133846253	EYA4	70.05	109.16	67.38	75.59	68.03	67.58	99.91	170.19	116.85	33.33	74.07	54.84	75.33	83.92
chr6	133846293	133846392	EYA4	95.24	162.61	89.64	88.99	117.69	76.47	129.29	222.22	168.96	47.70	106.87	68.84	101.36	114.54
chr6	133849864	133849943	EYA4	56.36	79.88	56.34	54.44	77.89	59.89	87.46	122.96	101.45	43.78	61.35	54.21	59.23	71.33
chr7	24738646	24738878	DFNA5	60.31	86.82	64.35	53.47	97.58	47.24	100.64	120.64	100.98	28.49	70.35	47.78	71.42	73.22
chr7	24742380	24742452	DFNA5	25.93	30.42	19.30	27.40	44.68	18.18	42.00	61.33	52.16	44.67	35.34	34.42	23.93	36.32
chr7	24745804	24745995	DFNA5	16.37	32.36	15.72	22.30	29.21	20.61	28.36	51.12	37.35	41.07	28.63	25.16	23.28	29.02
chr7	24747747	24747873	DFNA5	23.56	43.23	33.39	35.20	45.21	49.53	33.24	42.34	42.67	62.43	63.82	49.05	29.79	33.57	42.35
chr7	24749844	24750007	DFNA5	18.57	32.10	26.58	27.58	36.03	17.87	33.00	44.75	42.13	43.44	26.41	15.79	27.37	30.35
chr7	24756874	24756993	DFNA5	63.32	100.86	78.88	104.63	171.08	102.85	118.02	172.13	112.88	250.91	109.87	73.77	130.67	121.60
chr7	24758667	24758837	DFNA5	27.90	22.37	49.37	35.35	24.77	48.07	35.07	41.87	35.07	23.80	17.49	25.57	16.63	32.22
chr7	24784182	24784373	DFNA5	53.67	82.87	59.30	57.86	89.57	57.84	85.87	120.87	96.40	125.89	60.61	63.79	61.98	79.54
chr7	24789184	24789412	DFNA5	42.42	65.99	33.57	33.83	55.49	32.87	60.57	88.99	82.22	20.80	50.64	36.61	42.72	50.33
chr7	45614144	45614781	ADCY1	28.81	22.03	14.77	28.04	45.73	18.30	27.58	36.63	34.91	53.11	26.29	15.22	31.40	29.28
chr7	45632359	45632507	ADCY1	112.47	129.40	88.48	142.85	202.29	87.08	167.11	233.82	146.25	287.19	138.27	78.77	121.15	151.16
chr7	45649979	45650096	ADCY1	104.09	112.05	78.78	69.95	125.06	70.40	114.22	182.39	107.49	192.98	105.02	63.08	106.00	110.46
chr7	45662232	45662342	ADCY1	30.20	42.61	28.53	24.77	45.21	22.80	38.64	66.77	55.82	59.07	31.53	29.72	36.06	39.64
chr7	45688270	45688396	ADCY1	77.80	110.94	67.21	80.19	112.00	80.86	109.79	170.65	119.62	174.16	107.20	65.50	94.21	106.33
chr7	45697327	45697484	ADCY1	45.72	38.33	22.86	37.03	66.16	27.43	42.32	60.18	56.73	95.22	40.08	25.58	51.94	46.47
chr7	45699642	45699782	ADCY1	49.65	98.21	62.28	81.14	96.77	67.62	87.97	142.30	99.54	122.54	71.78	54.53	63.70	86.19
chr7	45701659	45701813	ADCY1	65.30	92.72	57.52	68.09	90.23	51.38	91.17	140.62	114.70	101.83	80.70	73.26	80.04	85.63
chr7	45703889	45703974	ADCY1	31.78	39.65	26.01	38.10	61.49	37.44	52.71	72.85	49.99	92.03	41.67	24.86	50.66	47.38
chr7	45717469	45717662	ADCY1	100.98	147.23	93.15	99.76	165.56	89.20	136.57	164.84	143.16	210.00	113.25	92.16	107.03	129.66
chr7	45717766	45717862	ADCY1	53.31	77.68	48.43	60.41	99.91	40.05	77.23	111.07	64.82	120.44	76.82	39.80	60.65	72.50
chr7	45719309	45719392	ADCY1	65.07	108.48	71.56	70.90	113.95	77.90	119.29	190.10	140.17	132.63	82.19	53.48	69.27	102.14
chr7	45724579	45724667	ADCY1	70.61	93.98	62.04	60.58	96.37	69.42	110.82	169.18	115.18	71.36	88.85	55.64	75.28	88.67
chr7	45725562	45725814	ADCY1	66.11	78.26	58.01	71.26	112.35	47.34	82.35	105.92	92.67	167.44	81.14	43.99	75.50	83.90
chr7	45726147	45726272	ADCY1	56.93	50.02	27.62	55.76	88.50	28.90	57.98	69.85	60.61	122.71	50.38	46.43	56.82	59.64
chr7	45742976	45743091	ADCY1	34.74	35.19	23.80	44.14	56.35	39.30	45.47	60.88	37.02	82.52	47.85	35.09	42.24	45.20
chr7	45743200	45743345	ADCY1	125.32	125.24	86.71	145.52	187.42	105.62	144.47	215.08	140.55	297.35	143.12	98.93	148.95	151.28
chr7	45744118	45744215	ADCY1	21.57	51.58	33.45	32.83	38.21	34.58	47.00	67.74	56.05	75.14	36.38	35.61	34.81	44.18
chr7	45747950	45748063	ADCY1	38.21	66.74	46.22	45.18	75.47	38.88	67.00	107.61	79.52	63.48	48.51	35.98	42.41	59.40
chr7	45750128	45750251	ADCY1	76.65	105.34	65.33	83.60	107.23	59.34	89.93	142.19	125.15	185.65	89.50	55.62	69.66	98.79
chr7	45753293	45753594	ADCY1	56.69	74.54	43.70	63.56	77.40	53.69	73.29	114.25	74.28	138.03	77.92	49.90	76.80	74.77
chr7	81331898	81332073	HGF	162.02	271.25	172.89	146.75	202.39	120.70	229.49	300.44	261.01	55.43	179.17	152.08	165.62	187.80
chr7	81334707	81334851	HGF	38.86	73.16	35.72	39.36	59.39	47.61	72.08	93.04	81.77	2.79	34.72	46.24	37.34	52.06
chr7	81334964	81335069	HGF	54.05	106.84	49.24	55.92	88.10	54.06	103.26	99.21	110.20	13.58	67.43	51.64	61.22	71.13
chr7	81335604	81335743	HGF	110.86	190.39	134.80	124.06	156.85	120.82	198.04	246.54	238.80	12.95	162.10	123.96	126.77	151.68
chr7	81336607	81336680	HGF	34.26	70.14	40.53	31.39	50.42	51.11	62.49	64.38	87.59	1.91	41.46	35.27	39.23	47.58
chr7	81339464	81339559	HGF	40.89	79.63	37.05	31.20	70.73	42.60	75.54	113.36	87.81	11.95	41.41	38.39	46.90	55.88
chr7	81340798	81340835	HGF	10.76	27.45	21.45	18.45	20.03	22.95	24.95	18.97	33.95	0.00	20.37	13.61	8.74	19.41
chr7	81346549	81346681	HGF	78.49	124.30	87.30	83.68	119.95	82.29	119.72	165.53	124.50	38.71	115.90	75.56	87.72	101.33
chr7	81350062	81350163	HGF	29.52	55.60	21.26	24.79	40.76	27.14	59.30	56.37	65.55	0.00	36.80	23.70	25.92	36.73
chr7	81355207	81355333	HGF	71.77	128.22	90.77	76.04	118.54	80.42	169.57	222.98	177.68	66.38	92.46	93.38	87.99	115.68
chr7	81358922	81359095	HGF	40.40	80.74	56.09	41.29	73.03	48.53	78.80	123.28	99.01	19.74	54.87	62.29	62.78	64.84
chr7	81372259	81372265	HGF	24.57	34.29	44.86	30.29	45.00	5.57	37.14	70.71	52.43	35.14	28.71	35.00	30.29	36.98
chr7	81372670	81372787	HGF	48.10	59.76	40.06	61.88	67.25	51.97	74.20	133.58	86.96	91.50	66.35	55.29	64.97	69.74
chr7	81374317	81374436	HGF	69.60	107.65	57.08	45.84	77.38	65.63	100.79	156.28	109.53	23.64	72.38	46.32	66.76	77.68
chr7	81381429	81381578	HGF	92.24	153.93	92.20	92.41	117.45	80.57	141.34	214.62	158.53	35.79	114.13	75.32	107.43	114.04
chr7	81386506	81386619	HGF	90.04	156.92	84.55	82.90	99.67	80.83	150.11	208.96	167.29	0.82	104.51	80.04	93.97	108.89
chr7	81388009	81388120	HGF	26.11	55.75	45.94	24.77	33.95	25.71	55.33	53.79	66.67	0.00	28.28	23.16	29.18	36.62
chr7	81392024	81392188	HGF	34.18	53.54	34.81	21.96	25.70	31.12	59.44	75.07	76.79	0.00	38.70	30.73	29.34	40.17
chr7	81399201	81399287	HGF	27.97	40.98	18.40	28.69	37.01	26.46	38.98	57.11	56.56	22.24	29.33	19.89	32.95	33.64
chr7	107302085	107302250	SLC26A4	26.75	32.59	16.78	44.94	64.74	24.55	43.42	48.83	38.60	71.22	30.57	18.61	29.75	38.47
chr7	107303742	107303880	SLC26A4	54.23	99.19	54.14	66.34	101.04	55.15	111.25	141.46	103.26	81.51	73.50	61.05	67.55	83.51

chr7	107312584	107312693	SLC26A4	54.73	116.59	73.96	49.05	85.76	58.29	117.53	159.19	134.95	0.88	60.78	63.57	56.92	81.27
chr7	107314610	107314793	SLC26A4	39.09	47.03	48.74	34.54	50.47	37.47	71.49	72.08	73.04	11.10	53.05	42.20	38.78	48.36
chr7	107315391	107315554	SLC26A4	69.96	109.50	61.31	56.03	68.27	58.43	118.26	159.28	140.81	25.93	72.72	55.23	56.99	82.98
chr7	107323648	107323799	SLC26A4	25.05	40.11	35.72	19.96	29.32	29.43	50.72	63.26	60.44	1.98	23.47	33.23	32.73	34.39
chr7	107323901	107323982	SLC26A4	39.43	65.02	60.32	45.26	61.67	51.89	81.82	118.12	94.22	4.22	63.98	58.02	54.10	62.00
chr7	107329499	107329645	SLC26A4	54.50	83.59	44.31	64.87	90.56	56.97	87.52	152.42	105.88	63.91	66.03	60.61	72.54	77.60
chr7	107330570	107330682	SLC26A4	78.21	136.75	81.77	89.18	111.26	99.99	136.72	208.99	171.77	95.84	100.16	82.37	84.27	116.08
chr7	107334849	107334925	SLC26A4	69.79	86.03	52.29	51.78	83.81	53.26	91.38	150.30	120.52	53.90	71.58	61.70	75.23	78.86
chr7	107335067	107335161	SLC26A4	80.13	154.80	80.23	88.17	133.58	86.46	136.00	201.27	165.97	49.47	109.07	91.29	97.96	114.70
chr7	107336379	107336484	SLC26A4	64.81	136.68	76.33	60.78	83.49	55.18	121.26	153.68	118.37	8.25	85.25	78.70	62.75	86.90
chr7	107338488	107338556	SLC26A4	74.84	152.12	72.68	81.32	112.13	69.36	156.38	179.81	145.20	50.57	87.67	80.19	96.96	105.19
chr7	107340529	107340620	SLC26A4	20.92	66.28	22.57	23.37	32.73	29.97	46.61	61.57	65.08	2.09	26.71	38.33	27.50	36.35
chr7	107341547	107341641	SLC26A4	41.11	71.08	38.43	36.11	56.22	36.80	80.44	83.94	87.34	0.00	43.16	40.94	41.76	51.30
chr7	107342273	107342502	SLC26A4	98.54	154.55	100.82	93.16	130.89	84.57	156.30	257.20	172.37	48.81	98.09	88.99	102.42	123.69
chr7	107344777	107344830	SLC26A4	59.54	132.39	98.98	54.83	81.17	74.44	126.24	192.41	157.67	1.96	78.02	89.54	56.30	95.60
chr7	107350500	107350644	SLC26A4	46.03	85.17	45.56	43.11	62.86	49.71	79.59	111.99	98.68	8.46	49.74	57.90	43.91	61.57
chr7	107352985	107353067	SLC26A4	41.58	91.93	64.94	46.02	69.19	42.58	106.99	114.18	99.47	3.59	56.51	54.67	53.83	65.97
chr7	107355869	107355891	SLC26A4	98.61	154.61	104.61	102.57	115.83	105.52	154.91	172.30	171.52	16.00	115.43	72.43	98.57	115.36
chr7	116339126	116340338	MET	115.49	188.92	129.68	118.17	164.14	119.56	207.31	293.03	224.20	91.42	133.43	124.87	126.84	159.18
chr7	116371723	116371913	MET	52.51	80.10	50.64	40.11	64.34	64.48	70.10	117.69	80.67	36.06	58.17	52.61	40.13	63.96
chr7	116380005	116380138	MET	72.82	107.34	70.34	77.59	106.70	71.57	128.87	192.19	144.05	48.34	95.47	63.96	94.44	98.27
chr7	116380907	116381079	MET	32.06	27.73	28.29	14.94	52.57	29.39	37.35	49.61	33.29	65.23	37.72	28.94	35.97	36.43
chr7	116395410	116395569	MET	69.60	139.16	87.39	70.23	99.89	67.43	116.23	121.26	148.30	35.44	100.16	57.28	84.04	92.70
chr7	116397492	116397593	MET	27.16	34.57	28.16	21.22	23.87	25.36	49.60	34.15	52.67	0.00	27.56	23.75	21.25	29.00
chr7	116397693	116397828	MET	54.50	99.15	52.25	49.00	62.99	71.60	85.34	118.50	125.11	8.42	40.12	45.93	49.96	67.74
chr7	116398514	116398674	MET	44.12	94.94	70.97	47.14	64.55	58.92	102.99	127.77	123.69	6.34	61.24	51.74	49.39	71.20
chr7	116399392	116399544	MET	106.98	154.25	94.23	101.49	145.65	98.84	173.50	259.20	189.63	50.01	118.96	108.30	110.30	133.42
chr7	116403105	116403322	MET	102.33	277.79	172.31	119.55	161.56	135.85	277.28	381.06	294.06	19.36	146.61	154.24	136.06	186.83
chr7	116409700	116409920	MET	57.70	104.06	72.14	59.71	98.11	61.88	124.58	142.53	130.74	31.87	73.02	76.87	74.61	86.10
chr7	116411553	116411708	MET	24.35	43.46	25.83	25.11	35.44	23.73	41.06	72.51	44.85	6.14	29.46	21.94	23.19	32.82
chr7	116411904	116412043	MET	68.51	97.92	49.57	61.04	88.59	55.88	104.91	148.00	123.98	28.79	78.74	68.67	66.27	81.22
chr7	116414936	116415165	MET	40.30	69.61	54.73	48.83	76.70	46.67	73.52	119.53	89.53	20.95	83.04	45.08	61.87	64.04
chr7	116417444	116417523	MET	46.01	115.53	79.98	51.94	87.39	70.74	109.25	138.99	129.45	15.41	81.50	63.75	61.06	80.20
chr7	116418831	116419011	MET	24.65	43.92	34.38	26.27	44.19	22.31	46.27	59.88	47.02	17.66	28.76	24.81	26.35	35.01
chr7	116422043	116422151	MET	68.72	107.39	74.01	52.73	85.52	60.12	118.97	157.98	111.04	10.45	71.28	71.81	63.51	82.50
chr7	116423359	116423523	MET	89.38	116.97	81.84	86.34	108.90	72.58	108.60	179.10	144.44	36.44	91.16	82.54	79.18	99.86
chr7	116435710	116435845	MET	29.82	39.81	43.12	34.03	44.13	30.32	38.58	77.82	62.20	19.06	40.64	38.74	36.85	41.52
chr7	116435942	116436178	MET	63.12	97.41	65.35	75.54	95.21	67.89	111.61	150.86	115.70	81.14	87.54	68.74	89.69	90.01
chr8	102504999	102505017	GRHL2	67.53	91.21	46.79	76.32	87.63	70.00	86.16	126.79	84.89	129.68	93.00	57.95	71.47	84.83
chr8	102555470	102555664	GRHL2	40.05	53.58	56.53	53.29	63.58	44.38	58.39	81.35	77.23	44.75	61.01	36.35	47.58	55.87
chr8	102564944	102565010	GRHL2	57.10	48.24	32.79	54.90	57.93	24.60	60.70	68.64	64.57	33.04	47.28	38.03	52.40	48.99
chr8	102570648	102571040	GRHL2	54.04	79.48	44.68	56.43	87.39	45.85	72.96	106.91	72.95	116.25	69.01	46.48	59.93	71.04
chr8	102582568	102582622	GRHL2	64.20	144.04	72.15	70.89	117.29	77.13	118.18	184.87	133.55	2.85	96.95	67.98	89.76	95.84
chr8	102585897	102586052	GRHL2	45.30	59.20	43.81	43.69	56.08	37.65	55.06	66.31	75.69	74.21	51.02	30.31	50.34	53.19
chr8	102589637	102589747	GRHL2	26.54	53.87	42.22	33.86	73.41	39.98	47.20	80.66	65.03	45.72	51.28	29.50	51.92	49.11
chr8	102611286	102611379	GRHL2	86.67	128.18	109.82	79.78	126.24	87.48	145.60	223.37	173.61	7.73	96.97	112.80	110.50	114.85
chr8	102631768	102631925	GRHL2	85.75	164.63	101.84	85.53	128.38	84.04	176.99	233.89	186.11	4.22	131.07	95.27	113.53	123.14
chr8	102643866	102643952	GRHL2	65.43	73.66	47.86	56.31	90.03	32.06	56.47	104.11	90.91	76.67	71.00	49.54	74.62	67.84
chr8	102644455	102644593	GRHL2	57.05	83.35	51.91	47.59	83.19	65.68	98.54	157.91	104.24	33.35	76.75	61.40	68.63	76.75
chr8	102649126	102649156	GRHL2	100.45	157.55	90.65	107.00	148.68	95.10	161.19	244.39	173.10	39.45	109.52	89.32	98.74	126.37
chr8	102656360	102656453	GRHL2	71.95	82.93	44.63	57.39	85.17	47.93	84.89	151.54	98.53	90.84	58.05	45.23	74.89	76.59
chr8	102661643	102661727	GRHL2	33.99	43.33	40.76	41.53	55.39	36.49	31.93	88.21	59.91	45.98	39.62	28.27	39.22	45.45
chr8	102676683	102676746	GRHL2	40.55	39.09	29.03	43.88	67.38	27.05	51.75	68.08	62.06	19.94	53.66	29.69	43.47	44.35
chr8	102678818	102678931	GRHL2	47.43	46.80	24.55	47.33	60.30	37.53	46.30	65.84	54.92	96.81	54.08	34.94	47.47	51.40
chr9	75192819	75192939	TMC1	0.58	0.00	0.02	0.00	0.00	0.14	0.00	0.80	0.79	0.00	1.31	0.43	1.42	0.34
chr9	75231285	75231393	TMC1	0.00	0.00	0.00	0.00	0.02	0.00	0.92	0.00	0.53	0.00	0.00	0.00	0.00	0.12
chr9	75242838	75242979	TMC1	19.98	33.85	24.10	33.95	30.05	26.65	35.29	40.07	27.87	34.79	29.45	12.07	25.15	29.01
chr9	75263514	75263580	TMC1	33.45	55.21	31.85	38.73	45.22	32.79	46.13	63.24	58.88	36.24	44.51	28.46	40.22	42.89
chr9	75303626	75303672	TMC1	51.74	86.57	60.04	51.28	98.43	42.96	81.96	87.26	105.11	7.47	83.74	66.64	67.91	68.60
chr9	75309460	75309630	TMC1	26.98	34.64	28.29	24.96	33.74	25.87	42.84	40.39	30.60	2.62	43.70	18.20	28.81	29.40
chr9	75315435	75315559	TMC1	23.59	30.03	18.49	29.50	24.02	27.46	33.74	53.30	35.03	0.00	26.42	20.75	14.66	26.86
chr9	75355036	75355125	TMC1	75.44	119.59	67.77	69.53	97.83	52.72	101.32	143.80	136.29	24.71	98.27	57.58	70.83	87.07
chr9	75357361	75357441	TMC1	51.01	78.49	50.25	42.11	60.22	37.09	82.33	137.06	79.20	27.78	64.85	41.99	38.80	62.70
chr9	75366767	75366872	TMC1	19.54	49.66	18.14	22.95	32.65	22.09	60.28	56.07	39.75	0.60	30.84	28.07	19.31	31.72
chr9	75369703	75369800	TMC1	65.80	116.42	69.16	73.66	93.01	65.44	112.63	166.22	132.81	109.38	93.11	70.03	80.83	97.31
chr9	75387330	75387471	TMC1	92.63	215.12	112.03	83.77	152.77	88.55	191.98	294.11	219.55	72.43	140.57	104.08	104.44	147.30
chr9	75403256	75403399	TMC1	54.40	101.71	62.23	73.84	98.33	46.18	114.78	144.18	124.97	42.96	77.89	59.64	56.92	83.43
chr9	75404040	75404233	TMC1	36.64	52.60	44.11	37.77	53.57	38.87	65.56	91.07	56.51	43.14	42.01	31.98	37.26	49.49
chr9	75406803	75406981	TMC1	25.73	69.85	35.47	34.89	49.97	40.98	65.13	116.69	75.07	8.13	39.96	38.68	45.12	50.05
chr9	75407108	75407268	TMC1	91.77	146.34	89.77	76.31	136.60	73.27	131.50	196.52	168.24	35.40	111.42	99.37	103.40	113.04
chr9	75420299	75420426	TMC1	120.11	207.83	153.70	105.83	152.87	148.65	202.38	311.52	235.86	22.68	147.00	121.13	135.34	160.80
chr9	75431060	75431126	TMC1	55.07	108.39	72.81	63.57	77.10	50.79	97.21	168.88	106.48	54.82	89.87	68.48	62.33	84.46
chr9	75435759	75435997	TMC1	41.90	77.07	57.66	50.71	70.15	53.44	84.99	137.76	90.36	53.49	53.21	43.68	49.51	67.87
chr9	75441786	75441910	TMC1	106.35	129.07	79.80	88.70	128.23	90.32	151.38	235.75	169.57	72.46	101.47	71.66	99.43	118.73
chr9	75445368	75445445	TMC1	18.15	29.83	25.06	17.17	23.08	15.13	28.06	25.78	38.31	4.71	27.44	16.38	22.47	22.43
chr9	75445548	75445598	TMC1	33.90	61.33	36.55	22.57	43.31	33.02	47.86	33.14	66.06	11.80	48.29	31.00	32.02	39.07
chr9	75450868	75450889	TMC1	85.77	97.91	69.41	76.73	118.59	62.91	108.45	120.59	118.64	69.55	101.18	86.27	74.00	93.00
chr9	117165035	117165216	WHRN	30.31	50.24	20.01	32.34	59.39	38.51	53.03	61.04	64.33	89.27	49.53	26.28	51.06	47.86
chr9	117165498	117165619	WHRN	69.10	114.95	65.57	66.52	119.24	58.93	110.22	138.36	107.58	159.39	54.35	55.98	87.16	93.35
chr9	117166177	117166357	WHRN	42.56	46.18	34.40	64.82	78.14	33.98	68.11	63.10	60.98	132.26	66.78	33.62	71.17	60.41

chr9	117168636	117169172	WHRN	48.42	51.81	34.14	54.70	81.35	37.56	52.24	78.76	56.50	126.26	50.27	36.77	53.93	59.07
chr9	117170228	117170298	WHRN	41.49	23.83	23.59	48.68	59.79	29.25	44.01	50.55	45.82	73.69	45.97	28.68	42.28	42.95
chr9	117185595	117185803	WHRN	34.64	49.19	38.10	49.31	77.60	46.41	54.45	95.39	54.65	102.72	55.88	34.35	51.20	57.72
chr9	117186615	117186826	WHRN	12.35	26.00	17.92	23.97	31.70	18.55	15.97	27.46	30.25	52.99	16.21	13.63	24.36	23.92
chr9	117187269	117187304	WHRN	45.17	76.72	47.72	58.69	84.75	54.17	73.94	103.50	68.25	83.39	66.78	34.28	55.78	66.45
chr9	117188492	117188693	WHRN	21.12	25.19	22.90	20.63	31.17	21.17	20.71	42.52	37.93	48.56	24.87	21.36	34.81	28.18
chr9	117228548	117228672	WHRN	96.90	106.83	71.84	89.37	155.50	62.91	102.30	121.42	139.87	228.25	106.46	62.60	97.31	112.02
chr9	117240834	117241051	WHRN	63.28	88.17	66.43	88.63	140.24	64.87	111.01	131.28	109.07	188.17	85.87	56.39	89.78	99.45
chr9	117266465	117267081	WHRN	66.95	80.37	57.86	78.51	146.96	65.19	82.39	104.21	99.99	181.61	73.84	59.06	87.89	91.41
chr9	140086565	140086626	TPRN	66.97	73.94	60.26	84.85	123.82	75.47	76.55	91.11	82.13	180.66	81.82	44.02	107.42	86.80
chr9	140086712	140086817	TPRN	61.36	56.68	50.19	64.16	110.63	55.22	67.74	85.78	60.07	128.95	64.77	51.33	86.42	71.41
chr9	140086904	140087143	TPRN	77.04	80.26	64.02	93.70	127.57	53.19	95.96	121.40	97.99	201.81	78.00	71.30	109.26	96.85
chr9	140093440	140095163	TPRN	39.42	60.32	37.70	43.76	72.27	42.26	54.67	76.42	57.07	102.74	44.41	37.65	51.55	55.73
chr10	26224674	26224759	MYO3A	0.00	0.00	0.90	0.00	0.00	1.95	0.42	0.95	0.00	0.66	0.00	0.00	0.00	0.41
chr10	26241024	26241207	MYO3A	28.93	76.76	30.94	27.77	41.31	28.99	60.41	89.45	60.51	4.38	37.55	30.80	33.23	43.15
chr10	26243804	26243937	MYO3A	20.23	55.62	42.70	29.67	45.89	35.21	54.88	100.31	57.79	0.11	23.86	26.59	33.03	41.07
chr10	26285420	26285523	MYO3A	44.18	58.53	36.27	30.42	43.79	36.01	40.38	87.65	51.42	0.43	45.67	23.05	27.64	41.48
chr10	26286089	26286187	MYO3A	29.73	59.44	32.36	33.67	45.46	26.70	58.83	92.79	64.31	0.00	23.22	31.83	24.78	41.53
chr10	26305750	26305825	MYO3A	54.96	95.79	68.07	68.25	103.13	54.87	102.45	146.80	109.91	62.01	80.97	59.63	75.82	83.90
chr10	26310433	26310577	MYO3A	77.36	99.85	58.99	64.39	102.50	62.14	119.69	163.48	140.63	64.73	102.66	57.44	86.48	92.82
chr10	26312952	26313016	MYO3A	129.31	226.28	133.00	123.55	169.75	133.38	220.58	301.48	233.68	10.06	190.11	126.68	157.80	166.49
chr10	26315307	26315461	MYO3A	54.50	80.97	55.25	41.16	70.32	52.87	94.24	99.87	102.90	12.59	56.28	46.79	59.90	63.98
chr10	26355905	26356003	MYO3A	69.43	94.16	57.91	68.49	90.85	60.35	93.54	134.21	140.55	2.00	44.01	72.03	58.98	77.29
chr10	26357698	26357813	MYO3A	84.87	155.69	96.15	75.63	114.73	76.28	158.48	186.31	169.23	1.00	97.58	75.59	74.93	107.63
chr10	26359041	26359144	MYO3A	18.02	50.60	18.46	21.45	24.48	28.49	41.35	53.75	51.51	2.00	27.21	22.08	28.97	29.95
chr10	26359248	26359330	MYO3A	22.66	53.07	34.88	29.17	30.18	42.10	46.66	58.99	58.14	2.11	37.42	35.45	26.82	37.57
chr10	26377133	26377334	MYO3A	28.48	55.17	44.79	28.68	51.79	38.42	50.48	61.66	66.04	1.60	34.32	33.54	31.48	41.25
chr10	26385311	26385408	MYO3A	28.36	40.36	34.21	21.31	34.13	27.23	42.84	42.13	57.02	1.81	30.26	32.12	26.71	32.65
chr10	26385498	26385611	MYO3A	30.39	48.33	29.93	21.69	38.54	32.82	65.17	64.19	74.24	0.26	35.61	36.60	28.77	39.82
chr10	26409606	26409731	MYO3A	43.58	67.53	48.48	39.90	83.68	49.70	96.07	123.16	93.92	20.75	61.04	52.93	51.50	65.06
chr10	26414328	26414537	MYO3A	29.78	54.91	31.46	48.49	53.49	35.11	54.17	73.89	59.81	24.48	50.74	37.57	34.30	46.16
chr10	26417321	26417467	MYO3A	16.00	34.73	21.84	23.29	28.42	19.56	31.88	48.99	39.79	1.78	18.86	22.99	19.21	25.68
chr10	26432378	26432530	MYO3A	82.03	130.96	91.75	71.08	114.04	74.61	141.42	182.47	145.42	8.02	77.95	85.33	91.95	100.42
chr10	26434376	26434463	MYO3A	30.09	63.45	39.70	31.99	48.13	40.82	74.26	93.55	78.53	2.80	39.24	38.18	34.02	48.39
chr10	26436360	26436488	MYO3A	135.68	204.86	129.13	119.40	195.29	115.09	237.67	377.53	248.36	21.72	136.71	124.74	149.18	170.52
chr10	26442780	26442858	MYO3A	32.10	56.08	38.73	19.73	29.85	33.99	49.00	70.30	65.67	1.25	34.41	43.05	19.66	39.51
chr10	26443676	26443752	MYO3A	62.03	74.64	42.23	55.94	86.26	46.18	75.51	92.97	87.62	8.17	63.69	39.45	57.78	61.22
chr10	26446240	26446444	MYO3A	94.49	150.73	112.31	92.27	128.08	82.73	164.76	199.90	163.54	57.23	135.74	84.35	103.55	122.18
chr10	26454997	26455107	MYO3A	25.80	23.25	18.35	27.11	33.03	18.18	31.16	37.77	34.14	26.30	23.17	15.20	20.95	26.12
chr10	26457642	26457803	MYO3A	9.77	32.40	17.67	12.12	18.09	10.30	32.20	31.59	33.42	2.03	20.54	16.41	13.46	19.71
chr10	26459346	26459468	MYO3A	20.07	36.33	25.47	15.02	18.74	15.93	25.11	50.93	42.38	1.38	18.30	14.24	19.53	23.66
chr10	26462593	26463486	MYO3A	76.67	113.44	82.52	82.94	110.63	58.66	109.48	144.57	130.78	34.05	92.61	73.00	91.52	92.45
chr10	26465631	26465774	MYO3A	76.27	133.95	89.38	83.94	122.19	95.57	159.81	229.86	160.92	12.19	111.42	87.28	88.76	113.57
chr10	26482135	26482240	MYO3A	24.92	64.57	41.24	36.61	36.92	32.08	43.79	65.00	67.07	16.33	39.83	47.92	40.18	43.02
chr10	26490195	26490234	MYO3A	15.55	36.88	30.15	17.18	16.48	16.33	22.13	28.68	26.83	4.05	25.45	15.10	14.88	21.23
chr10	26491894	26492036	MYO3A	62.25	128.82	84.24	67.06	104.48	77.03	101.98	129.91	131.55	0.85	89.44	67.62	82.43	87.10
chr10	26500773	26500892	MYO3A	45.12	74.44	49.54	58.20	95.17	41.64	49.86	76.12	61.07	121.36	57.34	41.82	61.47	64.31
chr10	55566340	55566890	PCDH15	68.60	113.25	82.89	59.81	110.81	76.69	117.26	167.98	146.18	68.57	96.25	78.09	84.53	98.87
chr10	55568453	55569306	PCDH15	76.80	124.42	88.61	80.94	113.86	83.92	155.63	212.92	162.24	46.33	104.46	92.66	92.06	111.90
chr10	55570317	55570426	PCDH15	79.86	114.26	78.55	90.70	102.51	77.45	110.13	137.93	144.72	23.15	109.40	85.50	91.75	96.18
chr10	55571310	55571381	PCDH15	18.97	39.67	30.50	17.00	26.15	20.60	31.22	39.15	30.65	0.00	18.71	21.47	16.13	24.51
chr10	55581619	55583118	PCDH15	70.22	114.92	71.86	66.48	88.04	62.95	121.81	175.03	132.51	33.44	83.87	63.82	72.14	90.41
chr10	55583190	55583193	PCDH15	14.00	30.25	30.00	22.50	26.25	20.50	31.00	41.75	28.00	1.00	12.00	17.25	14.25	22.88
chr10	55587150	55587308	PCDH15	44.57	111.58	62.33	56.05	94.06	69.82	99.58	130.60	116.74	96.71	60.98	69.21	63.38	84.35
chr10	55588326	55588333	PCDH15	25.13	55.38	39.13	16.38	32.50	35.88	68.88	72.50	57.25	0.00	44.88	53.13	27.00	41.75
chr10	55591076	55591293	PCDH15	40.58	40.27	61.16	49.98	45.83	23.11	75.49	96.19	58.42	54.49	40.27	38.44	41.32	52.02
chr10	55600081	55600256	PCDH15	70.45	118.76	84.97	86.97	110.61	68.12	132.60	181.92	146.05	59.38	88.84	68.10	94.34	101.40
chr10	55616936	55617023	PCDH15	28.36	71.26	47.30	34.82	37.42	35.76	65.85	72.75	87.63	3.16	44.51	35.93	39.41	47.06
chr10	55626403	55626617	PCDH15	62.92	111.67	78.37	63.78	92.01	58.23	123.16	152.93	146.23	1.22	69.64	72.19	64.54	86.03
chr10	55663004	55663130	PCDH15	10.97	40.58	27.24	20.40	23.06	28.80	47.73	73.82	50.63	7.69	25.11	35.86	33.90	32.66
chr10	55698576	55698715	PCDH15	62.64	113.06	67.33	54.95	99.98	53.79	94.90	134.91	129.82	1.00	71.01	68.22	80.87	79.30
chr10	55700627	55700735	PCDH15	97.22	139.74	101.10	84.06	95.87	96.61	146.37	165.29	160.50	9.87	100.66	86.57	79.49	106.99
chr10	55719493	55719604	PCDH15	81.99	124.65	100.58	89.07	115.63	89.41	158.21	179.30	166.04	41.89	122.88	106.22	93.39	114.66
chr10	55721513	55721652	PCDH15	48.22	109.90	84.85	35.41	59.94	61.88	110.95	139.86	124.79	0.00	81.24	78.86	72.29	77.99
chr10	55755410	55755525	PCDH15	71.40	143.13	98.61	93.58	109.34	72.87	122.71	245.39	159.25	4.97	103.10	78.99	78.33	108.61
chr10	55779953	55780176	PCDH15	95.54	140.33	83.45	86.49	123.78	99.54	146.58	225.94	147.30	33.83	116.54	112.31	96.84	117.63
chr10	55782653	55782957	PCDH15	79.44	134.48	92.49	78.61	120.96	87.14	143.29	206.15	160.42	26.04	103.45	83.95	93.69	109.70
chr10	55826518	55826645	PCDH15	108.88	164.48	104.78	92.66	121.00	117.23	174.98	214.47	179.21	6.10	119.77	94.66	103.72	124.85
chr10	55839092	55839184	PCDH15	97.19	186.42	105.48	92.47	123.72	90.01	159.53	237.70	186.82	10.89	151.15	103.13	102.22	128.71
chr10	55849745	55849823	PCDH15	38.95	72.96	41.49	31.08	52.87	42.32	69.47	83.20	84.94	2.51	64.61	40.77	42.78	52.10
chr10	55892636	55892767	PCDH15	36.37	46.71	29.07	33.01	53.39	25.04	54.53	67.79	55.92	14.89	38.90	35.13	31.16	40.90
chr10	55912861	55913053	PCDH15	44.80	73.80	50.29	41.77	64.82	48.20	73.04	114.96	71.78	76.88	53.61	44.07	39.58	63.17
chr10	55943205	55943353	PCDH15	96.46	163.50	105.36	64.95	120.92	85.59	175.05	281.53	212.07	6.09	104.52	92.92	94.16	125.75
chr10	55944895	55945028	PCDH15	88.66	162.75	93.34	96.91	136.42	94.99	167.93	236.96	189.49	28.69	151.22	101.22	99.10	129.05
chr10	55949125	55949144	PCDH15	19.25	32.05	25.30	16.40	18.20	24.35	39.35	45.65	46.65	2.95	30.00	35.55	35.45	27.98
chr10	55955444	55955649	PCDH15	87.28	127.76	85.49	93.66	120.32	74.44	131.75	164.83	170.90	3.16	107.77	78.17	92.61	103.79
chr10	55973697	55973808	PCDH15	54.64	93.13	47.05	47.99	75.33	61.59	89.09	131.68	84.21	2.67	72.63	53.01	47.37	67.75
chr10	55996584	55996691	PCDH15	83.35	140.05	88.96	75.57	132.69	85.83	121.48	171.14	169.61	6.91	104.93	87.06	89.85	105.63
chr10	56077032	56077201	PCDH15	71.72	119.14	61.81	76.91	116.61	54.21	104.22	142.85	127.98	83.88	87.27	63.36	74.45	92.50
chr10	56089357	56089466	PCDH15	29.97	60.04	38.16	20.75	39.15	25.46	61.75	87.76	73.42	0.00	30.39	33.95	29.60	41.73

chr10	56106126	56106244	PCDH15	64.74	140.81	89.88	65.13	84.07	84.04	148.67	160.50	140.88	3.45	72.60	77.65	65.29	94.37
chr10	56128881	56129035	PCDH15	49.01	81.41	62.15	56.38	78.70	58.68	96.06	101.90	111.37	16.42	58.55	44.76	63.16	67.95
chr10	56138543	56138702	PCDH15	45.67	74.59	41.68	34.74	73.54	45.16	55.07	91.70	89.01	17.22	61.72	52.73	47.51	56.90
chr10	56287573	56287637	PCDH15	46.55	82.43	49.28	43.80	67.86	37.86	76.74	81.72	100.78	0.00	43.52	35.43	35.98	55.50
chr10	56288149	56288162	PCDH15	39.43	75.57	40.50	34.71	37.64	33.71	64.71	77.21	90.50	1.00	42.36	41.79	39.86	48.26
chr10	56423933	56424050	PCDH15	80.38	122.19	77.74	89.16	114.61	53.67	121.36	171.15	141.32	4.63	85.94	73.47	75.69	94.64
chr10	73199585	73199655	CDH23	30.04	45.87	31.73	34.45	55.31	47.59	51.86	74.94	53.99	105.38	33.04	24.49	46.97	49.06
chr10	73206076	73206152	CDH23	32.34	40.62	31.47	37.99	58.00	26.62	48.38	71.49	47.40	73.69	51.48	38.09	35.32	46.46
chr10	73269840	73269981	CDH23	57.37	94.72	60.79	82.08	120.52	63.28	94.25	140.12	102.51	193.27	80.82	58.13	83.76	95.65
chr10	73270712	73270758	CDH23	59.74	72.66	49.83	54.04	100.55	51.66	67.94	99.64	91.53	161.77	64.04	49.60	85.62	76.92
chr10	73270878	73270969	CDH23	96.05	106.59	81.25	106.38	168.93	75.89	121.32	166.12	128.76	296.60	110.23	71.65	116.76	127.48
chr10	73326500	73326693	CDH23	59.66	85.62	55.64	77.19	93.63	65.45	83.95	121.48	102.52	162.21	83.21	55.44	66.42	87.17
chr10	73330548	73330675	CDH23	47.76	86.30	48.24	50.40	87.96	73.06	79.20	125.18	102.77	77.25	58.59	48.20	70.92	73.74
chr10	73337672	73337749	CDH23	73.56	95.42	62.14	78.55	97.31	74.19	77.44	101.78	86.50	157.13	91.26	45.41	56.26	86.72
chr10	73375262	73375373	CDH23	85.30	94.54	66.54	89.03	144.71	69.17	90.00	137.65	88.89	197.70	101.19	66.68	108.58	102.62
chr10	73376963	73377237	CDH23	39.03	48.54	36.01	42.03	54.41	35.41	53.70	78.04	55.17	85.13	55.48	32.25	50.63	51.27
chr10	73405583	73405737	CDH23	55.75	67.86	47.76	65.62	93.62	54.33	58.59	86.47	73.90	141.12	60.49	46.07	59.45	70.97
chr10	73406217	73406518	CDH23	30.04	47.07	26.39	38.78	46.40	33.43	41.27	43.80	46.75	82.70	37.80	35.94	34.55	42.53
chr10	73434870	73434933	CDH23	38.28	32.56	23.73	35.25	40.84	30.50	40.14	79.75	48.64	68.61	55.22	23.55	38.77	43.09
chr10	73437214	73437450	CDH23	81.96	88.76	66.17	76.73	148.64	61.47	95.76	156.73	123.86	227.59	84.44	66.43	93.03	106.55
chr10	73439145	73439249	CDH23	108.33	97.79	83.67	91.41	159.64	101.52	109.90	195.78	117.66	236.10	121.74	78.31	147.68	125.15
chr10	73442203	73442329	CDH23	52.19	49.32	27.54	40.72	58.06	42.05	48.71	70.27	57.39	88.19	54.56	35.72	52.11	52.06
chr10	73447405	73447476	CDH23	53.58	50.75	59.22	85.24	126.85	38.29	72.65	80.81	73.36	160.08	69.42	47.17	73.50	76.45
chr10	73450226	73450341	CDH23	30.96	31.15	22.42	27.45	41.35	29.41	26.22	34.00	29.84	55.76	26.53	15.46	30.59	30.88
chr10	73453905	73454016	CDH23	45.08	38.32	19.94	42.87	78.36	20.49	40.04	52.65	41.88	106.79	46.02	23.26	45.16	46.31
chr10	73455176	73455282	CDH23	116.99	133.15	104.27	141.63	209.66	107.69	139.77	191.22	159.79	327.47	146.34	96.71	156.92	156.22
chr10	73461780	73461968	CDH23	74.65	92.36	55.18	100.85	143.69	77.24	107.61	126.97	101.55	203.90	99.93	76.09	96.53	105.00
chr10	73462307	73462451	CDH23	70.74	114.03	70.93	105.92	110.74	84.28	123.94	163.95	111.46	233.23	105.14	80.23	102.14	114.55
chr10	73464669	73464887	CDH23	55.86	62.60	40.79	65.59	97.44	42.58	63.58	86.98	84.44	125.76	43.90	39.59	52.91	67.43
chr10	73466655	73466886	CDH23	52.07	60.19	42.71	68.76	107.40	50.02	53.21	86.83	62.06	144.88	74.40	36.51	79.81	69.92
chr10	73468856	73468968	CDH23	33.68	35.38	31.99	39.74	72.58	27.96	39.81	52.04	55.55	83.35	44.63	25.39	42.02	45.17
chr10	73472423	73472570	CDH23	89.31	109.22	72.32	118.69	161.75	76.20	125.14	146.08	124.08	222.07	101.20	74.28	108.85	118.36
chr10	73483803	73483862	CDH23	63.50	101.17	69.62	87.77	127.13	61.30	102.43	117.65	88.50	188.20	84.73	37.75	77.40	94.15
chr10	73485130	73485277	CDH23	52.49	68.59	60.24	60.88	93.44	55.84	93.95	115.57	94.28	141.55	63.74	61.41	62.81	80.16
chr10	73490227	73490361	CDH23	24.62	26.23	18.53	43.72	47.39	16.07	27.62	39.48	28.87	63.69	37.45	10.97	39.42	32.05
chr10	73491745	73492174	CDH23	110.46	120.51	91.17	127.85	188.73	99.75	135.01	210.88	151.83	269.03	115.92	95.41	123.06	143.05
chr10	73493998	73494098	CDH23	38.04	42.51	40.53	60.69	73.97	45.12	54.39	83.02	73.16	120.77	57.46	42.17	58.16	60.99
chr10	73498253	73498404	CDH23	54.28	74.65	47.69	63.72	79.93	64.01	83.19	112.07	81.65	137.57	68.32	52.58	63.79	76.64
chr10	73499402	73499529	CDH23	45.84	32.89	22.77	42.90	72.69	20.34	31.09	51.55	42.30	91.06	43.63	27.41	46.59	43.70
chr10	73500580	73500707	CDH23	95.61	124.80	90.43	145.55	183.55	111.59	132.16	175.21	143.74	284.27	124.43	97.34	132.90	142.39
chr10	73501452	73501678	CDH23	64.63	66.39	67.24	82.20	127.82	66.58	84.47	133.08	100.38	176.80	76.88	70.32	85.30	93.07
chr10	73537438	73537658	CDH23	39.37	53.57	42.84	54.43	73.80	41.06	63.90	86.25	55.95	121.74	49.81	34.73	45.98	59.79
chr10	73537947	73538065	CDH23	51.29	48.66	38.66	52.95	71.72	33.75	37.64	72.34	50.58	103.24	46.22	27.37	59.41	52.87
chr10	73539025	73539204	CDH23	73.76	107.48	73.52	75.02	117.11	78.86	115.42	157.12	143.63	158.28	82.31	68.86	76.27	104.28
chr10	73544045	73544177	CDH23	49.74	77.05	46.77	80.29	91.46	49.20	81.96	97.89	68.67	138.30	76.11	43.98	57.98	75.12
chr10	73544649	73544857	CDH23	58.48	77.47	49.71	62.54	90.26	69.08	78.38	122.15	83.49	143.32	68.33	49.78	55.58	79.42
chr10	73545389	73545495	CDH23	79.06	102.27	76.67	97.48	131.15	66.46	88.33	131.84	101.63	185.87	86.23	78.11	96.03	102.09
chr10	73548698	73548799	CDH23	93.79	114.24	82.50	92.14	164.03	74.16	121.69	176.53	166.05	223.70	110.83	70.14	106.49	124.15
chr10	73550046	73550170	CDH23	89.22	102.87	66.09	91.26	160.71	77.32	112.84	167.85	122.48	236.09	102.40	68.65	109.94	116.48
chr10	73550890	73551092	CDH23	40.71	42.66	39.39	53.20	79.80	35.38	40.87	57.65	40.01	107.01	53.31	27.23	60.85	51.44
chr10	73552940	73553397	CDH23	97.28	124.41	92.94	109.48	162.68	102.18	129.41	181.06	152.56	225.83	126.37	86.66	128.87	132.57
chr10	73556862	73556977	CDH23	30.71	39.78	28.59	44.37	40.48	19.42	39.42	65.63	33.72	65.82	34.40	18.12	33.78	38.37
chr10	73558112	73558335	CDH23	20.89	26.03	62.05	33.04	27.19	77.60	40.23	48.96	41.61	29.59	26.34	26.29	26.34	38.32
chr10	73558869	73559037	CDH23	86.20	124.05	72.13	107.50	141.27	84.07	125.92	181.83	121.01	187.82	95.98	82.88	89.60	117.56
chr10	73559250	73559386	CDH23	35.55	42.21	31.63	35.66	50.82	25.19	40.19	66.99	51.63	82.10	50.24	28.70	46.45	45.08
chr10	73560394	73560512	CDH23	37.69	55.73	31.03	30.97	55.63	32.87	64.14	65.52	59.70	64.46	40.13	30.81	33.37	47.39
chr10	73562656	73562832	CDH23	112.91	109.94	78.33	146.92	190.28	104.93	129.56	194.94	135.60	323.24	128.29	98.66	153.66	146.13
chr10	73562967	73563177	CDH23	47.85	74.91	46.82	60.36	90.60	52.19	56.42	117.77	79.68	149.69	59.70	49.51	64.63	73.79
chr10	73565564	73565754	CDH23	59.16	83.53	53.55	81.40	113.88	62.96	73.72	134.48	80.03	158.58	73.50	49.59	73.94	85.37
chr10	73565926	73566038	CDH23	40.20	47.97	41.42	36.48	76.57	35.21	58.89	81.60	67.55	113.75	53.11	33.63	50.46	57.20
chr10	73567035	73567163	CDH23	44.71	52.68	43.12	66.67	98.13	51.55	52.27	70.61	62.40	121.19	59.11	23.54	62.66	62.16
chr10	73567274	73567524	CDH23	81.45	105.56	65.24	95.15	142.31	86.68	101.56	153.45	106.56	223.18	89.97	76.43	111.78	110.63
chr10	73567604	73567764	CDH23	86.91	98.22	56.62	80.08	140.35	56.76	101.99	136.94	106.48	202.72	84.66	49.09	111.53	100.07
chr10	73569578	73569833	CDH23	110.57	138.96	80.55	120.77	205.26	94.11	136.55	184.86	124.52	321.88	128.50	90.31	137.95	144.74
chr10	73570230	73570326	CDH23	64.63	76.76	54.77	73.75	119.44	64.15	88.18	127.92	68.45	175.59	102.34	59.70	80.94	89.64
chr10	73571073	73571192	CDH23	81.94	83.26	86.93	98.89	109.21	74.70	98.73	139.28	110.48	195.60	80.58	73.67	100.42	102.77
chr10	73571269	73571347	CDH23	89.95	107.56	88.89	107.92	150.68	79.68	119.58	161.34	136.13	255.25	104.37	77.14	107.28	123.21
chr10	73571471	73571510	CDH23	30.43	33.55	36.63	34.43	51.18	21.10	37.93	52.48	51.35	103.83	40.68	24.25	32.48	43.15
chr10	73571713	73571772	CDH23	63.23	108.78	82.40	71.25	127.50	78.13	100.62	146.10	110.40	174.35	92.22	80.60	92.83	102.97
chr10	73572050	73572119	CDH23	70.16	95.80	64.30	96.94	162.06	78.84	94.64	134.34	108.53	218.47	88.81	88.21	90.67	108.43
chr10	73572238	73572366	CDH23	119.91	145.53	89.12	122.01	225.61	99.23	147.97	190.79	158.53	313.26	108.72	99.81	142.45	151.71
chr10	73572526	73572647	CDH23	66.92	84.08	60.47	80.39	108.11	67.20	92.82	123.41	105.65	192.02	83.79	63.98	99.25	94.07
chr10	73573002	73573105	CDH23	38.84	55.68	36.38	51.40	86.61	48.49	57.47	89.46	73.10	120.47	58.64	49.01	53.82	63.80
chr10	73574710	73575035	CDH23	71.34	119.29	80.40	94.17	145.33	89.44	108.74	119.72	115.44	241.87	104.40	78.06	111.45	114.02
chr11	17515880	17515923	USH1C	22.27	39.41	9.14	19.16	40.48	24.52	22.11	43.20	31.07	51.00	21.45	14.07	31.30	28.16
chr11	17517117	17517224	USH1C	32.06	41.17	29.04	38.88	59.85	21.76	26.17	31.19	37.02	91.98	45.14	20.96	46.72	39.60
chr11	17518306	17518360	USH1C	51.73	98.47	58.95	75.78	103.20	51.76	76.78	124.29	87.64	162.09	65.20	47.25	78.05	83.60
chr11	17519710	17519818	USH1C	139.06	184.72	96.79	160.74	246.85	114.23	182.87	307.09	172.48	315.36	169.31	88.48	176.50	181.50
chr11	17522599	17522697	USH1C	37.72	62.29	43.70	41.63	82.89	53.72	60.30	90.33	69.88	125.30	44.81	49.55	67.17	63.51
chr11	17523030	17523082	USH1C	56.49	56.26	49.89	64.11	116.74	45.32	76.77	104.26	70.15	146.00	78.79	53.66	86.96	76.54

chr11	17523487	17523527	USH1C	63.95	72.39	42.02	47.76	82.90	58.10	57.59	96.76	71.10	105.02	68.80	32.15	55.29	66.54
chr11	17526195	17526244	USH1C	67.56	118.92	92.44	76.34	123.10	85.98	115.86	150.72	150.94	167.04	93.64	59.72	91.32	108.52
chr11	17527378	17527496	USH1C	46.92	51.46	25.08	37.10	63.35	38.52	53.34	52.93	60.55	104.07	52.18	25.77	44.73	50.94
chr11	17530904	17531385	USH1C	42.06	58.81	36.53	46.31	71.61	43.68	65.60	86.58	65.56	92.27	43.04	42.25	53.42	57.86
chr11	17531953	17532068	USH1C	20.11	30.87	10.48	17.04	30.09	14.09	25.10	31.45	25.44	31.72	20.95	14.03	19.81	22.61
chr11	17533450	17533601	USH1C	63.80	104.44	72.13	60.70	97.50	71.55	130.26	137.26	121.51	40.16	89.95	81.67	84.11	89.24
chr11	17537789	17537837	USH1C	39.37	35.12	20.55	29.53	43.08	20.84	41.69	63.73	55.04	53.88	38.92	25.61	34.29	38.95
chr11	17538949	17539021	USH1C	28.05	28.40	16.63	27.08	37.64	22.86	24.88	23.77	32.18	68.19	26.96	22.22	34.96	29.91
chr11	17542418	17542541	USH1C	53.07	86.85	52.07	55.23	89.27	66.44	92.44	142.34	107.83	77.71	72.62	54.63	63.77	79.21
chr11	17542894	17542958	USH1C	64.28	126.23	69.45	68.72	106.54	65.68	125.98	187.35	131.89	88.66	69.94	57.29	89.97	96.83
chr11	17544332	17544473	USH1C	43.90	40.08	22.22	29.25	57.06	29.32	39.05	30.26	37.35	76.76	36.94	28.33	45.58	39.21
chr11	17544759	17544814	USH1C	74.30	57.18	53.07	64.21	92.82	38.39	56.75	94.52	80.57	130.20	49.13	46.75	61.55	69.82
chr11	17544967	17545025	USH1C	37.64	30.76	56.24	35.54	31.61	72.56	43.68	58.54	41.39	35.54	39.08	32.05	36.24	42.89
chr11	17545999	17546082	USH1C	34.98	25.39	19.54	36.27	61.70	26.17	33.50	45.19	29.21	65.05	29.88	23.05	33.87	35.83
chr11	17547895	17547988	USH1C	44.82	91.60	73.54	89.00	109.98	54.83	82.67	106.11	82.27	173.13	70.65	48.82	90.68	85.62
chr11	17548301	17548357	USH1C	64.54	55.84	55.79	62.44	90.16	58.91	72.88	74.32	61.91	136.32	69.39	45.61	80.12	70.68
chr11	17548564	17548587	USH1C	20.71	15.00	20.46	19.92	38.38	33.88	24.54	24.83	24.79	60.79	22.29	12.58	32.21	26.51
chr11	17548771	17548878	USH1C	20.89	25.18	17.37	21.78	41.30	20.50	35.97	44.38	33.58	34.45	27.62	16.25	27.45	28.27
chr11	17552702	17552839	USH1C	28.36	29.20	28.58	42.86	64.04	26.26	41.33	39.91	43.06	84.78	34.52	19.61	47.57	40.21
chr11	17552947	17553089	USH1C	35.60	41.74	22.29	38.81	63.67	33.00	48.51	49.20	35.48	103.45	34.36	22.45	47.07	44.05
chr11	17554803	17554869	USH1C	24.00	43.34	30.36	37.33	41.72	41.31	49.24	46.78	34.39	65.61	35.58	27.93	35.19	39.80
chr11	17565820	17565963	USH1C	31.69	41.28	30.72	57.58	82.16	32.38	43.98	53.30	46.35	107.84	49.54	35.60	59.48	51.03
chr11	17568921	17569093	OTOG	27.66	38.42	25.24	27.22	33.56	20.39	39.23	44.18	27.81	57.78	32.76	19.49	25.66	32.81
chr11	17569475	17569534	OTOG	3.38	1.77	3.22	2.37	10.23	3.72	5.38	4.30	5.73	16.35	3.00	3.23	4.03	5.22
chr11	17569683	17569759	OTOG	33.52	21.03	23.56	40.17	65.99	18.87	38.29	47.74	37.96	72.81	40.92	21.65	42.22	38.54
chr11	17573548	17573622	OTOG	58.95	55.81	32.81	61.55	96.84	59.51	60.72	107.75	70.59	133.57	66.39	39.39	73.29	70.32
chr11	17574667	17574758	OTOG	79.77	101.30	62.63	86.84	120.01	52.63	101.77	159.78	103.84	203.20	84.09	56.40	97.39	101.02
chr11	17574913	17575066	OTOG	62.41	91.32	63.34	71.44	99.42	57.94	99.83	125.40	117.58	173.99	65.54	64.80	82.81	91.08
chr11	17577327	17577444	OTOG	28.42	42.76	24.93	37.08	53.58	23.74	36.81	58.15	34.75	66.75	41.42	26.28	30.83	39.56
chr11	17578666	17578870	OTOG	36.58	47.16	29.71	47.90	71.06	37.80	47.46	82.30	53.24	122.46	53.08	33.25	51.97	55.17
chr11	17579733	17579862	OTOG	38.20	39.15	30.55	43.64	55.89	36.35	40.02	52.20	51.98	94.21	47.38	34.84	53.85	47.03
chr11	17580086	17580191	OTOG	46.95	51.92	32.03	54.42	94.41	41.24	60.25	79.05	58.17	128.34	55.56	45.64	55.43	62.33
chr11	17580600	17580708	OTOG	38.83	48.46	39.08	42.50	76.70	30.79	62.61	96.79	67.89	106.55	51.06	32.86	49.84	57.84
chr11	17581082	17581209	OTOG	49.89	67.75	42.13	60.18	90.95	41.87	57.15	83.24	63.86	126.95	62.52	40.25	66.20	65.56
chr11	17582257	17582364	OTOG	24.69	72.06	47.66	34.95	58.13	43.41	61.27	87.51	54.61	78.89	46.62	38.66	47.01	54.04
chr11	17582639	17582684	OTOG	62.72	75.26	46.57	45.46	73.54	61.78	67.93	124.00	80.93	113.22	74.37	36.41	55.50	71.85
chr11	17583210	17583354	OTOG	53.58	57.67	47.86	56.52	76.88	42.26	65.12	94.95	79.76	144.34	62.29	46.11	65.66	68.94
chr11	17590704	17590835	OTOG	31.96	32.89	25.84	37.08	44.54	31.34	35.41	42.91	40.84	62.55	35.64	31.33	37.33	37.69
chr11	17591761	17591937	OTOG	49.04	52.95	31.62	44.78	92.85	32.19	62.54	75.31	66.02	125.73	51.31	42.85	54.56	60.60
chr11	17593628	17593751	OTOG	66.71	75.60	53.64	66.88	95.89	52.42	67.95	122.03	85.82	136.23	87.48	43.62	54.05	79.52
chr11	17594626	17594837	OTOG	36.96	56.25	30.75	45.90	66.82	42.13	52.38	74.16	58.78	97.42	46.06	29.67	55.36	53.11
chr11	17596268	17596459	OTOG	41.03	55.54	39.86	50.70	85.39	39.65	55.58	80.19	69.72	108.41	51.13	46.71	63.14	60.33
chr11	17598104	17598177	OTOG	60.61	58.58	45.84	69.50	105.58	58.15	74.77	127.73	90.04	146.97	88.34	53.32	73.35	81.62
chr11	17598416	17598458	OTOG	55.56	65.16	51.42	52.63	93.79	47.12	75.12	94.30	83.02	141.72	68.47	45.23	51.53	72.79
chr11	17599921	17600073	OTOG	34.72	42.42	30.62	45.31	78.42	35.67	40.41	66.58	46.48	90.42	42.91	23.87	44.61	48.15
chr11	17608022	17608128	OTOG	34.24	34.25	27.31	43.71	42.65	21.53	34.11	65.38	45.16	41.54	32.25	26.33	40.57	37.37
chr11	17612998	17613135	OTOG	56.99	70.91	48.65	57.92	94.20	46.28	56.09	102.27	75.35	154.50	62.52	52.57	76.05	73.19
chr11	17614741	17614874	OTOG	80.50	125.49	84.90	80.20	98.58	61.53	117.44	193.76	159.60	49.59	88.65	78.96	88.78	101.60
chr11	17615158	17615303	OTOG	13.78	14.60	6.02	18.78	22.86	15.45	16.51	29.42	20.29	39.59	22.40	8.31	16.80	19.00
chr11	17615595	17615713	OTOG	58.00	50.77	50.50	71.36	75.86	38.60	59.35	65.68	75.31	125.27	69.64	54.46	72.64	66.23
chr11	17617586	17617701	OTOG	60.70	66.44	43.88	57.97	82.84	44.43	55.56	103.16	61.07	103.57	64.46	45.93	49.45	65.83
chr11	17618399	17618554	OTOG	25.99	30.60	24.95	38.13	43.27	25.72	34.28	53.12	41.16	76.68	36.85	28.10	36.22	38.24
chr11	17621219	17621244	OTOG	53.85	88.85	60.35	65.50	92.23	61.38	89.35	110.00	101.92	137.65	73.27	62.08	73.58	83.04
chr11	17623758	17623924	OTOG	27.33	23.95	16.27	19.30	30.76	15.95	16.07	31.60	35.03	53.25	20.07	19.17	27.32	25.73
chr11	17627405	17627682	OTOG	52.99	55.08	43.61	61.91	84.64	49.37	60.31	75.48	65.32	137.91	58.02	40.94	75.24	65.47
chr11	17629844	17629960	OTOG	23.00	35.47	24.33	28.80	61.74	17.29	26.48	48.62	36.60	72.72	34.96	25.35	36.91	36.28
chr11	17630678	17630756	OTOG	47.28	54.54	44.65	50.27	70.47	32.33	60.99	81.01	55.59	69.61	37.66	41.01	47.06	53.78
chr11	17631203	17632970	OTOG	68.56	79.00	54.96	75.01	125.41	61.90	73.27	102.81	87.96	173.19	79.93	54.43	90.03	86.37
chr11	17633710	17633877	OTOG	37.50	42.83	34.42	39.81	63.72	39.03	49.83	69.32	42.86	100.45	45.55	33.62	38.51	49.91
chr11	17634168	17634312	OTOG	43.74	47.54	38.73	45.26	83.41	39.40	53.40	67.60	53.01	105.47	55.08	28.61	53.61	55.10
chr11	17635160	17635248	OTOG	50.44	52.99	40.25	56.19	84.25	42.96	62.99	111.00	64.62	122.04	60.80	37.02	59.44	65.46
chr11	17650681	17650863	OTOG	27.28	34.53	19.83	34.69	66.71	28.87	34.17	57.75	39.30	72.45	27.03	22.56	35.79	38.76
chr11	17653250	17653469	OTOG	57.83	72.01	57.75	57.18	101.88	52.82	86.71	117.02	76.83	140.34	71.93	49.85	79.59	78.51
chr11	17653636	17653773	OTOG	30.49	29.64	29.90	31.46	45.67	27.46	33.30	46.30	28.61	67.03	31.91	15.96	34.05	34.81
chr11	17655228	17655421	OTOG	17.44	21.96	19.98	27.21	53.12	24.81	26.74	29.93	32.76	79.04	28.12	19.25	30.99	31.69
chr11	17655617	17655828	OTOG	16.14	26.57	15.35	31.46	36.20	13.72	26.41	34.09	28.98	48.15	25.27	21.10	20.53	26.95
chr11	17656392	17656495	OTOG	14.13	25.26	18.05	14.62	28.82	19.70	28.17	31.46	38.19	41.84	12.97	22.18	17.29	24.62
chr11	17656628	17656734	OTOG	43.46	54.63	33.28	38.44	84.47	46.49	59.67	82.79	66.23	105.87	48.50	37.36	56.67	58.43
chr11	17657158	17657258	OTOG	63.60	70.47	51.17	69.31	113.88	65.35	75.78	110.59	71.39	167.59	74.75	48.65	90.95	81.88
chr11	17659999	17660096	OTOG	69.93	75.38	48.73	68.13	94.80	56.42	69.95	107.69	78.27	117.30	67.76	59.08	77.62	76.12
chr11	17660971	17661010	OTOG	64.63	101.00	60.45	62.15	109.63	76.88	91.13	140.30	92.55	129.65	82.95	64.18	92.03	89.62
chr11	17662293	17662367	OTOG	43.97	48.32	26.76	60.93	77.12	27.59	41.00	61.56	54.83	119.24	34.09	19.00	45.19	51.20
chr11	17662461	17662638	OTOG	33.90	47.81	31.77	48.59	64.92	36.19	50.87	65.48	54.73	99.36	39.59	22.58	51.99	49.65
chr11	17663395	17663498	OTOG	78.04	117.74	82.77	81.68	156.79	81.83	123.99	173.44	135.49	214.47	79.63	74.30	102.31	116.68
chr11	17663675	17663793	OTOG	30.44	48.93	29.08	31.43	74.93	29.40	35.26	67.87	53.78	103.84	45.07	25.89	39.54	47.99
chr11	17665009	17665053	OTOG	49.09	64.67	36.31	47.00	84.53	39.18	73.69	76.62	51.84	138.40	56.91	51.44	54.49	64.14
chr11	17667112	17667190	OTOG	89.53	100.24	66.49	90.86	149.14	78.48	119.28	150.51	132.32	240.30	92.53	68.22	98.57	114.82
chr11	17667292	17667491	OTOG	66.83	95.14	63.14	90.45	132.63	87.60	91.55	150.83	138.73	186.93	88.67	71.71	103.46	105.35
chr11	71799344	71799426	LRTOMT	67.63	113.67	57.94	94.27	97.18	59.18	101.82	160.17	97.00	42.67	59.00	55.05	85.95	83.80
chr11	71800076	71800211	LRTOMT	14.44	20.07	21.51	21.58	28.96	16.15	23.52	19.19	24.61	22.71	25.68	21.95	19.90	21.70

chr11	71800464	71800642	LRTOMT	44.17	72.48	40.30	41.79	60.76	30.85	65.67	70.86	76.05	29.33	52.72	35.91	46.61	51.74
chr11	71804543	71804747	LRTOMT	17.36	19.69	32.51	17.00	24.18	28.11	28.11	29.13	33.52	24.30	20.27	19.16	16.02	24.45
chr11	71805995	71806146	LRTOMT	70.57	94.97	66.38	66.18	107.41	55.24	97.01	105.80	95.11	126.86	96.93	57.74	64.14	86.68
chr11	71806426	71807938	LRTOMT	16.67	24.12	17.73	18.83	26.68	16.82	23.37	30.28	25.29	22.64	21.01	15.78	22.05	21.60
chr11	71816011	71816058	LRTOMT	47.90	60.21	54.31	54.85	72.79	51.96	49.88	82.48	65.29	128.54	57.25	47.31	66.63	64.40
chr11	71816983	71817256	LRTOMT	60.13	63.30	42.79	60.26	76.37	57.25	64.56	73.05	58.06	108.39	65.19	49.55	65.25	64.91
chr11	71818970	71819165	LRTOMT	73.62	124.53	69.08	91.91	119.43	81.82	121.62	141.05	127.39	175.72	98.01	75.67	100.26	108.32
chr11	71819652	71819971	LRTOMT	48.48	52.21	47.87	51.20	102.01	50.81	55.84	102.16	66.26	137.98	53.54	31.87	70.37	66.68
chr11	76841636	76841698	MYO7A	50.43	75.41	61.27	68.65	103.37	68.44	95.79	119.78	85.03	149.37	73.87	62.33	76.75	84.48
chr11	76853756	76853868	MYO7A	57.20	56.08	35.64	55.98	90.28	43.27	74.95	87.00	60.83	135.38	48.58	33.93	54.40	64.93
chr11	76858845	76858996	MYO7A	62.89	65.13	48.45	84.06	113.15	65.60	72.37	89.25	86.22	180.74	80.41	56.93	99.34	83.77
chr11	76866954	76867137	MYO7A	51.81	81.18	56.96	77.51	109.51	65.15	91.40	98.45	98.36	158.68	69.47	61.82	81.58	85.02
chr11	76867707	76867827	MYO7A	57.74	56.08	57.66	65.03	96.57	55.89	62.50	105.19	75.88	152.87	77.67	44.02	78.30	75.59
chr11	76867909	76868050	MYO7A	109.00	102.46	98.04	119.49	171.49	88.01	130.01	204.55	129.19	283.12	136.15	67.75	129.08	136.60
chr11	76868326	76868438	MYO7A	26.50	36.82	23.61	36.35	57.65	28.27	47.45	71.34	54.85	86.30	39.44	23.48	48.12	44.34
chr11	76869324	76869476	MYO7A	45.37	46.01	34.32	43.61	85.69	42.39	31.63	72.51	50.06	112.30	41.97	32.55	54.59	53.20
chr11	76870494	76870569	MYO7A	35.46	83.36	61.66	71.71	120.09	58.25	82.51	122.57	83.68	151.57	74.72	56.03	69.89	83.47
chr11	76871210	76871328	MYO7A	25.39	36.50	22.55	39.13	48.01	27.15	34.15	52.29	35.98	74.88	26.71	22.70	24.58	37.12
chr11	76872020	76872161	MYO7A	38.39	52.34	42.49	43.83	65.27	28.39	55.89	92.51	80.26	90.78	58.44	24.06	52.56	56.05
chr11	76873167	76873376	MYO7A	21.45	35.80	15.67	31.07	45.92	20.42	27.76	46.88	31.28	59.95	33.29	15.91	23.53	32.12
chr11	76873900	76874034	MYO7A	89.24	128.70	91.64	103.13	164.07	111.84	133.77	175.01	147.16	216.39	119.29	106.36	112.84	132.22
chr11	76877103	76877208	MYO7A	77.38	92.21	54.80	88.03	107.83	63.93	95.24	130.68	113.67	192.83	91.95	58.25	88.18	97.23
chr11	76883795	76883931	MYO7A	59.39	84.72	38.26	73.18	109.48	57.26	78.18	101.65	83.95	180.97	65.69	38.71	90.02	80.95
chr11	76885803	76885960	MYO7A	37.01	56.46	34.39	47.75	80.28	27.37	44.97	81.28	42.35	124.89	46.79	23.39	58.78	53.91
chr11	76886419	76886510	MYO7A	96.79	129.32	84.72	109.67	174.97	77.13	120.74	193.85	153.05	275.97	108.15	78.95	113.70	133.61
chr11	76888596	76888689	MYO7A	112.79	98.71	70.44	106.39	170.91	86.73	107.23	165.97	123.14	258.23	103.86	80.98	117.55	123.78
chr11	76890092	76890175	MYO7A	68.29	79.61	67.58	79.12	103.35	50.13	79.10	119.27	79.52	191.23	88.33	46.42	76.65	87.66
chr11	76890782	76890999	MYO7A	16.72	18.00	18.31	20.71	35.14	15.53	18.89	27.50	21.66	45.18	6.76	13.18	18.44	21.47
chr11	76891421	76891527	MYO7A	37.93	72.47	47.29	61.64	85.37	46.27	60.68	86.13	77.92	130.00	57.00	40.51	67.26	66.93
chr11	76892427	76892635	MYO7A	45.67	67.84	44.39	52.78	87.57	48.12	61.22	84.96	66.30	133.81	57.96	42.79	68.96	66.12
chr11	76892998	76893200	MYO7A	36.78	51.98	27.36	39.08	67.75	45.54	36.28	61.13	43.60	101.21	34.13	34.56	39.87	48.28
chr11	76893470	76893645	MYO7A	48.19	54.48	41.52	58.97	91.74	39.97	56.60	83.62	80.07	121.98	67.38	41.72	54.11	65.52
chr11	76894114	76894202	MYO7A	30.18	58.79	32.58	47.98	80.44	37.57	71.16	91.26	61.40	120.19	52.70	27.69	59.75	59.33
chr11	76895634	76895794	MYO7A	25.02	30.78	37.31	32.63	57.42	29.22	38.01	75.21	57.73	73.80	34.43	19.71	30.59	42.61
chr11	76900390	76900515	MYO7A	55.90	68.54	49.45	52.48	112.94	51.55	65.90	112.21	79.90	138.42	73.25	46.28	69.98	75.57
chr11	76901066	76901184	MYO7A	21.37	30.59	11.70	24.55	42.78	14.90	24.76	45.37	36.55	61.07	28.11	11.67	38.78	29.45
chr11	76901743	76901915	MYO7A	67.34	77.46	63.89	69.82	100.94	63.45	89.38	109.84	96.23	152.47	73.25	46.70	74.18	84.23
chr11	76903097	76903323	MYO7A	40.42	54.03	35.89	49.56	67.45	50.04	45.77	81.89	53.96	116.30	40.07	33.59	61.53	55.75
chr11	76905400	76905569	MYO7A	32.05	39.88	31.57	37.74	70.16	28.36	38.56	55.09	41.75	93.16	44.62	16.91	44.98	44.15
chr11	76908527	76908643	MYO7A	24.78	50.68	27.41	28.76	42.95	20.47	35.83	52.17	43.64	61.20	37.09	23.73	34.65	37.39
chr11	76909541	76909666	MYO7A	38.14	33.41	24.48	42.47	64.90	37.18	36.43	67.70	44.72	105.68	44.13	30.64	46.29	47.49
chr11	76910581	76910863	MYO7A	30.14	29.06	28.61	36.88	61.68	28.35	42.90	57.79	47.77	85.28	41.28	33.16	41.28	43.57
chr11	76912494	76912683	MYO7A	34.01	52.17	47.53	50.35	74.75	37.89	62.62	85.43	63.79	121.68	53.89	33.63	56.43	59.81
chr11	76913346	76913469	MYO7A	35.06	43.02	27.00	38.59	67.81	36.48	50.41	60.03	55.81	78.19	34.54	26.83	43.07	46.15
chr11	76914106	76914262	MYO7A	8.87	4.28	3.39	10.92	16.30	3.33	8.12	9.71	8.36	23.07	11.18	6.87	9.22	9.53
chr11	76915122	76915274	MYO7A	69.71	82.19	64.78	79.41	123.24	53.46	86.29	110.38	88.52	166.17	93.43	64.10	76.00	90.14
chr11	76916508	76916662	MYO7A	58.89	61.21	36.06	70.10	101.67	37.72	64.30	78.97	81.65	154.17	66.60	42.85	65.88	71.18
chr11	76917143	76917247	MYO7A	65.21	110.03	61.92	94.21	130.36	85.63	115.29	169.25	131.04	226.44	98.05	69.02	97.71	113.04
chr11	76918335	76918447	MYO7A	36.84	40.61	27.07	31.81	73.08	34.12	33.56	58.62	42.12	106.88	37.16	24.86	44.89	45.56
chr11	76919476	76919562	MYO7A	33.99	61.62	43.90	36.33	60.91	42.95	46.08	110.82	58.40	98.14	41.40	25.29	48.10	54.99
chr11	76919743	76919848	MYO7A	52.98	62.80	55.94	60.80	95.95	48.78	75.97	120.66	75.11	124.08	51.20	40.19	67.21	72.04
chr11	76922198	76922382	MYO7A	61.31	66.36	44.66	56.83	95.99	37.86	77.35	110.32	70.92	161.23	67.05	46.90	62.71	74.73
chr11	76922867	76922982	MYO7A	69.56	98.30	54.28	75.26	96.39	49.58	71.64	112.30	92.30	158.16	70.22	55.19	76.83	83.60
chr11	76923998	76924080	MYO7A	40.76	49.64	41.37	48.64	62.81	32.72	65.13	69.40	68.99	81.99	47.70	33.52	47.18	53.56
chr11	76924906	76925024	MYO7A	72.39	77.31	68.25	78.95	105.25	69.79	90.91	134.87	102.40	200.34	103.38	54.30	83.89	96.51
chr11	76925653	76925741	MYO7A	64.92	74.35	73.22	79.85	119.36	41.63	67.81	119.84	83.39	135.98	82.63	47.29	70.27	82.52
chr11	110070307	110070403	RDX	80.10	109.56	76.46	70.43	108.20	67.72	112.90	201.68	127.21	75.02	90.98	64.85	66.46	98.76
chr11	110102595	110102758	RDX	11.61	10.20	14.74	5.49	6.81	9.95	11.13	12.73	17.25	3.74	10.26	9.49	10.85	10.28
chr11	110103963	110104204	RDX	15.86	35.24	21.28	24.23	30.25	12.94	40.98	53.62	50.65	19.13	28.26	23.31	32.28	29.65
chr11	110106825	110106916	RDX	2.88	17.66	15.39	5.96	9.54	8.74	9.59	12.42	23.59	0.00	12.18	7.23	5.05	10.43
chr11	110108218	110108377	RDX	22.93	48.10	33.54	22.31	24.08	29.12	54.94	60.21	57.05	6.38	26.41	41.57	25.84	35.55
chr11	110118429	110118558	RDX	15.32	34.88	24.48	12.81	18.23	26.24	44.18	41.41	47.05	0.00	19.78	39.77	22.66	27.01
chr11	110124672	110124834	RDX	34.46	61.88	39.26	35.62	39.04	38.02	46.36	81.18	85.85	0.00	42.91	35.69	36.12	45.02
chr11	110126015	110126110	RDX	17.16	40.74	25.89	31.82	34.31	27.28	42.94	50.10	63.96	0.00	32.21	31.35	31.73	33.15
chr11	110128493	110128638	RDX	13.33	12.63	14.14	12.27	14.15	14.60	17.55	14.01	24.77	0.00	16.95	9.86	8.83	13.69
chr11	110128832	110128914	RDX	14.54	26.78	25.42	13.42	25.54	16.87	34.87	20.37	37.65	0.46	16.42	27.76	20.29	21.68
chr11	110134686	110134959	RDX	32.64	53.59	50.03	35.27	55.36	21.95	56.13	74.19	72.89	5.42	36.60	37.68	41.74	44.31
chr11	110135505	110135599	RDX	27.28	55.79	39.33	36.82	41.83	36.38	75.97	89.76	76.38	21.09	38.68	51.27	42.45	49.22
chr11	110143262	110143344	RDX	7.46	10.23	16.40	8.82	9.43	0.33	13.83	17.70	21.55	15.10	8.17	10.27	8.17	11.61
chr11	110150407	110150481	RDX	30.33	35.67	38.72	22.92	28.40	38.53	42.32	50.23	65.51	1.13	29.03	35.44	19.97	34.85
chr11	120973376	120973438	TECTA	56.51	86.51	57.81	45.87	73.89	51.63	77.30	133.44	99.19	3.68	61.76	51.59	55.94	66.60
chr11	120976541	120976673	TECTA	96.08	151.43	107.02	88.79	148.45	99.87	155.27	247.50	189.31	89.62	128.90	97.30	110.17	133.30
chr11	120979921	120980207	TECTA	56.10	73.52	50.03	62.65	72.59	44.16	81.13	122.51	102.85	83.25	82.86	48.60	49.32	73.35
chr11	120983782	120983918	TECTA	77.00	87.31	57.64	93.70	126.80	64.67	107.42	133.74	98.09	200.15	61.41	56.68	85.49	97.05
chr11	120984263	120984427	TECTA	38.41	47.11	32.88	34.52	53.45	24.76	49.13	60.47	74.33	73.25	46.10	33.61	34.92	47.33
chr11	120989016	120989427	TECTA	61.25	81.10	53.20	63.56	102.70	46.74	78.30	115.17	83.76	155.43	70.41	51.52	81.38	80.26
chr11	120996012	120996581	TECTA	77.84	105.61	76.88	99.38	158.38	81.53	110.01	165.19	129.75	237.22	95.68	73.15	112.89	117.55
chr11	120998462	120999053	TECTA	102.86	116.86	103.02	122.52	194.56	103.90	149.52	183.74	136.02	289.63	125.90	76.67	137.18	142.10
chr11	121000348	121000920	TECTA	98.39	112.54	87.72	102.38	133.62	90.30	125.46	174.45	139.17	203.18	94.89	73.09	118.37	119.60
chr11	121008131	121008731	TECTA	79.10	107.34	67.86	90.79	143.07	70.78	100.91	162.23	114.68	210.73	92.95	73.66	97.18	109.51

chr11	121016265	121016825	TECTA	78.53	102.79	68.54	86.49	134.69	73.48	116.16	164.80	117.00	182.36	89.92	71.83	91.24	107.21
chr11	121023591	121023789	TECTA	46.01	61.63	49.45	54.96	82.70	52.29	73.32	95.57	73.28	130.98	73.74	40.63	66.59	69.55
chr11	121028551	121028933	TECTA	89.35	111.94	83.23	107.11	154.25	89.21	121.25	156.20	142.59	220.66	101.10	81.24	109.61	121.51
chr11	121030845	121031130	TECTA	49.78	98.83	59.26	69.98	90.91	59.62	81.16	131.70	96.91	106.13	65.60	64.49	84.35	81.20
chr11	121032785	121033079	TECTA	51.10	40.25	32.16	55.64	82.02	44.66	48.66	71.85	64.53	106.47	44.33	43.50	58.33	57.10
chr11	121035983	121036092	TECTA	60.99	91.67	61.24	63.75	93.27	52.26	91.94	146.67	116.75	149.92	66.13	52.61	83.41	87.27
chr11	121037288	121037489	TECTA	23.91	52.88	28.96	34.00	67.28	31.99	60.55	71.76	50.50	71.57	42.92	28.68	39.88	47.08
chr11	121038764	121038926	TECTA	74.13	125.18	84.52	84.47	115.44	88.75	142.07	211.53	128.71	102.64	114.98	85.82	82.56	113.19
chr11	121039387	121039634	TECTA	65.64	103.78	72.94	77.35	93.44	65.53	105.90	138.69	126.72	86.25	99.24	63.28	80.54	91.56
chr11	121058542	121058703	TECTA	60.36	79.95	59.17	69.36	98.65	64.78	92.25	145.80	101.26	58.38	68.21	51.26	66.11	79.12
chr11	121059790	121059876	TECTA	97.78	164.15	98.10	91.02	134.28	86.56	151.64	215.61	170.18	52.98	117.75	89.59	118.64	122.47
chr11	121060474	121060589	TECTA	39.49	40.44	27.16	32.17	55.27	29.22	53.19	63.28	48.44	72.04	32.00	20.25	44.34	42.75
chr11	121061416	121061515	TECTA	91.67	148.63	80.11	82.67	124.73	87.82	154.37	211.79	151.49	14.92	93.58	93.19	96.85	111.25
chr12	15774252	15774364	EPS8	52.79	75.35	55.02	59.01	70.47	63.18	86.84	142.04	91.97	19.58	57.62	49.99	44.99	68.65
chr12	15776093	15776221	EPS8	124.22	228.98	147.55	124.53	181.81	131.18	244.81	312.72	264.56	55.65	159.74	146.42	149.85	176.85
chr12	15777162	15777341	EPS8	18.97	34.24	13.06	16.27	29.94	17.43	22.32	38.59	23.43	15.29	23.02	11.78	16.90	22.03
chr12	15784377	15784598	EPS8	27.05	44.21	31.50	33.83	56.56	35.74	45.99	76.95	42.17	60.15	34.21	34.66	32.95	43.59
chr12	15793638	15793780	EPS8	78.43	121.26	86.22	83.31	116.02	80.16	129.99	183.71	144.63	16.83	90.44	77.06	74.65	100.67
chr12	15794657	15794764	EPS8	15.05	44.85	35.29	8.17	26.40	29.40	36.52	23.52	55.81	1.43	20.61	25.06	20.65	26.84
chr12	15800062	15800194	EPS8	63.05	77.69	51.76	42.35	75.41	44.98	81.03	127.56	98.29	21.58	58.45	47.60	58.56	65.81
chr12	15803758	15803940	EPS8	80.46	132.87	80.40	85.49	122.15	65.95	145.50	181.81	149.10	56.13	107.96	63.98	85.66	105.98
chr12	15807080	15807227	EPS8	72.53	129.36	92.47	99.22	110.07	79.78	165.21	235.49	145.48	13.59	94.06	94.26	93.01	110.96
chr12	15811014	15811087	EPS8	65.61	111.23	76.35	66.77	105.54	53.69	126.26	158.89	125.23	4.66	82.08	58.72	64.80	86.25
chr12	15811432	15811519	EPS8	61.82	101.68	76.07	58.60	88.52	49.84	89.59	98.97	122.72	30.10	104.15	50.83	69.83	77.74
chr12	15813549	15813674	EPS8	19.49	24.52	31.94	22.60	27.87	25.92	32.65	39.87	42.08	17.28	28.41	25.84	27.28	28.21
chr12	15814961	15815033	EPS8	95.66	161.68	100.33	81.84	141.47	103.36	168.67	192.78	188.44	16.55	120.36	101.00	109.11	122.68
chr12	15818691	15818826	EPS8	42.72	48.46	38.91	43.86	71.24	34.85	60.15	74.10	70.01	60.08	42.42	42.18	37.94	52.42
chr12	15819375	15819456	EPS8	88.10	166.87	88.61	73.66	112.51	82.85	164.68	214.80	183.40	19.33	95.82	77.71	86.38	114.03
chr12	15822322	15822470	EPS8	83.62	134.09	76.93	78.07	121.91	81.13	143.74	174.91	147.64	33.69	89.07	91.03	80.13	104.65
chr12	15822599	15822759	EPS8	56.25	128.19	77.31	60.25	105.37	66.31	130.70	157.33	154.24	4.91	87.76	78.85	71.49	92.29
chr12	15823791	15823857	EPS8	111.00	225.73	138.07	107.94	189.78	139.24	242.19	265.39	258.61	3.31	169.12	142.72	127.24	166.09
chr12	15834161	15834236	EPS8	21.13	27.37	26.16	21.59	17.83	13.28	33.12	40.97	28.37	5.14	18.17	13.91	16.13	22.25
chr12	15835828	15835906	EPS8	16.48	31.43	16.14	11.54	26.29	18.39	35.24	30.97	43.25	0.65	28.91	37.33	26.53	24.72
chr12	65672550	65672645	MSRB3	32.04	27.71	25.09	32.03	55.88	26.88	36.60	36.88	34.39	90.70	37.26	20.30	46.07	37.98
chr12	65700644	65700806	MSRB3	0.00	1.56	1.22	0.00	1.47	0.05	0.00	0.00	0.75	0.39	0.00	0.00	0.00	0.45
chr12	65702310	65702435	MSRB3	88.17	159.61	94.71	103.73	143.67	82.55	177.02	298.78	188.07	136.09	105.75	92.84	104.71	139.25
chr12	65720607	65720714	MSRB3	94.80	121.98	84.61	84.55	125.23	67.26	111.06	148.32	137.86	45.16	88.68	69.87	92.19	98.28
chr12	65722307	65722383	MSRB3	24.10	60.58	28.12	27.92	33.53	34.99	54.87	72.10	58.88	0.00	24.90	25.82	32.81	37.15
chr12	65762779	65762806	MSRB3	24.96	57.86	36.93	20.04	26.71	33.32	48.46	35.11	47.89	4.96	28.50	38.43	27.57	33.60
chr12	65847509	65847605	MSRB3	73.42	128.31	79.74	85.12	113.28	68.79	130.30	226.06	141.59	79.63	104.95	70.56	94.78	108.48
chr12	65856936	65857102	MSRB3	34.12	58.36	36.84	33.44	58.22	30.24	53.32	73.96	70.66	62.92	40.20	23.75	41.59	48.00
chr12	80603240	80603290	OTOGL	38.61	53.25	33.06	28.33	40.92	36.20	44.82	70.18	78.06	0.00	39.55	40.02	32.33	41.92
chr12	80604628	80604666	OTOGL	37.23	62.77	45.82	42.92	44.46	42.44	76.31	77.54	78.82	0.00	32.77	46.69	35.38	48.98
chr12	80605730	80605777	OTOGL	70.71	124.56	61.52	70.27	97.81	59.60	100.29	156.46	125.42	42.77	101.15	66.40	68.04	89.75
chr12	80611379	80611444	OTOGL	32.06	65.36	40.50	24.67	48.76	33.42	59.17	93.27	74.38	1.00	41.61	34.97	33.59	45.76
chr12	80613595	80613692	OTOGL	50.16	105.78	84.20	56.65	72.63	56.88	123.71	149.66	125.10	5.08	63.70	72.66	66.36	80.52
chr12	80615872	80616025	OTOGL	96.92	179.84	100.03	89.99	141.45	89.63	163.74	262.06	191.74	34.53	119.18	107.42	110.32	131.38
chr12	80623038	80623158	OTOGL	23.92	48.73	35.46	25.32	44.83	20.88	56.65	74.50	64.28	2.63	27.46	26.40	25.51	37.59
chr12	80626673	80626877	OTOGL	80.73	126.98	57.40	59.76	112.29	63.19	99.64	192.83	133.60	19.69	92.56	74.63	85.50	92.77
chr12	80632632	80632758	OTOGL	92.53	132.29	95.65	99.57	130.48	92.31	130.73	181.57	153.09	65.28	111.47	79.58	89.43	113.71
chr12	80633114	80633219	OTOGL	46.81	109.54	60.06	47.72	82.11	66.01	114.01	153.78	138.63	0.90	62.34	64.74	66.73	78.89
chr12	80645474	80645579	OTOGL	35.38	66.07	31.75	30.63	48.42	36.08	72.48	105.43	71.34	7.96	46.48	40.75	37.80	49.40
chr12	80645857	80645981	OTOGL	55.27	110.05	94.10	55.76	73.02	57.30	87.98	149.06	111.24	8.84	60.63	51.02	62.86	76.19
chr12	80647247	80647354	OTOGL	77.43	126.32	87.73	62.98	113.28	69.09	146.78	220.15	160.94	23.68	79.69	84.88	81.92	104.41
chr12	80648305	80648350	OTOGL	19.80	47.61	26.33	12.11	23.50	28.37	29.00	30.52	43.43	0.00	15.98	23.98	14.33	25.05
chr12	80648821	80648965	OTOGL	41.57	62.19	35.00	20.99	46.30	30.41	62.46	93.88	73.08	5.25	39.52	39.63	36.83	45.86
chr12	80650118	80650240	OTOGL	54.78	96.66	67.66	71.76	77.65	51.56	102.27	165.50	141.00	10.89	82.48	67.76	71.23	82.50
chr12	80651606	80651782	OTOGL	26.65	56.12	33.41	40.93	45.72	32.15	57.87	84.97	51.56	0.29	31.55	30.90	38.70	41.01
chr12	80655750	80655873	OTOGL	61.52	112.33	81.66	68.99	108.52	70.31	122.06	161.59	149.16	16.44	83.49	76.19	69.59	92.69
chr12	80658782	80658990	OTOGL	47.53	71.32	37.72	48.12	58.64	38.29	71.25	96.93	67.08	41.88	49.06	43.44	48.38	55.94
chr12	80660232	80660396	OTOGL	33.06	47.36	23.55	22.02	35.51	22.18	53.09	65.44	60.04	26.62	29.55	33.78	34.04	37.68
chr12	80661034	80661107	OTOGL	27.49	47.16	32.26	23.69	36.30	27.15	50.76	58.28	62.15	15.50	40.70	37.46	40.59	38.24
chr12	80663883	80663934	OTOGL	66.37	77.65	72.23	61.83	69.40	58.15	81.96	137.67	114.37	17.44	75.69	53.31	63.65	73.84
chr12	80665429	80665590	OTOGL	65.15	108.44	58.91	60.31	95.15	55.42	106.41	138.69	112.95	38.97	64.13	67.99	71.69	81.04
chr12	80671949	80672055	OTOGL	45.42	100.85	47.51	49.00	68.64	51.32	82.05	117.17	95.27	1.00	71.24	52.14	51.87	65.13
chr12	80672809	80672946	OTOGL	65.30	107.58	62.80	64.24	91.99	53.46	113.44	183.80	133.49	7.82	76.51	77.78	75.31	86.52
chr12	80690608	80690741	OTOGL	20.48	35.57	20.81	23.83	18.24	25.54	32.34	53.59	37.20	0.00	18.19	30.19	13.82	26.33
chr12	80696415	80696563	OTOGL	6.79	28.05	18.50	12.23	19.58	10.34	22.53	37.50	32.19	0.00	17.10	11.46	16.42	18.02
chr12	80699357	80699475	OTOGL	66.48	131.49	79.97	62.08	93.80	61.65	130.60	152.51	135.40	0.96	77.00	68.30	62.46	88.35
chr12	80704392	80704507	OTOGL	36.28	60.97	34.41	27.19	33.89	31.03	51.66	76.59	55.42	5.39	37.87	37.32	24.59	40.67
chr12	80707257	80707412	OTOGL	60.40	91.34	60.63	50.42	83.24	51.90	103.54	155.56	106.62	0.75	57.54	53.47	54.08	72.95
chr12	80708086	80708111	OTOGL	18.42	28.69	36.85	10.23	19.85	24.77	30.12	29.96	35.85	0.54	22.08	31.08	9.65	24.04
chr12	80712327	80712493	OTOGL	34.70	56.68	45.13	31.47	48.45	36.84	68.82	58.86	68.11	9.17	41.59	28.24	44.07	44.00
chr12	80714203	80714480	OTOGL	71.45	148.06	79.49	88.41	98.64	65.52	144.24	193.14	147.66	16.95	85.55	76.89	84.82	101.33
chr12	80717504	80717620	OTOGL	86.00	167.42	97.85	91.42	132.29	113.11	162.44	228.08	213.50	37.68	108.65	97.86	78.23	128.02
chr12	80722446	80722524	OTOGL	79.90	160.77	90.52	72.57	107.75	87.84	151.94	216.11	155.24	15.97	104.52	100.59	79.77	111.98
chr12	80722832	80722899	OTOGL	67.26	183.26	93.87	73.79	132.44	95.91	129.75	228.24	192.93	10.18	103.59	99.57	93.16	117.57
chr12	80726786	80726858	OTOGL	59.07	94.73	65.21	61.11	96.34	71.44	97.90	173.86	115.23	2.48	69.36	67.23	57.25	81.16
chr12	80729744	80729920	OTOGL	61.64	96.08	68.36	58.71	89.94	72.46	87.43	123.09	121.83	33.98	69.01	54.36	67.90	78.07
chr12	80730194	80730335	OTOGL	11.94	51.48	33.85	24.69	35.80	30.17	60.46	70.52	55.82	2.42	34.97	25.44	21.38	36.46

chr12	80730578	80730600	OTOGL	3.70	25.65	23.09	3.17	7.00	17.04	22.04	24.83	32.74	1.00	6.96	17.83	6.30	15.42
chr12	80730693	80730784	OTOGL	14.15	46.09	39.64	11.57	27.55	23.09	45.97	49.11	51.95	0.00	22.25	33.14	14.97	30.37
chr12	80732856	80733044	OTOGL	73.31	133.52	109.48	76.08	99.32	92.94	128.66	172.33	171.76	11.60	104.83	84.61	77.41	104.87
chr12	80735729	80735942	OTOGL	30.28	50.57	36.48	29.06	41.13	27.23	58.99	73.79	58.78	0.00	27.27	35.62	25.13	39.10
chr12	80746076	80746216	OTOGL	33.30	65.88	49.42	35.96	63.01	38.71	83.50	130.93	75.97	2.42	35.45	37.82	38.05	54.36
chr12	80747106	80747290	OTOGL	76.65	110.98	55.91	50.90	88.60	56.72	89.59	155.55	111.64	31.39	79.62	69.17	74.57	81.39
chr12	80749517	80749728	OTOGL	69.85	120.69	81.86	69.94	101.87	82.99	125.11	185.61	131.81	58.81	78.73	74.18	79.32	98.46
chr12	80750197	80750300	OTOGL	16.41	38.50	11.36	11.13	27.84	16.38	38.40	41.08	39.97	2.90	26.09	12.60	25.77	23.55
chr12	80750588	80750694	OTOGL	73.52	149.17	94.51	72.13	86.75	61.81	140.40	170.13	162.35	4.37	87.98	89.47	78.31	99.38
chr12	80752029	80752129	OTOGL	39.67	80.07	41.21	39.27	43.78	43.99	75.82	118.00	93.12	7.11	41.13	41.58	40.40	55.40
chr12	80752452	80752555	OTOGL	38.39	64.52	53.46	39.08	49.87	41.48	84.14	112.37	83.61	5.01	30.72	44.13	40.95	53.90
chr12	80752641	80752680	OTOGL	13.00	15.33	16.23	7.53	7.63	7.98	22.65	13.90	26.33	1.00	2.48	11.08	6.50	12.09
chr12	80760355	80760417	OTOGL	18.78	35.57	21.17	13.46	22.73	31.21	29.22	39.08	39.38	0.00	22.49	21.71	23.95	24.57
chr12	80761342	80761519	OTOGL	20.85	35.82	25.65	14.12	19.96	20.79	47.33	42.52	31.05	0.00	12.88	17.89	19.20	24.07
chr12	80761986	80762089	OTOGL	75.22	130.17	88.86	81.94	106.72	87.69	139.68	205.94	119.10	29.01	90.84	99.05	68.94	104.52
chr12	80764351	80764469	OTOGL	26.37	33.12	27.92	17.13	21.52	23.05	46.45	59.62	51.76	0.00	35.77	19.77	18.95	30.21
chr12	80765800	80765844	OTOGL	31.02	54.67	41.93	25.16	30.09	27.44	50.33	56.67	67.49	0.00	31.22	36.93	20.40	37.75
chr12	80770904	80770982	OTOGL	32.53	47.25	38.70	34.80	32.82	34.29	53.08	63.38	60.67	0.00	42.94	30.41	43.59	39.24
chr12	80771629	80771828	OTOGL	63.58	92.79	78.04	53.72	92.40	60.50	111.21	161.14	108.21	17.83	67.37	71.94	76.64	81.56
chr12	122692929	122693124	DIABLO	50.42	49.59	54.94	54.35	110.68	42.64	63.89	106.08	70.95	125.84	52.97	41.45	62.17	68.65
chr12	122701036	122701131	DIABLO	121.33	220.81	145.31	119.54	196.23	133.72	237.97	281.57	275.94	55.96	139.71	158.02	122.40	173.84
chr12	122701307	122701416	DIABLO	76.88	139.39	65.43	69.85	96.67	82.85	148.58	206.02	152.35	3.43	89.56	90.95	78.87	101.83
chr12	122702814	122702944	DIABLO	100.58	153.67	113.94	95.24	129.41	94.02	159.26	260.16	169.57	11.74	115.62	90.02	120.92	124.44
chr12	122709060	122709295	DIABLO	124.01	153.44	110.28	130.06	191.70	110.75	172.27	256.48	222.71	186.89	144.98	103.96	139.78	158.96
chr12	122710513	122711200	DIABLO	8.75	10.90	6.47	9.87	16.49	5.91	9.42	12.38	8.72	22.23	8.07	5.57	9.45	10.40
chr12	133195404	133195575	P2RX2	55.31	76.02	39.38	48.49	75.23	53.60	55.32	72.15	79.63	121.35	61.99	49.29	74.03	65.65
chr12	133196026	133196160	P2RX2	70.53	118.26	72.36	91.36	140.72	86.34	108.46	119.74	141.56	218.50	84.58	68.25	109.67	110.05
chr12	133196266	133196336	P2RX2	89.93	110.97	81.54	97.96	192.39	111.01	104.27	184.35	143.45	229.56	93.96	78.11	137.82	126.46
chr12	133196431	133196505	P2RX2	74.64	86.19	49.49	63.53	124.01	60.29	85.89	113.91	102.47	160.47	60.57	50.84	86.21	86.03
chr12	133196587	133196682	P2RX2	18.60	25.86	19.38	17.64	40.06	16.61	15.77	25.75	24.98	55.54	17.08	11.63	23.30	24.08
chr12	133196854	133196933	P2RX2	56.84	84.74	61.48	62.68	103.43	68.86	73.73	131.31	91.80	153.19	88.39	56.96	76.64	86.12
chr12	133197032	133197174	P2RX2	56.99	58.71	56.52	61.57	93.66	41.47	67.66	89.73	73.63	151.22	62.43	46.42	59.64	71.67
chr12	133197588	133197717	P2RX2	55.92	70.45	45.20	70.32	121.88	62.59	77.15	112.95	86.05	169.53	73.37	45.12	80.29	82.55
chr12	133197842	133197950	P2RX2	85.34	126.25	108.33	115.14	173.85	97.36	153.80	218.91	150.45	256.06	103.59	95.54	106.33	140.38
chr12	133198062	133198558	P2RX2	49.90	71.93	52.84	73.68	108.16	58.03	82.98	113.48	91.40	174.65	73.47	56.96	78.79	83.96
chr13	20763041	20763742	GJB2	75.32	96.49	67.91	73.96	114.48	64.51	93.74	157.98	106.42	149.84	87.52	61.26	83.12	95.79
chr14	31344123	31344178	COCH	69.57	69.16	38.04	79.30	114.71	53.48	48.16	87.04	87.79	174.46	70.79	42.32	73.64	77.90
chr14	31344263	31344309	COCH	27.87	33.51	22.43	42.94	62.77	25.21	33.85	48.06	53.45	83.17	27.91	32.94	39.06	41.18
chr14	31346779	31346934	COCH	46.32	69.01	47.45	37.33	71.17	53.42	80.85	112.75	74.93	20.79	55.14	48.53	47.69	59.81
chr14	31348018	31348150	COCH	29.24	39.33	23.28	22.64	35.35	18.65	35.57	58.42	53.06	9.00	33.24	24.50	29.59	31.86
chr14	31348630	31348691	COCH	96.82	120.00	83.58	87.69	107.47	95.55	114.60	171.16	162.74	18.84	110.87	82.50	84.37	104.32
chr14	31349659	31349702	COCH	52.68	82.89	43.14	46.95	55.50	46.61	94.52	118.18	88.43	22.11	53.52	43.00	61.39	62.30
chr14	31349794	31349940	COCH	80.63	134.29	81.39	77.00	107.03	76.95	109.52	177.92	152.02	80.36	90.14	78.68	104.24	103.83
chr14	31353760	31353862	COCH	64.84	95.94	62.62	62.60	93.09	52.72	117.38	136.62	101.52	3.83	78.18	61.19	71.27	77.55
chr14	31354601	31354826	COCH	97.87	157.78	101.58	90.64	137.28	87.89	145.31	242.81	171.43	111.97	121.25	84.25	101.93	129.17
chr14	31355003	31355518	COCH	110.63	174.37	122.70	121.53	154.48	113.24	186.58	290.76	199.03	100.92	126.84	121.59	122.51	151.89
chr14	31358823	31358997	COCH	61.74	88.49	56.58	61.29	92.25	51.40	105.09	151.14	118.98	3.42	59.47	56.10	58.30	75.49
chr14	76870740	76870802	ESRRB	0.00	0.00	0.00	1.00	0.00	0.00	0.00	0.14	0.00	0.00	0.70	0.00	2.00	0.15
chr14	76873887	76873939	ESRRB	0.00	0.00	1.40	1.00	1.00	0.79	0.19	0.00	0.00	0.00	0.00	1.00	0.00	0.45
chr14	76905685	76906093	ESRRB	61.94	84.33	55.54	88.07	143.40	75.50	77.12	122.12	87.49	169.91	70.00	54.18	83.31	90.80
chr14	76928889	76929004	ESRRB	56.07	62.66	47.95	49.93	90.16	45.06	62.69	71.00	60.11	129.54	61.06	39.76	71.07	64.67
chr14	76948360	76948469	ESRRB	64.66	90.48	69.51	80.86	111.85	79.05	108.13	134.84	130.91	196.97	90.60	63.84	80.85	101.81
chr14	76948942	76949102	ESRRB	31.06	40.55	21.76	31.40	42.19	20.73	41.80	53.60	52.01	78.13	37.07	26.30	43.75	39.72
chr14	76957791	76958059	ESRRB	15.12	15.96	17.83	22.62	40.00	14.32	19.54	24.38	25.51	50.24	22.54	8.53	21.79	23.05
chr14	76964558	76964795	ESRRB	61.49	93.80	47.40	72.17	115.81	58.95	94.14	125.01	99.85	182.63	67.20	46.17	76.58	88.72
chr14	76966207	76966409	ESRRB	31.85	43.47	31.99	36.05	53.55	31.09	51.72	42.87	49.04	64.55	41.45	28.06	48.41	42.14
chr14	76967039	76967064	ESRRB	82.62	113.62	88.23	116.04	145.04	99.15	121.38	201.77	148.42	200.85	105.54	96.00	129.12	126.55
chr15	43891871	43891958	STRC	0.00	0.00	0.00	0.13	0.00	0.28	0.01	0.06	0.00	0.00	0.00	0.00	0.05	0.04
chr15	43892159	43892305	STRC	64.84	84.63	55.95	77.69	141.34	61.48	73.57	132.91	114.69	156.22	106.73	54.90	83.56	93.75
chr15	43892395	43892491	STRC	19.27	22.92	21.85	12.97	31.41	16.21	20.75	32.38	36.57	42.73	22.79	17.33	22.38	24.76
chr15	43892733	43892880	STRC	31.84	32.41	18.97	34.15	56.66	19.85	38.55	54.47	54.64	77.22	40.83	20.33	28.28	39.99
chr15	43893071	43893212	STRC	26.24	19.75	10.82	32.06	46.15	11.77	18.12	41.80	48.21	57.07	42.73	14.33	42.65	30.75
chr15	43893595	43893749	STRC	40.22	36.90	24.94	34.44	58.04	30.19	42.48	59.90	73.09	44.94	39.15	32.57	44.02	43.07
chr15	43895441	43895609	STRC	56.62	55.57	41.76	45.40	79.05	46.31	65.75	88.73	103.68	86.06	43.64	61.99	65.99	64.55
chr15	43896195	43896350	STRC	20.09	15.70	8.46	14.96	26.42	9.29	17.63	21.47	29.55	37.35	20.94	14.63	22.69	19.71
chr15	43896560	43896649	STRC	22.47	26.27	25.34	28.07	41.36	22.17	38.83	55.72	62.93	39.27	25.32	25.88	23.56	34.47
chr15	43896849	43897044	STRC	112.55	136.86	90.98	118.43	177.06	87.18	134.62	199.37	214.18	178.80	123.44	79.14	121.17	137.72
chr15	43897463	43897597	STRC	43.36	80.92	48.07	48.76	80.59	33.16	74.44	120.87	136.29	101.31	62.03	42.34	60.81	72.68
chr15	43900062	43900173	STRC	28.70	47.79	33.12	42.24	64.56	30.53	42.60	60.66	53.03	72.89	53.38	12.67	40.62	45.18
chr15	43900279	43900401	STRC	39.11	46.49	48.12	44.55	73.50	27.52	57.52	72.25	65.30	93.98	69.05	14.80	58.61	54.35
chr15	43901475	43901532	STRC	99.50	124.10	83.38	99.28	150.78	90.00	134.05	167.50	137.24	146.88	118.90	34.74	114.62	115.53
chr15	43902511	43902635	STRC	6.30	8.90	5.90	1.55	5.41	3.14	8.53	12.77	6.62	9.16	3.24	5.18	7.76	6.39
chr15	43903118	43903182	STRC	103.26	189.51	149.05	105.02	195.38	138.72	114.12	188.12	116.32	244.02	108.11	121.98	109.32	147.80
chr15	43903347	43903513	STRC	2.66	4.80	3.44	1.58	2.09	1.69	4.25	3.95	3.65	5.40	1.79	3.35	2.89	3.22
chr15	43903698	43903735	STRC	3.00	12.87	0.00	6.74	9.50	0.00	0.00	18.53	4.29	0.13	5.08	0.00	0.00	5.01
chr15	43904041	43904222	STRC	0.00	0.00	0.00	0.00	0.13	0.00	0.00	0.04	0.00	0.00	0.00	0.00	0.00	0.01
chr15	43904576	43904707	STRC	0.00	0.00	0.00	0.00	0.00	0.00	0.00	0.00	0.00	0.00	0.00	0.00	0.00	0.00
chr15	43904990	43905104	STRC	0.00	0.00	0.00	0.00	0.00	0.00	0.00	0.00	0.00	0.00	0.00	0.00	0.00	0.00
chr15	43905244	43905429	STRC	0.93	0.25	0.00	0.87	0.05	0.19	0.40	2.87	1.27	0.74	0.39	0.78	0.62	0.73
chr15	43906086	43906251	STRC	0.00	0.00	0.00	0.00	0.00	0.00	0.00	0.00	0.00	0.00	0.00	0.00	0.00	0.00

chr15	43906395	43906510	STRC	0.26	0.47	0.31	0.27	0.34	0.66	0.72	0.88	1.99	0.00	0.37	0.10	0.00	0.53
chr15	43906613	43906674	STRC	8.94	6.29	0.00	10.77	6.10	5.82	8.29	24.81	6.02	6.53	8.56	5.79	9.42	8.16
chr15	43907632	43908888	STRC	0.00	0.00	0.00	0.00	0.00	0.00	0.02	0.00	0.00	0.00	0.02	0.00	0.00	0.00
chr15	43909657	43909680	STRC	0.00	0.00	0.00	0.00	0.00	0.00	0.00	0.00	0.00	0.00	0.00	0.00	0.00	0.00
chr15	43909770	43910554	STRC	24.52	30.14	22.23	31.72	42.69	23.49	27.28	40.15	32.88	73.25	33.64	18.52	31.80	33.38
chr15	43910858	43910920	STRC	0.00	0.00	0.00	0.00	0.00	0.00	0.00	0.00	0.00	0.00	0.00	0.00	0.00	0.00
chr15	78397654	78397674	CIB2	46.19	33.62	38.62	37.29	62.33	25.90	54.71	60.86	53.48	105.14	46.05	36.10	50.48	50.02
chr15	78398082	78398276	CIB2	119.21	138.77	100.04	127.44	208.58	107.35	147.64	199.96	160.09	282.90	152.93	105.96	149.11	154.24
chr15	78401578	78401851	CIB2	72.48	85.12	67.91	67.87	107.47	61.44	106.90	118.64	101.89	150.75	82.16	55.93	92.00	89.88
chr15	78403508	78403618	CIB2	56.97	66.70	43.27	53.86	93.56	45.79	60.59	79.07	87.22	134.27	75.67	39.13	69.07	69.67
chr15	78416048	78416081	CIB2	35.65	26.21	19.18	36.85	37.91	27.88	30.18	45.82	32.82	60.47	33.65	18.91	29.12	33.79
chr15	78423508	78423877	CIB2	44.48	72.38	45.79	24.84	60.03	48.81	68.55	67.33	75.38	63.68	44.38	48.57	57.72	55.35
chr15	83518468	83518655	HOMER2	40.37	60.49	37.13	56.20	87.96	39.26	66.87	89.37	70.41	89.31	59.03	33.98	55.65	60.87
chr15	83519904	83519983	HOMER2	41.01	79.41	47.51	45.38	82.44	44.51	67.34	117.30	93.30	43.58	44.08	49.06	61.16	62.91
chr15	83520895	83521004	HOMER2	60.80	90.65	56.53	68.88	93.49	53.22	96.90	125.33	94.19	57.35	75.23	66.63	78.98	78.27
chr15	83523397	83523552	HOMER2	29.56	35.93	22.46	33.77	61.74	31.81	33.47	47.19	43.22	105.72	35.73	34.01	47.33	42.88
chr15	83527782	83527920	HOMER2	48.76	56.07	29.29	42.00	60.80	48.98	52.13	74.51	73.32	85.35	48.52	39.68	57.05	54.95
chr15	83532920	83533011	HOMER2	111.36	189.09	119.04	105.73	145.85	117.73	214.55	285.37	226.30	34.16	132.26	133.35	108.83	151.23
chr15	83544026	83544156	HOMER2	23.98	27.23	14.53	23.44	28.52	17.59	33.79	46.37	39.44	37.62	30.37	21.61	26.18	28.71
chr15	83561438	83561593	HOMER2	101.97	132.29	91.14	113.89	163.87	101.08	126.85	205.99	154.15	216.51	126.79	69.87	142.96	133.70
chr15	83621284	83621287	HOMER2	5.00	3.00	8.00	15.00	9.25	8.00	5.00	6.00	12.50	15.25	6.00	4.00	6.00	8.08
chr16	2546036	2547114	TBC1D24	63.12	74.19	55.05	76.43	123.13	63.08	87.92	118.07	85.57	186.57	72.34	51.36	89.69	88.07
chr16	2547712	2547728	TBC1D24	85.00	123.94	88.65	69.06	136.06	79.71	149.82	197.59	164.59	128.88	121.53	68.24	93.06	117.75
chr16	2548240	2548397	TBC1D24	38.19	31.96	34.11	48.01	69.12	22.78	45.85	66.08	55.25	104.89	40.13	23.04	37.18	48.29
chr16	2549359	2549421	TBC1D24	55.10	45.06	42.70	52.83	99.16	40.46	62.79	84.56	65.95	111.43	47.21	40.44	68.16	62.31
chr16	2549837	2549931	TBC1D24	83.98	120.56	77.96	129.55	171.95	69.62	116.17	170.33	105.31	251.63	89.97	73.48	114.03	121.71
chr16	2550270	2550491	TBC1D24	28.36	22.23	10.71	27.62	40.16	17.26	22.73	26.13	33.58	41.15	19.98	15.87	25.09	25.48
chr16	2550806	2550959	TBC1D24	94.99	114.04	85.33	127.34	204.57	98.71	127.83	182.79	128.49	278.58	114.88	97.12	126.44	137.89
chr16	21689837	21689926	OTOA	36.72	61.43	38.57	54.80	59.22	47.64	58.21	125.04	91.37	20.84	53.33	40.72	48.22	57.33
chr16	21690237	21690264	OTOA	27.93	69.57	42.18	24.50	38.82	35.79	51.93	103.43	70.89	0.00	29.50	44.00	28.14	44.88
chr16	21690358	21690387	OTOA	38.33	86.70	60.37	45.03	69.87	51.37	98.93	155.00	109.03	0.00	61.50	56.93	51.90	69.42
chr16	21690506	21690532	OTOA	45.59	87.19	47.19	47.00	51.44	41.33	87.78	91.07	88.67	0.00	42.33	50.59	54.81	56.68
chr16	21693060	21693146	OTOA	49.09	62.33	37.47	49.31	73.59	50.00	64.70	102.51	81.15	80.75	52.46	37.09	54.46	61.70
chr16	21695845	21695873	OTOA	39.72	49.14	22.17	47.00	44.10	28.38	41.03	64.24	51.21	74.79	41.14	26.52	32.17	44.12
chr16	21696552	21696682	OTOA	32.13	59.26	27.69	43.09	73.83	41.40	41.00	63.85	70.13	88.86	52.83	34.16	54.26	52.35
chr16	21698735	21698969	OTOA	37.42	52.04	39.60	39.95	65.74	45.82	55.08	72.31	62.46	70.82	43.72	34.20	46.14	51.60
chr16	21702906	21703008	OTOA	44.18	61.25	44.29	57.70	69.59	31.57	75.78	90.91	68.75	51.19	59.66	39.11	52.64	57.83
chr16	21709097	21709196	OTOA	55.63	116.89	61.45	56.68	90.00	48.78	115.71	164.48	135.62	20.92	80.87	65.15	70.91	84.35
chr16	21712210	21712348	OTOA	25.83	48.20	38.04	36.33	51.91	34.08	47.27	68.94	55.26	64.28	41.02	36.50	49.23	45.64
chr16	21716352	21716358	OTOA	6.29	9.00	16.00	10.71	6.71	19.86	6.00	11.00	9.14	3.00	6.43	9.00	5.00	9.43
chr16	21716491	21716613	OTOA	21.89	29.38	21.92	34.82	42.59	27.64	44.20	59.34	42.59	72.46	33.20	18.28	36.67	37.36
chr16	21721210	21721424	OTOA	29.78	44.82	37.80	34.17	62.41	38.69	34.51	67.87	62.77	87.54	48.21	31.86	53.56	48.37
chr16	21726307	21726473	OTOA	47.29	50.79	34.19	62.02	81.81	46.02	59.11	96.84	80.65	120.13	58.60	36.74	47.49	64.52
chr16	21728229	21728368	OTOA	39.49	61.96	40.28	42.79	80.59	33.52	67.34	108.56	66.41	13.48	55.05	40.14	55.59	54.13
chr16	21730455	21730512	OTOA	18.83	33.64	24.64	12.03	35.62	16.81	19.93	45.59	31.90	23.43	21.19	11.00	29.12	24.55
chr16	21730709	21730825	OTOA	56.60	93.11	63.79	72.29	92.55	52.83	96.26	141.72	122.59	101.15	72.40	53.75	64.46	84.92
chr16	21734227	21734299	OTOA	106.21	172.96	107.99	113.55	137.05	106.55	164.30	253.51	175.59	79.33	111.82	106.19	122.19	136.25
chr16	21737845	21737979	OTOA	38.64	33.82	28.19	36.31	45.68	21.13	49.97	48.94	46.31	61.14	48.44	30.42	48.64	40.75
chr16	21739563	21739752	OTOA	58.50	83.54	42.71	79.39	98.10	61.05	105.22	117.91	81.44	174.41	91.40	61.69	78.19	87.95
chr16	21742159	21742251	OTOA	60.59	101.86	60.80	51.65	82.20	62.19	98.95	152.27	91.81	77.17	64.90	83.72	34.68	82.34
chr16	21747583	21747711	OTOA	58.56	93.05	52.81	62.16	111.88	57.87	77.69	162.72	100.12	58.57	90.05	56.42	83.05	81.83
chr16	21752043	21752229	OTOA	0.00	0.00	0.00	0.00	0.00	0.00	0.00	0.00	0.00	0.00	0.00	0.00	0.00	0.00
chr16	21756203	21756357	OTOA	0.00	0.00	0.00	0.00	0.00	0.00	0.00	0.00	0.00	0.00	0.00	0.00	0.00	0.00
chr16	21763257	21763398	OTOA	0.00	0.00	0.00	0.00	0.00	0.00	0.61	0.00	0.59	0.00	0.02	0.00	2.08	0.10
chr16	21763691	21763826	OTOA	6.66	6.10	3.25	7.84	2.71	2.84	15.96	12.60	8.13	15.40	3.24	2.18	0.67	7.24
chr16	21764349	21764445	OTOA	48.42	44.38	48.91	48.16	13.89	45.94	71.32	44.31	18.92	32.88	45.05	29.88	11.24	41.00
chr16	21768404	21768598	OTOA	0.18	0.00	0.00	0.34	0.14	0.15	0.34	0.00	0.26	0.00	0.00	0.61	0.00	0.17
chr16	21771792	21771861	OTOA	41.39	37.89	39.21	34.47	30.34	30.30	65.80	59.80	36.56	84.86	36.83	30.59	34.09	44.00
chr16	75661794	75661891	KARS	86.92	104.14	82.59	89.84	129.37	77.39	127.40	161.51	121.20	27.03	81.11	86.39	86.43	97.91
chr16	75662468	75662610	KARS	28.08	39.44	26.98	23.64	35.48	18.66	37.56	44.67	40.66	32.44	26.95	20.48	30.08	31.25
chr16	75663314	75663439	KARS	39.38	49.21	31.42	41.16	63.67	40.44	59.46	93.87	59.56	67.37	52.14	30.40	59.53	52.34
chr16	75664322	75664406	KARS	38.13	63.09	35.95	40.07	51.09	43.48	55.40	77.76	61.18	32.80	55.24	39.71	43.29	49.49
chr16	75665067	75665151	KARS	37.98	48.07	37.04	36.89	55.48	32.39	59.07	58.02	49.47	60.91	55.19	30.24	50.41	46.73
chr16	75665315	75665487	KARS	82.69	121.49	69.93	90.02	110.17	72.38	123.17	191.98	122.18	145.82	88.24	78.29	86.79	108.03
chr16	75665592	75665753	KARS	46.59	59.68	41.11	61.00	69.08	49.78	72.44	110.20	88.66	89.47	57.35	41.53	67.59	65.57
chr16	75668072	75668190	KARS	27.66	49.04	46.01	29.98	64.29	32.29	59.78	82.07	75.01	6.08	41.15	36.80	39.83	45.85
chr16	75669579	75669703	KARS	45.78	66.56	50.39	54.77	61.55	42.62	72.28	121.43	84.82	15.60	59.05	53.57	52.21	60.70
chr16	75669811	75669996	KARS	102.07	159.27	96.51	89.03	138.80	80.44	167.59	205.02	180.08	40.26	121.82	98.56	101.07	123.29
chr16	75670353	75670445	KARS	50.37	87.90	73.10	68.04	93.41	58.12	101.65	122.53	122.28	59.28	85.15	64.42	64.12	82.19
chr16	75674083	75674247	KARS	16.84	40.55	22.46	20.54	39.32	22.53	30.01	61.32	30.31	35.91	31.10	23.01	25.30	31.16
chr16	75675463	75675621	KARS	36.51	57.71	29.12	44.50	67.20	46.01	78.74	87.20	61.06	56.96	40.36	39.20	42.26	53.72
chr16	75678182	75678360	KARS	78.85	119.79	61.72	69.73	100.21	62.63	98.73	116.05	109.42	137.78	94.60	66.32	85.74	92.99
chr16	75681477	75681537	KARS	84.25	101.44	84.05	98.67	151.16	90.52	105.90	131.31	123.51	206.23	97.16	77.70	111.44	112.66
chr17	18021897	18025723	MYO15A	67.90	82.48	61.84	74.73	120.97	64.38	84.60	100.96	92.22	171.60	75.78	60.17	84.63	88.13
chr17	18027798	18027879	MYO15A	59.45	73.45	53.96	73.22	100.49	67.46	75.88	129.24	108.91	164.49	76.95	51.27	84.61	86.23
chr17	18028483	18028545	MYO15A	57.25	77.67	53.75	51.54	83.60	66.22	86.02	127.29	79.46	93.52	66.19	52.06	65.11	74.55
chr17	18029662	18029770	MYO15A	48.21	55.40	41.30	77.43	90.58	52.39	65.19	82.50	82.41	145.09	68.53	50.51	67.94	71.63
chr17	18030106	18030179	MYO15A	49.88	79.62	39.66	76.34	87.04	46.05	81.72	108.15	82.18	137.39	66.73	37.26	61.51	74.33
chr17	18030390	18030483	MYO15A	73.28	93.90	60.64	69.20	108.38	54.73	92.40	121.59	114.15	169.57	75.84	58.54	75.82	91.02
chr17	18034552	18034656	MYO15A	40.54	71.25	47.64	78.87	79.83	57.60	70.92	87.97	73.31	149.08	73.12	39.89	53.44	72.50

chr17	18034783	18034845	MYO15A	32.97	48.95	36.73	33.68	56.57	31.37	40.24	39.92	47.78	73.16	38.41	24.70	34.46	42.04
chr17	18035768	18035880	MYO15A	45.28	75.12	60.93	51.31	107.97	51.00	64.50	117.28	76.38	148.92	64.96	41.97	70.19	75.47
chr17	18036540	18036700	MYO15A	38.17	40.84	28.10	34.30	67.20	31.80	39.20	62.84	42.58	76.32	42.26	24.53	40.16	44.01
chr17	18039026	18039138	MYO15A	32.91	46.31	30.58	36.90	47.49	23.34	40.61	44.69	38.53	67.44	35.85	24.11	36.96	39.06
chr17	18039732	18039789	MYO15A	99.38	115.86	95.71	110.60	186.76	108.84	122.52	166.95	156.97	260.10	121.50	89.50	129.28	136.22
chr17	18039878	18040000	MYO15A	38.25	79.47	65.24	66.67	107.03	53.35	70.89	119.95	94.05	149.27	65.85	62.67	74.11	81.06
chr17	18040899	18040993	MYO15A	18.42	27.27	22.43	22.07	30.77	17.27	24.89	36.20	34.12	34.09	22.61	15.51	24.73	25.47
chr17	18041430	18041560	MYO15A	61.53	65.60	58.15	74.78	117.06	53.75	76.09	141.87	80.05	138.73	71.83	46.65	87.39	82.17
chr17	18042126	18042250	MYO15A	40.03	29.11	20.02	37.44	59.32	30.44	30.85	49.92	42.90	78.96	36.95	23.82	44.13	39.98
chr17	18042849	18042925	MYO15A	46.16	53.60	46.38	53.47	81.14	47.13	60.13	84.10	66.17	110.92	55.04	41.03	60.47	62.10
chr17	18043832	18043979	MYO15A	47.05	68.25	49.01	70.98	123.84	59.24	83.34	94.29	91.49	160.57	69.11	52.00	78.68	80.76
chr17	18044102	18044146	MYO15A	43.16	41.47	43.04	46.42	90.56	42.80	66.69	80.18	73.04	122.87	57.49	38.84	49.02	62.21
chr17	18044334	18044457	MYO15A	30.77	40.25	40.66	38.83	67.44	34.64	47.72	69.90	47.19	75.06	55.24	37.31	40.85	48.75
chr17	18044968	18045084	MYO15A	70.11	61.46	45.44	63.61	99.61	44.97	86.67	101.12	68.78	145.77	65.08	54.99	70.63	75.63
chr17	18045394	18045568	MYO15A	30.26	17.70	11.13	25.76	42.09	13.79	26.82	34.49	25.35	37.54	27.63	14.77	29.65	25.61
chr17	18046071	18046154	MYO15A	27.54	36.60	25.12	37.57	37.81	32.38	47.48	43.14	39.95	112.44	37.11	32.25	46.96	42.45
chr17	18046881	18046933	MYO15A	11.55	18.75	12.40	11.85	30.17	14.83	23.47	33.92	22.68	41.75	18.96	16.62	16.23	21.41
chr17	18047030	18047110	MYO15A	43.52	50.68	43.23	54.10	73.63	43.43	45.59	95.88	60.72	114.64	52.14	28.78	39.99	58.86
chr17	18047185	18047314	MYO15A	34.38	38.27	37.34	45.10	62.15	37.95	53.52	76.72	57.16	83.01	51.58	26.94	49.18	50.34
chr17	18047812	18047906	MYO15A	26.39	23.71	18.01	31.05	49.25	22.81	31.59	32.57	32.49	77.72	27.80	15.92	22.98	32.44
chr17	18049187	18049421	MYO15A	65.18	76.48	59.39	57.34	106.22	58.55	94.65	125.08	85.13	160.37	65.09	55.84	74.63	84.11
chr17	18051344	18051524	MYO15A	73.82	73.00	45.19	71.14	112.14	51.94	82.64	109.63	83.53	173.08	63.11	46.58	80.93	82.15
chr17	18051811	18051882	MYO15A	38.08	50.89	29.26	34.92	69.67	38.83	65.63	84.19	70.46	97.10	42.22	32.68	56.54	54.49
chr17	18052076	18052266	MYO15A	37.86	54.25	28.52	37.02	65.46	27.81	53.06	72.25	61.98	99.04	41.23	27.49	40.10	50.50
chr17	18052531	18052690	MYO15A	50.90	57.54	45.69	40.37	58.66	33.04	58.29	83.54	58.96	87.63	49.97	36.99	55.54	55.13
chr17	18052801	18052894	MYO15A	44.88	51.44	33.47	40.36	59.01	33.48	53.36	61.33	57.44	73.03	52.84	29.09	39.28	49.14
chr17	18053744	18053857	MYO15A	67.39	55.31	48.80	61.41	103.54	55.17	59.29	100.07	78.11	158.97	74.18	44.30	67.11	75.54
chr17	18054013	18054079	MYO15A	17.69	23.22	13.39	33.28	38.33	15.49	14.76	31.94	17.34	50.13	19.75	17.58	23.96	24.41
chr17	18054151	18054227	MYO15A	10.99	18.90	25.69	13.53	14.44	31.66	13.53	15.99	13.12	12.43	11.39	6.96	8.53	15.72
chr17	18054425	18054604	MYO15A	91.09	125.22	92.77	118.86	184.06	96.33	112.37	150.29	145.29	283.98	112.57	98.78	121.35	134.30
chr17	18054710	18054841	MYO15A	78.61	89.01	82.01	91.48	146.21	66.14	112.69	152.94	100.84	186.85	111.13	87.67	85.83	108.80
chr17	18055161	18055265	MYO15A	41.09	45.88	27.30	36.08	46.12	35.89	37.19	57.73	41.74	68.36	34.58	25.47	39.50	41.45
chr17	18055427	18055498	MYO15A	22.68	33.54	19.94	33.69	40.43	28.13	29.08	45.54	27.74	68.40	21.90	14.75	21.38	32.15
chr17	18057090	18057210	MYO15A	57.73	60.45	61.16	71.96	97.74	49.50	48.56	72.07	61.64	165.36	64.39	35.98	50.98	70.55
chr17	18057446	18057504	MYO15A	56.80	60.54	48.32	67.95	101.25	46.98	61.90	92.90	65.76	167.10	80.93	44.19	67.05	74.55
chr17	18057995	18058069	MYO15A	35.31	30.84	24.48	21.11	60.35	24.79	37.91	47.63	46.15	58.32	39.57	23.35	29.43	37.48
chr17	18058425	18058539	MYO15A	70.73	86.28	71.03	78.26	126.67	50.80	91.68	94.13	105.37	171.13	69.17	58.33	90.47	89.46
chr17	18058629	18058746	MYO15A	55.69	43.96	37.68	58.89	108.73	36.24	50.20	67.51	52.88	108.18	48.78	34.53	58.18	58.61
chr17	18059510	18059650	MYO15A	40.31	47.42	26.22	47.51	67.12	30.76	43.45	64.70	50.02	79.83	47.83	28.48	45.72	47.80
chr17	18060269	18060379	MYO15A	31.71	39.57	28.76	30.91	80.28	37.09	38.49	56.59	48.94	95.81	39.08	33.59	38.35	46.73
chr17	18060471	18060544	MYO15A	67.54	109.34	63.50	81.47	133.65	70.46	100.81	155.04	99.46	163.32	67.22	73.31	90.24	98.76
chr17	18061037	18061214	MYO15A	39.00	76.76	40.52	43.33	79.08	39.72	73.66	97.49	89.65	129.47	47.53	45.92	51.19	66.85
chr17	18061838	18061952	MYO15A	153.42	206.06	141.77	162.52	262.31	139.19	228.37	305.81	239.63	421.19	176.12	132.29	186.42	214.06
chr17	18062240	18062311	MYO15A	44.36	53.00	34.79	39.53	59.93	36.94	46.60	58.01	58.79	97.63	60.69	26.18	50.29	51.37
chr17	18062590	18062661	MYO15A	47.42	70.78	40.42	56.51	94.53	49.51	61.11	67.25	71.88	124.46	65.97	49.89	60.54	66.64
chr17	18062921	18062993	MYO15A	48.25	52.00	41.59	40.40	51.30	37.08	38.81	93.04	69.10	76.77	43.29	24.10	41.78	51.31
chr17	18063250	18063331	MYO15A	46.38	65.22	55.98	62.07	93.62	52.91	78.71	110.61	90.12	128.48	60.88	56.10	60.48	75.09
chr17	18064632	18064761	MYO15A	42.53	60.96	34.69	58.82	99.44	48.94	60.54	77.89	68.78	141.62	58.69	49.35	70.53	66.85
chr17	18065900	18065993	MYO15A	52.95	40.31	34.39	68.27	96.11	42.70	50.13	76.28	57.44	155.29	65.31	37.95	66.15	64.76
chr17	18066559	18066635	MYO15A	42.71	32.19	20.65	27.97	38.86	21.99	40.13	51.64	47.21	60.70	23.47	10.05	27.83	34.80
chr17	18067057	18067152	MYO15A	29.24	54.76	37.17	51.35	80.38	42.74	35.59	56.43	42.28	105.36	34.74	24.05	51.60	49.51
chr17	18069676	18069835	MYO15A	58.56	63.96	43.09	47.70	99.12	50.29	61.81	105.75	69.11	132.86	62.93	38.30	52.98	69.46
chr17	18070905	18071037	MYO15A	45.73	56.22	45.90	55.38	94.35	60.98	77.45	98.30	77.17	133.16	63.13	37.90	59.00	70.47
chr17	18074953	18075085	MYO15A	28.70	39.16	30.51	34.45	55.69	19.85	28.05	42.15	36.74	60.88	29.62	16.01	33.29	35.15
chr17	18075472	18075604	MYO15A	42.73	44.59	34.23	55.62	67.53	46.98	43.74	72.52	61.14	97.55	56.85	39.31	51.68	55.23
chr17	18077096	18077235	MYO15A	62.15	92.12	63.45	79.82	109.80	53.54	87.47	131.06	96.31	148.87	79.87	58.58	75.00	88.59
chr17	18082084	18082184	MYO15A	47.62	44.84	40.30	50.76	71.20	30.37	51.72	81.38	63.56	97.83	44.20	27.66	48.40	54.29
chr17	79477506	79477859	ACTG1	17.86	29.41	15.90	28.89	44.19	21.90	20.10	35.70	18.86	56.82	19.41	19.56	24.03	27.38
chr17	79477954	79478134	ACTG1	38.52	43.67	34.49	47.86	70.75	38.69	45.12	77.23	72.84	90.17	46.54	38.64	50.82	53.71
chr17	79478215	79478652	ACTG1	42.98	49.42	40.72	59.72	81.64	46.95	58.72	78.88	65.09	133.66	54.74	42.50	57.00	62.92
chr17	79478930	79479168	ACTG1	53.28	69.25	46.54	79.89	96.35	55.09	70.69	93.54	85.59	141.23	60.42	46.09	77.31	74.83
chr17	79479259	79479386	ACTG1	109.76	166.02	120.62	155.80	249.28	124.52	161.99	206.68	191.84	403.37	162.88	124.86	175.04	181.47
chr17	79479761	79479892	ACTG1	0.17	0.79	1.10	1.62	1.56	0.86	0.63	0.00	1.71	2.56	0.37	0.00	0.66	0.95
chr18	44057436	44057915	LOXHD1	45.75	60.78	38.42	53.35	84.23	45.37	62.52	91.74	60.47	119.61	57.03	37.19	62.80	63.04
chr18	44063551	44063708	LOXHD1	19.08	32.16	21.94	31.92	50.77	22.50	21.54	51.56	40.13	54.89	31.46	29.92	23.61	33.99
chr18	44064983	44065114	LOXHD1	46.70	77.55	50.13	49.25	76.15	50.61	77.64	100.86	75.84	99.64	46.58	52.28	51.45	66.94
chr18	44068936	44069105	LOXHD1	40.26	44.36	34.49	48.34	52.58	35.98	43.47	71.36	55.66	103.52	47.33	41.25	33.19	51.55
chr18	44085802	44085986	LOXHD1	67.59	73.47	66.05	57.04	82.59	72.64	74.38	113.98	84.74	148.25	79.94	46.66	70.04	80.61
chr18	44087502	44087675	LOXHD1	68.42	121.97	81.49	95.84	123.08	99.20	124.38	180.39	128.99	200.14	93.16	58.93	102.66	114.67
chr18	44089662	44089778	LOXHD1	19.24	27.29	10.85	18.96	27.38	14.62	21.46	22.03	31.32	47.41	25.09	17.13	16.11	23.57
chr18	44098093	44098219	LOXHD1	30.61	33.30	27.19	32.05	60.63	28.28	37.30	66.72	50.17	51.30	39.93	25.94	28.95	40.28
chr18	44102065	44102272	LOXHD1	37.59	55.74	53.99	49.43	78.91	50.39	58.94	79.54	68.33	125.47	76.09	52.82	57.47	65.60
chr18	44104430	44104564	LOXHD1	65.41	50.81	46.10	58.87	85.32	51.03	72.54	96.10	73.84	115.10	62.94	39.55	72.47	68.13
chr18	44104672	44104880	LOXHD1	58.57	72.95	61.16	75.36	83.02	63.70	97.53	120.23	90.45	172.99	78.75	46.29	101.20	85.08
chr18	44109141	44109294	LOXHD1	56.14	86.12	56.37	63.57	82.89	56.18	78.98	141.01	83.73	115.45	77.75	45.49	73.00	78.64
chr18	44113126	44113287	LOXHD1	37.72	68.89	47.66	41.38	65.37	35.78	74.98	102.15	70.94	60.09	46.99	30.80	40.00	56.90
chr18	44114299	44114414	LOXHD1	35.01	58.14	37.21	42.01	45.78	36.66	52.34	68.68	61.49	60.69	49.69	37.28	45.59	48.75
chr18	44118120	44118300	LOXHD1	40.56	43.58	34.44	26.23	66.03	29.46	50.86	64.53	56.29	55.68	44.61	28.83	33.57	45.09
chr18	44121740	44121903	LOXHD1	43.90	54.88	35.50	45.14	67.01	56.95	57.76	84.30	65.80	120.59	56.96	49.28	58.75	61.51
chr18	44122691	44122818	LOXHD1	30.80	50.39	24.52	44.05	63.26	39.23	59.64	61.40	45.73	55.87	42.47	26.84	31.00	45.35

chr18	44125281	44125384	LOXHD1	110.27	140.61	105.43	92.40	136.27	86.10	179.96	234.26	190.88	49.46	125.61	102.35	108.42	129.47
chr18	44126859	44127021	LOXHD1	58.47	81.20	66.28	73.46	103.74	59.79	77.66	127.27	101.50	117.91	91.72	57.67	75.96	84.72
chr18	44137320	44137452	LOXHD1	43.21	46.28	39.41	53.74	64.16	31.52	63.40	82.09	53.17	110.41	54.34	27.03	47.99	55.73
chr18	44139412	44139565	LOXHD1	70.31	82.68	50.71	66.96	106.03	69.69	66.15	110.79	100.98	177.64	78.94	57.96	62.99	86.57
chr18	44140047	44140508	LOXHD1	35.82	59.15	33.50	46.51	68.16	40.05	51.58	88.70	69.64	104.67	54.06	39.46	56.16	57.61
chr18	44143029	44143188	LOXHD1	24.17	36.79	37.73	33.99	39.96	21.01	36.62	50.95	41.49	48.78	32.34	27.07	36.36	35.91
chr18	44146221	44146412	LOXHD1	32.92	29.72	28.02	35.42	60.70	29.17	39.30	56.52	38.14	111.13	39.59	24.49	42.99	43.76
chr18	44149406	44149601	LOXHD1	31.60	52.50	32.59	32.08	41.47	30.52	37.13	85.72	60.21	43.86	45.41	29.53	41.31	43.55
chr18	44152050	44152125	LOXHD1	52.93	52.45	53.84	64.38	77.63	57.08	66.78	110.51	70.22	115.28	65.14	49.28	63.39	69.63
chr18	44157671	44157830	LOXHD1	43.21	46.93	34.98	40.23	86.29	42.23	47.34	69.30	54.19	95.51	44.16	36.07	58.29	53.37
chr18	44159594	44159747	LOXHD1	43.18	40.32	23.23	32.63	42.50	21.34	34.95	68.01	46.90	58.05	42.98	23.54	36.10	39.80
chr18	44171897	44172031	LOXHD1	38.00	88.36	58.81	48.58	90.04	51.03	82.49	143.25	96.75	85.60	67.85	61.90	75.82	76.06
chr18	44172462	44172547	LOXHD1	104.16	125.01	66.10	105.28	126.01	87.38	161.10	197.01	167.78	104.13	99.21	86.42	82.58	119.13
chr18	44173564	44173723	LOXHD1	50.98	71.84	39.51	50.54	58.58	39.76	77.68	97.12	80.35	56.33	62.79	40.14	53.17	60.47
chr18	44174295	44174429	LOXHD1	27.64	27.97	22.90	17.47	37.90	15.98	36.24	50.59	30.74	33.95	33.33	20.45	24.22	29.60
chr18	44181181	44181430	LOXHD1	48.98	66.13	44.02	55.58	87.79	50.86	79.23	80.04	75.48	120.40	58.01	39.71	54.37	67.19
chr18	44184070	44184192	LOXHD1	40.39	48.08	34.66	39.44	64.96	36.83	46.26	73.37	64.36	51.95	53.92	42.47	47.53	49.72
chr18	44190740	44190887	LOXHD1	34.08	55.19	45.27	45.51	57.40	33.57	61.12	66.26	62.13	68.30	42.96	38.32	43.98	50.84
chr18	44198156	44198253	LOXHD1	49.74	83.19	50.55	50.56	70.79	55.19	93.71	102.62	108.15	30.10	62.06	71.88	56.56	69.05
chr18	44219580	44219763	LOXHD1	43.21	57.10	42.81	55.62	85.73	58.10	60.11	104.69	64.58	118.06	69.04	28.30	54.80	65.61
chr18	44221920	44221999	LOXHD1	79.86	85.74	49.46	65.83	103.40	73.85	114.98	156.00	99.04	110.90	79.90	62.44	94.19	90.12
chr18	44229119	44229232	LOXHD1	25.95	30.36	14.75	17.58	39.99	20.52	32.35	43.05	28.90	74.23	23.46	14.84	29.91	30.50
chr18	44236868	44236996	LOXHD1	29.65	47.31	28.32	47.64	74.22	37.87	52.40	68.51	51.75	109.47	37.38	33.62	38.84	51.51
chr19	3585597	3585820	GIPC3	17.32	23.61	13.16	20.16	26.59	18.23	21.70	33.97	24.76	38.74	24.42	14.63	22.68	23.11
chr19	3586494	3586678	GIPC3	79.08	84.48	79.43	82.56	175.15	74.67	106.48	120.71	106.00	182.59	94.94	63.36	102.46	104.12
chr19	3586813	3586992	GIPC3	89.73	122.56	91.15	116.77	164.62	94.66	128.23	169.21	130.73	242.69	99.27	75.11	110.24	127.06
chr19	3589442	3589553	GIPC3	48.65	61.71	61.54	65.63	61.54	111.71	73.60	93.51	65.78	69.13	56.22	42.63	42.79	67.64
chr19	3589830	3589910	GIPC3	49.47	40.28	34.27	44.84	70.02	31.63	46.93	61.38	49.78	123.06	41.11	34.49	54.48	52.27
chr19	3590038	3590188	GIPC3	33.97	38.07	27.03	46.06	77.11	26.58	36.77	55.23	35.28	111.56	35.21	24.03	54.60	45.57
chr19	10334521	10335623	S1PR2	74.35	101.11	67.89	95.06	135.51	73.57	96.12	145.98	102.40	219.14	90.07	68.73	103.02	105.83
chr19	36494240	36494422	SYNE4	49.28	80.39	46.28	56.38	99.87	41.84	79.43	120.58	107.72	106.70	65.67	52.32	66.17	75.54
chr19	36494516	36494573	SYNE4	22.71	27.40	19.50	25.86	38.97	18.74	24.10	35.78	28.03	34.10	18.45	18.76	24.03	26.03
chr19	36496236	36496339	SYNE4	67.87	103.85	63.71	66.24	118.84	58.52	107.33	136.25	130.88	169.22	100.32	65.38	82.10	99.03
chr19	36497326	36497573	SYNE4	29.77	29.90	17.12	26.90	47.43	24.07	34.94	43.01	32.96	65.62	30.92	16.08	24.21	33.23
chr19	36497653	36497846	SYNE4	43.42	49.36	28.53	33.72	68.64	35.23	51.49	57.75	39.45	98.66	50.04	26.82	45.15	48.59
chr19	36498028	36498170	SYNE4	20.33	22.21	8.15	21.01	43.64	18.29	16.30	36.22	26.13	57.70	19.70	13.62	15.45	25.28
chr19	36499120	36499269	SYNE4	16.41	23.95	12.73	22.05	35.07	20.84	35.15	39.67	28.91	43.73	20.49	20.00	28.13	26.58
chr19	36499457	36499583	SYNE4	26.57	24.24	12.40	24.72	49.64	16.85	24.41	32.30	30.80	78.39	27.18	19.50	28.14	30.58
chr19	45204632	45204763	CEACAM16	32.24	31.10	27.46	36.73	64.20	32.17	37.07	56.59	52.27	94.46	39.02	21.52	42.46	43.74
chr19	45206620	45206963	CEACAM16	32.06	35.29	36.22	41.79	69.40	36.23	45.19	54.98	48.33	101.91	42.73	28.67	50.32	47.73
chr19	45207289	45207566	CEACAM16	28.04	34.68	16.52	35.74	60.58	24.12	31.42	39.85	41.38	72.86	35.40	15.86	37.80	36.37
chr19	45208861	45209138	CEACAM16	72.55	82.03	69.52	87.39	122.87	73.83	91.51	125.78	97.73	187.09	87.72	63.38	94.04	96.78
chr19	45211134	45211459	CEACAM16	64.31	54.74	55.55	61.31	119.10	57.90	69.10	94.85	72.35	163.41	61.17	51.83	72.32	77.14
chr19	45213769	45213778	CEACAM16	64.10	74.30	46.40	70.90	123.30	71.30	113.40	109.00	79.00	153.60	74.90	58.10	71.00	86.53
chr19	50713621	50714027	MYH14	67.49	84.26	56.29	57.05	128.19	60.64	79.61	108.78	90.90	156.31	61.98	51.63	79.21	83.59
chr19	50720873	50721028	MYH14	82.08	92.40	57.48	88.92	149.90	76.37	104.38	142.65	93.37	200.96	111.55	64.54	98.45	105.38
chr19	50726341	50726367	MYH14	67.11	70.59	61.56	72.33	92.70	61.11	88.15	103.74	70.41	127.04	62.19	47.74	90.48	77.06
chr19	50726505	50726606	MYH14	74.37	82.02	67.62	69.11	120.33	76.73	95.99	126.03	95.08	164.42	85.75	62.90	101.30	93.36
chr19	50727412	50727434	MYH14	61.83	65.87	39.65	42.91	79.04	47.30	75.09	85.74	83.04	112.74	53.00	40.22	66.17	65.54
chr19	50728843	50728934	MYH14	36.00	46.26	25.91	48.39	66.67	40.71	50.33	65.70	58.59	112.73	48.23	34.55	42.88	52.84
chr19	50730161	50730223	MYH14	85.90	111.78	85.81	104.08	167.06	76.86	139.87	192.54	160.21	248.10	107.87	73.79	123.05	129.49
chr19	50733783	50733880	MYH14	23.18	34.16	19.68	20.59	46.87	24.58	37.09	55.51	40.06	76.33	31.70	27.40	35.03	36.43
chr19	50735188	50735327	MYH14	60.10	78.26	58.84	90.88	134.81	61.27	80.34	122.10	89.71	180.66	96.09	61.48	87.86	92.88
chr19	50747500	50747594	MYH14	62.58	76.22	53.79	72.57	106.79	51.17	65.38	104.68	82.07	145.98	87.76	46.03	66.41	79.59
chr19	50750262	50750379	MYH14	57.92	76.09	39.87	64.39	98.08	48.18	65.67	77.90	87.47	145.25	68.65	42.97	65.44	72.70
chr19	50752245	50752396	MYH14	41.64	54.62	37.52	41.31	82.52	46.59	69.67	83.54	76.95	117.43	72.26	51.43	69.44	64.62
chr19	50752908	50753080	MYH14	43.46	40.14	28.69	54.99	67.58	31.80	40.02	62.73	50.19	121.56	46.93	18.02	50.55	50.51
chr19	50753773	50753945	MYH14	54.75	81.29	43.13	56.70	110.68	51.26	75.98	110.32	87.08	138.02	64.83	41.68	72.38	76.31
chr19	50755897	50756010	MYH14	66.13	87.51	70.67	87.55	129.20	80.45	95.02	133.82	110.54	171.10	85.75	80.13	83.50	99.82
chr19	50758478	50758575	MYH14	22.54	33.19	27.81	25.34	50.76	23.58	35.38	46.80	45.02	58.87	37.47	19.27	37.12	35.50
chr19	50760557	50760743	MYH14	93.55	114.86	93.05	84.36	121.69	84.86	97.39	140.68	133.24	232.74	95.69	69.12	109.30	113.43
chr19	50762402	50762522	MYH14	41.82	49.28	43.29	42.82	76.21	37.84	60.48	72.34	50.98	98.17	43.40	42.79	55.86	54.95
chr19	50763904	50763972	MYH14	115.17	119.62	87.25	146.30	191.90	78.00	131.09	204.13	131.52	296.22	146.16	74.54	156.13	143.49
chr19	50764733	50764892	MYH14	28.36	31.56	19.19	29.69	48.24	14.59	27.94	41.84	34.56	81.07	33.68	17.57	28.46	34.02
chr19	50766570	50766677	MYH14	24.22	29.15	21.14	21.41	49.88	15.52	25.19	36.87	24.68	61.07	33.33	21.84	32.50	30.36
chr19	50770135	50770265	MYH14	27.16	30.98	27.93	24.68	47.87	24.63	34.82	48.21	37.85	59.37	35.60	20.89	36.45	35.00
chr19	50771419	50771624	MYH14	10.34	12.57	7.94	10.72	14.68	10.76	9.28	15.66	16.73	33.97	6.67	8.69	20.30	13.17
chr19	50774667	50774803	MYH14	17.22	12.70	18.57	19.12	18.73	16.26	19.44	24.66	24.13	46.26	11.10	13.85	22.47	20.17
chr19	50775107	50775229	MYH14	24.96	27.49	11.41	30.91	31.69	18.03	17.89	25.33	19.27	59.46	22.33	15.13	25.59	25.33
chr19	50775818	50775988	MYH14	6.07	16.00	6.32	5.06	11.93	9.18	15.59	11.42	11.64	21.98	15.27	3.12	8.03	11.13
chr19	50779249	50779460	MYH14	17.32	24.51	13.48	26.02	24.22	13.06	18.66	25.96	20.60	47.76	16.60	19.22	18.46	22.28
chr19	50780015	50780158	MYH14	19.65	29.26	21.35	20.44	31.56	20.24	22.92	35.93	28.38	33.28	22.43	12.36	21.95	24.82
chr19	50781341	50781546	MYH14	27.70	36.72	20.45	25.40	42.29	24.69	36.98	50.40	40.12	53.83	32.04	21.78	32.33	34.37
chr19	50783295	50783398	MYH14	119.20	127.60	93.89	131.97	181.80	102.81	123.58	192.11	111.27	277.39	145.70	69.59	152.40	139.74
chr19	50783489	50783640	MYH14	22.74	26.01	17.11	20.69	35.89	12.59	21.94	34.43	25.83	59.96	17.63	8.31	25.33	25.26
chr19	50784852	50785099	MYH14	41.47	46.56	31.20	44.35	68.22	30.25	48.39	57.38	67.67	94.11	41.36	32.04	48.63	50.25
chr19	50789740	50789951	MYH14	45.37	70.70	45.92	60.16	76.68	35.68	68.29	91.53	78.58	105.98	50.57	46.42	61.29	64.66
chr19	50792694	50792905	MYH14	51.42	42.18	26.92	48.54	63.35	42.30	46.81	68.33	52.36	129.75	49.02	36.77	55.79	54.81
chr19	50794145	50794305	MYH14	29.70	27.14	22.07	28.49	37.29	19.11	34.42	32.93	35.03	49.68	26.35	19.50	26.85	30.14
chr19	50795519	50795646	MYH14	19.63	31.41	21.84	29.70	41.78	25.78	29.84	53.83	51.80	64.63	28.41	21.34	39.11	35.00

chr19	50796491	50796578	MYH14	66.81	93.51	52.24	71.32	91.68	52.57	74.08	126.47	82.27	178.06	75.01	53.78	74.19	84.82
chr19	50796822	50796944	MYH14	14.32	21.44	14.55	24.40	24.07	11.54	20.83	20.41	21.74	41.37	24.21	10.80	20.75	20.81
chr19	50804919	50805126	MYH14	32.46	39.54	26.71	41.41	45.52	30.66	55.66	65.81	50.26	61.27	30.09	29.50	35.76	42.41
chr19	50810307	50810414	MYH14	15.50	27.74	20.31	21.41	34.61	19.10	28.50	39.78	23.93	46.58	16.83	18.50	22.55	26.07
chr19	50812263	50812434	MYH14	41.02	58.94	44.34	54.69	87.60	33.59	56.93	89.31	66.76	113.76	52.41	34.62	61.06	61.16
chr19	50812898	50813047	MYH14	42.82	44.92	23.90	36.22	64.59	24.13	42.40	50.16	55.60	90.67	52.98	25.47	44.11	46.15
chr20	60831114	60831277	OSBPL2	53.88	77.05	42.64	39.85	58.16	31.66	66.79	98.09	65.72	32.31	44.57	27.15	50.07	53.16
chr20	60835038	60835181	OSBPL2	78.19	112.05	77.11	93.13	127.15	69.60	123.81	152.41	130.40	90.34	99.82	27.69	83.85	101.81
chr20	60838673	60838747	OSBPL2	68.23	77.29	53.53	76.32	101.23	60.45	61.43	119.47	87.16	165.77	85.27	45.12	87.27	83.44
chr20	60839686	60839774	OSBPL2	0.00	0.00	0.00	0.43	0.00	0.82	0.00	0.00	0.00	0.00	1.00	0.00	0.00	0.19
chr20	60847182	60847315	OSBPL2	83.09	86.12	63.46	95.09	154.77	63.66	93.25	115.05	107.30	204.40	97.42	51.29	122.33	101.24
chr20	60848366	60848462	OSBPL2	36.03	52.46	32.98	36.04	62.92	33.24	53.07	81.55	67.88	48.00	45.54	27.95	35.14	48.14
chr20	60854214	60854395	OSBPL2	55.65	70.33	63.05	57.58	83.33	56.17	87.49	142.03	94.49	77.31	58.63	53.31	58.28	74.95
chr20	60856115	60856221	OSBPL2	54.47	55.32	37.10	52.64	82.93	42.17	59.83	81.36	61.12	127.98	58.01	39.09	65.50	62.67
chr20	60856847	60856935	OSBPL2	70.93	113.81	64.12	74.82	130.67	82.17	142.20	193.91	130.11	115.22	90.25	70.45	97.06	106.56
chr20	60859103	60859225	OSBPL2	64.56	97.52	68.58	80.95	95.42	69.63	97.73	145.06	108.02	103.17	82.07	67.39	79.09	90.01
chr20	60861640	60861767	OSBPL2	50.92	75.35	47.30	47.89	67.94	41.05	72.46	76.15	69.66	135.34	63.58	40.95	57.24	65.72
chr20	60864264	60864386	OSBPL2	36.73	46.16	36.23	38.24	49.14	24.36	41.92	67.72	39.03	89.95	33.10	31.50	45.15	44.51
chr20	60866760	60866849	OSBPL2	41.83	68.51	31.91	48.70	79.30	42.89	75.48	77.36	80.17	100.94	47.42	47.33	61.87	61.82
chr20	60868842	60869013	OSBPL2	60.28	83.72	65.99	69.53	108.84	65.49	85.91	100.48	97.39	150.43	99.29	53.03	85.52	86.70
chr21	37833275	37834074	CLDN14	40.96	45.60	35.47	43.99	74.14	40.93	49.20	74.37	48.95	104.64	42.54	30.30	52.36	52.59
chr21	37849673	37849861	CLDN14	0.00	0.59	0.00	0.00	0.37	0.19	0.19	0.00	0.00	0.63	0.00	0.00	0.00	0.16
chr21	37882662	37882798	CLDN14	1.00	1.15	0.98	0.44	0.00	0.78	0.00	0.51	0.94	0.00	0.85	0.00	0.00	0.55
chr21	43792872	43792888	TMPRSS3	34.47	44.65	44.47	48.29	56.41	41.65	74.35	87.29	71.00	76.29	47.47	31.59	48.06	54.83
chr21	43795826	43795977	TMPRSS3	31.91	64.51	32.67	44.78	70.45	44.09	61.07	76.34	60.27	118.58	55.30	40.16	53.17	58.34
chr21	43796651	43796795	TMPRSS3	39.32	39.77	33.44	52.16	63.02	28.41	51.49	71.08	47.18	104.55	45.11	28.84	46.61	50.36
chr21	43800227	43800321	TMPRSS3	35.43	52.00	28.28	31.43	48.96	26.88	33.47	45.75	50.38	54.95	37.19	26.36	30.40	39.26
chr21	43802092	43802343	TMPRSS3	58.40	80.65	52.49	50.31	84.46	52.76	76.07	139.56	104.74	75.00	84.65	47.81	72.81	75.57
chr21	43803143	43803307	TMPRSS3	53.96	67.78	45.38	58.32	90.36	61.08	66.22	87.99	74.18	150.90	65.32	47.32	65.41	72.40
chr21	43804080	43804122	TMPRSS3	39.05	62.65	45.79	51.63	83.07	46.98	64.51	71.60	81.23	61.30	54.21	34.98	51.86	58.08
chr21	43805519	43805643	TMPRSS3	49.67	61.88	45.17	56.13	77.32	45.13	71.44	101.86	79.47	90.22	62.79	54.47	60.04	66.30
chr21	43808513	43808635	TMPRSS3	72.26	100.83	60.48	80.46	94.52	57.93	90.11	181.19	116.67	140.27	79.95	43.15	75.15	93.15
chr21	43809039	43809154	TMPRSS3	16.28	29.08	15.75	22.06	41.62	19.28	24.87	42.10	33.69	46.96	25.16	18.18	22.47	27.92
chr21	43810037	43810146	TMPRSS3	45.06	46.61	38.56	40.35	42.45	27.19	65.17	73.11	68.88	55.45	39.35	33.87	42.30	48.01
chr21	43815434	43815577	TMPRSS3	42.88	54.78	41.74	35.10	63.36	40.32	52.84	66.01	62.47	71.81	51.82	28.81	36.59	50.99
chr21	43816052	43816955	TMPRSS3	6.43	7.64	5.92	8.28	8.50	6.36	9.34	12.63	9.51	15.57	9.13	5.03	7.48	8.69
chr21	45919667	45919819	TSPEAR	18.39	29.03	18.76	22.22	42.13	20.19	30.20	29.37	29.51	58.54	24.90	17.46	28.84	28.39
chr21	45924664	45924764	TSPEAR	75.94	88.63	54.40	69.93	112.72	41.83	77.02	99.01	95.46	153.35	81.14	50.66	72.80	83.34
chr21	45929083	45929269	TSPEAR	47.50	70.10	30.60	59.34	70.34	49.99	60.88	91.08	82.57	113.74	52.22	32.73	49.41	63.43
chr21	45941767	45941995	TSPEAR	77.46	100.17	69.13	94.78	111.45	69.30	113.13	115.92	96.53	225.22	89.89	71.80	97.64	102.90
chr21	45945537	45945722	TSPEAR	52.16	72.94	47.48	62.35	93.46	53.82	76.58	115.47	86.60	120.42	72.39	54.30	63.02	75.66
chr21	45947176	45947401	TSPEAR	64.85	81.59	43.72	71.03	88.37	59.05	78.13	124.62	99.85	158.21	67.88	51.69	67.47	82.42
chr21	45948336	45948466	TSPEAR	51.08	60.92	51.27	72.71	89.31	59.11	63.88	97.51	60.15	162.18	63.14	56.50	91.34	73.98
chr21	45949682	45949837	TSPEAR	99.31	118.03	68.64	93.21	148.43	71.52	133.26	164.79	144.30	225.96	101.10	66.98	90.60	119.63
chr21	45950927	45951016	TSPEAR	65.97	86.89	43.37	63.56	95.94	63.79	85.84	94.97	75.03	162.53	73.00	48.84	72.86	79.98
chr21	45953569	45953806	TSPEAR	46.90	45.41	27.80	66.59	100.66	43.24	56.50	73.49	57.78	133.47	62.85	44.38	81.71	63.26
chr21	45987670	45987889	TSPEAR	61.43	72.20	59.28	74.62	106.95	59.65	84.70	100.21	88.94	151.68	77.57	55.51	78.22	82.73
chr21	46110461	46110521	TSPEAR	0.00	0.00	0.00	0.00	0.00	0.00	0.00	1.00	0.00	1.00	0.00	0.00	0.00	0.17
chr21	46131349	46131429	TSPEAR	61.89	85.98	52.93	75.69	116.43	58.60	79.48	123.01	99.80	161.47	69.10	68.83	85.63	87.77
chr22	36678715	36678831	MYH9	41.32	37.53	28.06	57.90	67.49	41.74	52.74	85.79	65.23	130.48	59.47	31.14	53.23	58.24
chr22	36680140	36680311	MYH9	41.99	60.03	39.40	37.38	68.42	34.70	66.13	78.98	74.02	111.35	33.88	36.56	44.53	56.90
chr22	36680450	36680557	MYH9	72.23	74.68	49.92	84.96	136.49	65.46	75.25	97.54	99.53	187.81	81.80	57.65	82.51	90.28
chr22	36681168	36681375	MYH9	35.50	48.33	31.09	43.19	66.79	29.92	51.81	79.18	51.61	100.58	44.20	29.18	56.23	50.95
chr22	36681705	36681827	MYH9	13.89	12.94	12.48	16.33	37.09	9.53	19.34	16.52	17.33	41.41	15.07	12.36	26.03	18.69
chr22	36681912	36681999	MYH9	38.69	40.67	31.73	37.88	83.77	24.43	48.61	61.67	50.01	100.47	55.88	28.06	48.55	50.16
chr22	36682765	36682892	MYH9	46.78	32.77	38.34	61.88	84.80	42.94	49.30	59.52	53.09	124.77	33.19	38.33	55.95	55.48
chr22	36684299	36684459	MYH9	36.36	59.37	32.05	54.39	63.89	46.46	50.48	49.28	57.31	118.61	66.16	36.04	64.24	55.87
chr22	36684774	36684985	MYH9	50.34	47.79	42.24	52.50	99.59	41.45	55.37	63.99	51.70	124.87	58.99	41.31	57.98	60.84
chr22	36685132	36685343	MYH9	35.84	44.33	22.32	36.21	66.46	31.09	36.79	51.75	31.17	105.85	42.19	25.09	37.86	44.09
chr22	36688033	36688280	MYH9	65.30	63.68	55.35	59.21	99.24	44.19	70.90	90.92	84.43	159.85	76.13	55.39	72.25	77.05
chr22	36689376	36689527	MYH9	44.11	42.84	27.45	52.96	73.34	32.76	56.99	53.51	43.21	106.89	40.22	34.74	46.82	50.75
chr22	36689806	36689909	MYH9	49.92	54.44	38.60	53.43	70.31	44.25	59.81	80.27	63.00	120.68	61.23	46.48	54.18	61.87
chr22	36690139	36690344	MYH9	69.99	75.60	51.83	85.72	128.70	60.43	90.90	117.75	91.32	188.28	82.56	50.95	82.89	91.17
chr22	36690979	36691122	MYH9	34.89	42.31	36.60	50.66	80.22	26.83	40.51	56.03	57.08	114.94	51.05	40.88	46.74	52.67
chr22	36691552	36691763	MYH9	18.49	27.26	19.75	33.58	42.20	19.14	27.02	34.92	30.85	48.97	22.72	18.22	28.10	28.59
chr22	36692890	36693060	MYH9	23.19	27.61	21.43	23.70	38.15	26.68	39.57	43.93	36.53	84.92	37.74	21.82	38.89	35.44
chr22	36694966	36695088	MYH9	22.88	35.98	23.03	31.85	38.48	15.11	31.59	46.94	38.73	44.87	29.24	34.90	28.69	32.80
chr22	36696174	36696310	MYH9	60.84	46.43	36.66	65.78	107.39	43.28	68.61	93.51	71.93	151.74	59.01	36.78	82.29	70.16
chr22	36696898	36697103	MYH9	49.38	40.06	30.35	44.49	62.02	34.85	42.90	70.60	53.12	105.71	46.44	32.44	50.58	51.03
chr22	36697581	36697711	MYH9	33.76	32.70	31.84	43.48	61.53	33.54	35.47	45.98	40.44	75.53	43.12	30.98	42.82	42.36
chr22	36698615	36698722	MYH9	88.94	90.46	56.62	99.81	174.55	74.46	88.17	136.24	103.17	225.76	93.15	72.97	104.42	108.69
chr22	36700042	36700201	MYH9	86.62	92.60	66.96	95.08	144.27	75.98	93.83	148.04	98.18	205.78	101.09	75.93	91.33	107.03
chr22	36701080	36701148	MYH9	45.54	52.52	34.78	54.91	78.78	42.30	55.03	67.55	65.07	64.12	50.33	34.25	47.14	53.77
chr22	36701977	36702097	MYH9	58.40	77.26	57.91	73.53	101.98	61.64	81.17	93.38	102.07	160.51	57.64	54.22	70.50	81.64
chr22	36702461	36702653	MYH9	47.02	59.79	29.90	68.90	99.17	42.48	61.86	88.31	61.39	131.78	61.28	35.91	59.25	65.65
chr22	36705328	36705441	MYH9	79.18	139.95	70.63	89.75	169.06	91.68	123.14	190.31	135.24	203.38	111.18	72.22	109.74	122.98
chr22	36708095	36708267	MYH9	54.19	66.79	26.02	69.31	82.60	39.95	54.76	71.94	61.77	149.38	59.55	38.54	70.85	64.57
chr22	36710191	36710363	MYH9	44.27	55.26	36.58	62.03	84.38	38.21	61.98	77.14	64.95	122.42	61.80	34.22	66.08	61.94
chr22	36712563	36712714	MYH9	56.73	72.39	40.79	48.89	67.82	50.07	59.14	88.43	76.52	136.29	55.52	34.01	49.09	65.55
chr22	36714253	36714370	MYH9	31.31	52.72	40.23	33.70	41.27	31.14	47.34	67.07	49.78	64.11	43.53	23.31	45.72	43.79

chr22	36715586	36715680	MYH9	98.67	123.83	96.28	119.48	179.61	75.20	129.92	140.15	135.13	213.34	122.11	98.82	117.45	127.71
chr22	36716266	36716408	MYH9	75.93	85.53	68.75	82.73	121.99	52.33	96.93	146.91	100.60	190.19	77.24	49.78	85.69	95.74
chr22	36716844	36716941	MYH9	25.62	25.31	23.46	31.98	45.38	20.20	25.05	52.61	44.16	49.14	31.19	21.94	33.54	33.00
chr22	36717804	36717866	MYH9	62.27	71.98	44.56	50.78	77.30	47.95	90.27	104.73	83.59	82.97	66.79	51.63	52.32	69.57
chr22	36718475	36718566	MYH9	53.78	63.36	45.03	65.40	86.16	45.52	64.82	74.40	78.20	140.89	41.60	41.17	69.43	66.69
chr22	36722614	36722706	MYH9	51.99	64.18	38.22	69.83	94.13	52.91	57.24	94.95	70.06	127.81	59.16	24.73	49.99	67.10
chr22	36723507	36723533	MYH9	30.93	58.59	29.41	41.04	64.52	32.07	49.70	72.33	62.89	44.30	53.07	35.00	38.00	47.82
chr22	36737416	36737571	MYH9	53.06	58.61	38.02	43.23	55.79	33.63	52.35	84.15	64.80	39.09	55.03	34.17	43.57	50.99
chr22	36744950	36745300	MYH9	86.41	109.23	97.16	101.68	139.79	87.24	126.48	175.26	146.75	214.61	108.02	87.08	123.49	123.31
chr22	38093596	38093703	TRIOBP	0.35	1.21	0.65	0.00	1.27	2.82	0.00	0.00	0.54	0.60	0.00	0.87	0.19	0.69
chr22	38097314	38097486	TRIOBP	61.27	64.61	52.22	78.83	100.19	55.92	71.88	123.82	72.79	152.37	86.75	53.99	77.86	81.22
chr22	38106435	38106573	TRIOBP	24.47	39.85	24.44	30.08	49.76	24.23	34.45	49.32	41.87	82.58	35.10	22.12	33.59	38.19
chr22	38109218	38109418	TRIOBP	28.06	48.55	21.00	33.00	65.00	27.00	35.12	45.32	41.71	93.77	41.55	26.17	46.69	42.19
chr22	38111771	38111941	TRIOBP	24.58	39.88	24.94	34.41	50.60	22.66	47.25	44.41	44.62	62.85	28.66	23.25	33.70	37.34
chr22	38119193	38122510	TRIOBP	122.99	129.17	106.63	139.20	212.20	109.54	143.33	150.31	156.38	323.76	166.82	113.17	145.35	156.13
chr22	38129306	38129419	TRIOBP	38.03	41.36	29.87	37.11	64.88	27.59	55.07	61.33	49.41	94.74	29.54	24.99	46.38	46.16
chr22	38130407	38131449	TRIOBP	67.55	93.65	62.81	77.83	130.18	59.61	86.05	103.10	110.47	186.32	78.87	59.14	86.18	92.97
chr22	38134650	38134726	TRIOBP	46.27	64.13	43.44	37.04	59.08	30.87	59.73	102.79	95.86	40.32	53.21	37.61	37.68	55.86
chr22	38136903	38137039	TRIOBP	27.61	33.27	20.18	25.92	43.46	31.12	34.60	44.82	39.26	58.81	31.31	20.53	37.78	34.24
chr22	38142246	38142427	TRIOBP	3.30	7.20	3.74	2.09	3.36	5.49	2.71	3.39	4.74	4.35	3.25	0.76	5.45	3.70
chr22	38147780	38147835	TRIOBP	36.13	56.38	23.23	49.86	75.96	32.36	51.00	86.39	58.21	114.75	41.20	32.02	49.68	54.79
chr22	38150885	38150991	TRIOBP	30.49	23.80	19.40	24.34	31.73	18.24	34.71	41.24	31.07	57.39	21.70	23.70	21.20	29.82
chr22	38151109	38151197	TRIOBP	67.08	74.10	41.48	64.37	94.06	44.49	76.74	94.29	69.15	162.85	67.22	53.38	66.49	75.77
chr22	38151558	38151666	TRIOBP	65.76	71.90	59.32	75.95	102.41	63.16	63.73	99.62	77.01	158.39	82.34	42.05	63.85	80.14
chr22	38153621	38154145	TRIOBP	38.04	66.54	35.61	49.34	84.02	28.28	51.94	70.76	64.36	123.49	46.45	34.57	55.11	57.78
chr22	38155162	38155271	TRIOBP	36.23	44.46	31.34	35.14	55.19	28.47	44.92	69.65	50.68	96.52	39.71	28.22	34.08	46.71
chr22	38155425	38155534	TRIOBP	57.26	60.92	39.26	50.54	80.00	32.73	60.78	89.57	70.23	109.03	63.04	39.27	47.65	62.72
chr22	38161678	38161824	TRIOBP	7.93	11.55	9.96	15.96	22.77	13.43	10.36	20.93	9.50	30.59	11.01	8.31	16.91	14.36
chr22	38164082	38164183	TRIOBP	59.42	46.94	43.04	46.84	89.71	46.20	60.77	95.76	74.79	132.96	70.70	52.61	75.74	68.31
chr22	38165036	38165194	TRIOBP	25.08	27.20	16.86	33.92	52.94	24.99	24.67	45.08	33.75	62.59	20.50	21.84	31.18	32.45
chr22	38165270	38165382	TRIOBP	22.13	37.33	21.35	32.09	57.19	24.44	29.20	51.99	36.28	95.99	36.37	19.81	34.44	38.68
chr22	38167658	38167743	TRIOBP	47.19	38.99	27.62	40.17	59.87	32.86	39.31	54.52	51.01	91.95	46.65	38.62	56.92	47.40
chr22	38168609	38168771	TRIOBP	56.74	66.51	48.21	65.99	96.96	55.35	67.23	102.08	64.26	189.58	65.72	33.95	77.17	76.05
chrx	21755668	21755815	SMPX	41.73	59.64	16.93	20.48	26.68	15.51	72.01	51.06	72.22	19.69	26.51	19.58	41.71	36.84
chrx	21761869	21761954	SMPX	40.76	74.72	17.77	16.15	27.59	21.74	49.70	47.64	81.97	38.43	24.48	11.49	52.08	37.70
chrx	21772365	21772420	SMPX	87.52	113.59	28.20	36.63	49.25	27.36	95.80	73.73	147.34	14.64	50.13	28.55	78.63	62.73
chrx	82763334	82764418	POU3F4	76.65	90.81	35.07	42.52	65.16	29.98	96.31	63.38	103.05	188.91	41.20	31.43	90.27	72.04
chrx	106871860	106871980	PRPS1	54.36	104.09	38.42	34.90	53.04	30.25	95.56	74.09	101.32	156.62	35.12	26.32	79.29	67.01
chrx	106882526	106882708	PRPS1	104.07	167.46	51.77	58.03	78.74	49.84	220.55	138.78	221.75	56.34	80.99	66.79	124.38	107.93
chrx	106884133	106884230	PRPS1	61.27	88.28	33.06	26.69	47.60	18.08	93.99	76.24	119.69	20.74	29.70	32.89	71.72	54.02
chrx	106885597	106885720	PRPS1	68.71	91.95	22.54	23.98	47.48	28.66	97.33	63.32	100.29	18.80	39.03	27.67	68.16	52.48
chrx	106888408	106888580	PRPS1	62.14	96.20	38.08	35.16	58.30	38.79	101.72	62.96	114.87	135.51	41.05	30.44	78.42	67.94
chrx	106890837	106890995	PRPS1	81.86	127.36	40.72	30.48	50.69	30.78	140.89	85.01	131.75	57.56	45.93	47.34	70.61	72.53
chrx	106893171	106893262	PRPS1	68.92	116.40	40.82	40.13	49.01	41.29	134.98	88.09	140.20	22.15	53.73	43.20	109.95	69.91
															**Average**		66.29

Uncovered regions are highlighted in gray.

Table S5. Variants in autosomal dominant NSHL genes detected by WES in this study

Gene Symbol	Indiv-idual	Sex	Age of onset	Nucleotide change[a]	Amino acid change	Exon (zygosity, segrega-tion)	Amino acid sequence conservation[b]	Frequencies in the dbSNP database[c]	Frequencies in the ExAC database[d]	Frequencies in the NBK database[e]	PP2[f]	MT[g]	PRO-VEAN[h]	SIFT[i]
MYO7A	YUHL 26-21	Fm	8 yr	c.1094A>G	p.Asp365Gly	11 (het, M)	*D. rerio*	ND	ND	ND	Bn (0.341)	DC (0.999)	Del (-3.39)	Tol (0.307)
DFNA5	YUHL 44-21	Ml	2 yr	c.116_119del AAAA	p.Lys39Argfs*24	2 (het, M)	NA	rs773853744 (MAF: -=0.00003/4)	ND	ND	NA	DC (1.000)	NA	NA

Abbreviations are as follows: Bn, benign; Dam, damaging; DC, disease causing; Del, deleterious; F, heterozygous mutation identified in father; Fm, female; het, heterozygous in affected individual; M, heterozygous mutation identified in mother; MAF, minor allele frequency; Ml, male; NA, not applicable; ND, no data or DNA available; Neu, neutral; PD, probably damaging; PM, Polymorphism; PP2, PolyPhen-2 prediction score Humvar; PROVEAN, Protein Variation Effect Analyzer; SIFT, Sorting Intolerant from Tolerant; SNP, single nucleotide polymorphism; Tol, tolerant; yr, years.

[a]cDNA mutations are numbered according to human cDNA reference sequence NM_000260.3 (*MYO7A*), NM_004403.2 (*DFNA5*); +1 corresponds to the A of ATG translation initiation codon. [b]Amino acid residue is continually conserved throughout evolution including the species as indicated. [c]dbSNP database (http://www.ncbi.nlm.nih.gov/SNP). [d]ExAC browser (http://exac.broadinstitute.org/). [e]National Biobank of Korea, the Centers for Disease Control and Prevention. [f]PolyPhen-2 prediction score HumVar ranges from 0 to 1.0; 0 = benign, 1.0 = probably damaging (http://genetics.bwh.harvard.edu/pph2/). [g]Mutation taster (http://www.mutationtaster.org/). [h]PROVEAN (http://provean.jcvi.org/index.php). [h]SIFT (http://sift.jcvi.org/).

Table S6. Heterozygous variants in autosomal recessive NSHL genes detected by WES in this study

Individual	Gene Symbol	Sex	Age of onset	Nucleotide change[a]	Amino acid change	Exon	Amino acid sequence conservation[b]	Frequencies in the dbSNP database[c]	Frequencies in the ExAC database[d]	Frequencies in the NBK database[e]	PP2[f]	MT[g]	PRO-VEAN[h]	SIFT
**YUHL 13-21**	*LOXHD1*	Ml	1 yr	c.3185C>T	p.Ser1062Leu	20	*D. rerio*	ND	ND	ND	PD (0.993)	DC (0.672)	Del (-4.43)	Dam (0.004)
	GIPC3	Ml	1 yr	c.58C>T	p.Pro20Ser	1	*M. musculus*	ND	ND	ND	Bn (0.003)	DC (0.945)	Neu (-0.21)	Tol (0.249)
**YUHL 20-21**	*LOXHD1*	Ml	2 yr	c.1810-2A>T	NA	13 (intron)	ND	ND	ND	A:0.00125945	NA	DC (1.000)	NA	NA
	GRXCR2	Ml	2 yr	c.88C>T	p.Arg30*	1	ND	ND	ND	ND	NA	DC (1.000)	NA	NA
**YUHL 14-21**	*PCDH15*	Fm	5 yr	c.4703A>G	p.Lys1568Arg	35	*X. tropicalis*	rs774523472 (C=0.00002/3)	3/120774 (no hom)	ND	Bn (0.149)	PM (0.956)	Neu (-0.18)	Dam (0.023)
**YUHL 26-21**	*MYO3A*	Fm	8 yr	c.2902G>A	p.Val968Ile	26	*D. rerio*	rs747578242 (A=0.000008/1)	1/121398 (no hom)	ND	PD (0.971)	DC (0.999)	Neu (-0.82)	Dam (0.045)
**YUHL 36-21**	*TSPEAR*	Fm	4 yr	c.1144A>G	p.Lys382Glu	7	*D. rerio*	rs782103811 (C=0.000008/1)	1/121366 (no hom)	C:0.00251889	Bn (0.225)	PM (0.908)	Neu (-1.10)	Tol (0.297)
**YUHL 44-21**	*KARS*	Ml	2 yr	c.1450T>G	p.Cys484Gly	12	*D. rerio*	rs772704062 (C=0.000008/1)	1/121136 (no hom)	ND	PD (0.989)	DC (0.999)	Del (-9.11)	Dam (0.01)
	FAM65B	Ml	2 yr	c.3176G>A	p.Arg1059His	23	*M. musculus*	rs753906236 (T=0.00009/2)	2/21828 (no hom)	ND	Bn (0.023)	DC (0.992)	Neu (-0.85)	Tol (0.1)

Abbreviations are as follows: Bn, benign; Dam, damaging; DC, disease causing; Del, deleterious; F, heterozygous mutation identified in father; Fm, female; het, heterozygous in affected individual; M, heterozygous mutation identified in mother; MAF, minor allele frequency; Ml, male; mo, month; NA, not applicable; ND, no data or DNA available; Neu, neutral; PD, probably damaging; PM, Polymorphism; PP2, PolyPhen-2 prediction score Humvar; PROVEAN, Protein Variation Effect Analyzer; SIFT, Sorting Intolerant from Tolerant; SNP, single nucleotide polymorphism; Tol, tolerant; yr, years.

[a]cDNA mutations are numbered according to human cDNA reference sequence NM_144612.6 (*LOXHD1*), NM_133261.2 (*GIPC3*), NM_001142763.1 (*PCDH15*), NM_001080516.1 (*GRXCR2*), NM_017433.4 (*MYO3A*), NM_144991.2 (TSPEAR), NM_001130089.1 (*KARS*), NM_014722.3 (*FAM65B*); +1 corresponds to the A of ATG translation initiation codon. [b]Amino acid residue is continually conserved throughout evolution including the species as indicated. [c]dbSNP database (http://www.ncbi.nlm.nih.gov/SNP). [d]ExAC browser (http://exac.broadinstitute.org/). [e]National Biobank of Korea, the Centers for Disease Control and Prevention. [f]PolyPhen-2 prediction score HumVar ranges from 0 to 1.0; 0 = benign, 1.0 = probably damaging (http://genetics.bwh.harvard.edu/pph2/). [g]Mutation taster (http://www.mutationtaster.org/). [h]PROVEAN (http://provean.jcvi.org/index.php). [h]SIFT (http://sift.jcvi.org/).

Table S7. Candidate genes identified in 10 affected individuals in whom a causative mutation in 72 known NSHL genes is excluded.

	Mutation type	Gene symbol	Alteration (phys. Location)	Accession #	cDNA position	Amino acid substitution	Count	Coverage	Conservation				dbSNP	EXAC Population Frequencies	EVS	NBK Allele frequency	Mutation Taster	PP2 Humvar	PROVEAN	SIFT	OMIM
									Mm	Gg	Xt	Dr									
YUHL 10-21	Compound Het	CSMD3	chr8:113662447C>T	NM_198123	c.3136G>A	p.Glu1046Lys	25	54	E	E	D	E	rs368870291 (NA)	3/121404 (no hom)	TT=0/TC=1/CC=6502	No	DC (0.9999)	Possibly Damaging (0.728)	Del (-3.30)	Tol (0.068)	No
			chr8:114111156C>T	NM_198123	c.746G>A	p.Ser249Asn	51	110	S	S	S	S	rs73701331 (T=0.0094/47)	304/120722 (2 hom)	TT=2/TC=103/CC=6398	T:0.00125945	DC (0.9257)	Possibly Damaging (0.985)	Neu (-1.48)	Tol (0.146)	No
	Compound Het	SPATC1	chr8:145094836G>C	NM_198572	c.238G>C	p.Val80Leu	32	62	A				rs191614050 (C=0.0020/10)	137/120552 (2 hom)	No	C:0.0214106	PM (0.9995)	Benign (0.001)	Neu (-0.50)	Tol (0.063)	No
			chr8:145095523T>A	NM_198572	c.821T>A	p.Met274Lys	68	129					No	3/117988 (no hom)	No	A:0.00125945	PM (0.9999)	Benign (0.022)	Neu (-1.32)	Tol (0.071)	No
	De novo	PITRM1	chr10:3212294A>G	NM_001242307.1	c.159+2T>C		19	41					No	No	No	No	DC (1)	No	No	No	No
	De novo	TYW5	chr2:200803702A>C	NM_001039693.2	c.440T>G	p.Val147Gly	15	27	V	V	V	V	No	No	No	No	DC (0.999)	Probably Damaging (0.997)	Del (-5.62)	Dam (0)	No
	De novo	OLFM4	chr13:53624689T>C	NM_006418.4	c.1316T>C	p.Val439Ala	45	83	V	V	V	V	No	No	No	No	DC (0.999)	Probably Damaging (0.996)	Del (-3.95)	Dam (0.003)	No
	De novo	RALGAPA1	chr14:36104800A>G	NM_194301.3	c.4163T>C	p.Val1388Ala	8	14	V	V	V	V	No	No	No	No	DC (0.999)	Probably Damaging (0.923)	Del (-2.9)	Dam (0.004)	No
	De novo	C14orf37	chr14:58563541G>A	NM_001001872.3	c.1990C>T	p.Gln664*	25	63	Q	Q	Q		No	No	No	No	DC (0.999)	No	No	No	No
	De novo	CORO2B	chr15:68987480C>T	NM_006091.4	c.218C>T	p.Thr73Ile	30	68	T	T	T	F	No	No	No	No	DC (0.997)	Possibly Damaging (0.464)	Del (-5.08)	Dam (0.004)	No
	De novo	AHNAK	chr11:62295239T>A	NM_001620.2	c.6650A>T	p.Asp2217Val	49	95	V	E	K		No	No	No	No	PM (0.738)	Possibly Damaging (0.809)	Del (-5.88)	Dam (0.032)	No
	De novo	RARRES3	chr11:63312273T>C	NM_004585.4	c.299T>C	p.Val100Ala	28	58	I		I	L	No	No	No	No	PM (0.999)	Possibly Damaging (0.851)	Del (-3.7)	Dam (0.003)	No
YUHL 14-21	Compound Het	KDELC2	chr11:108352754G>A	NM_153705	c.880C>T	p.Leu294Phe	29	56	L	L	L	L	rs75859794 (A=0.0006/3)	16/120542 (no hom)	No	A:0.00377834	DC (0.999)	Possibly Damaging (0.534)	Del (-2.97)	Dam (0.043)	No
			chr11:108356955G>A	NM_153705	c.613C>T	p.Arg205Trp	19	35	R	R	R	R	rs778413475 (NA)	6/120752 (no hom)	No	No	DC (0.999)	Probably Damaging (1.000)	Del (-6.27)	Dam (0.011)	No
	Compound Het	ARHGEF18	chr19:7506873C>T	NM_001130955	c.731C>T	p.Ala244Val	18	40	S	A	A	A	rs200966278 (T=0.0004/2)	23/121366 (no hom)	No	No	PM (0.999)	Benign (0.017)	Neu (-0.80)	Tol (1.00)	No
			chr19:7523482G>A	NM_001130955	c.1702G>A	p.Ala568Thr	27	57	A	A	A	A	rs370503507 (NA)	8/121388 (no hom)	No	No	DC (0.999)	Possbily Damaging (0.765)	Del (-3.04)	Dam (0.014)	No
	Compound Het	GPR151	chr5:145895095..145895099de CACAG	NM_194251	c.578_582delCTGTG	p.Ala193Glyfs*2	32	72	V		H	H	rs751873529 (NA)	No	No	No	DC (1.000)	No	No	No	No
			chr5:145895102..145895105de GGTA	NM_194251	c.572_575delTACC	p.Val191Glufs*59	32	71	P		P	P	rs762221581 (NA)	No	No	No	DC (1.000)	No	No	No	No
	Compound Het	DAB2IP	chr9:124329505A>G	NM_032552	c.38A>G	p.Tyr13Cys	31	56	Y	N			No	No	No	No	PM (0.991)	Benign (0.000)	Neu (-0.86)	Dam (0.02)	No
			chr9:124535604G>A	NM_032552	c.2713G>A	p.Gly905Ser	9	18	G		G	G	rs201889776 (A=0.0006/3)	5/11046 (no hom)	No	No	DC (0.999)	Probably Damaging (0.949)	Neu (-1.17)	Tol (0.138)	No
YUHL 20-21	De novo	IDE	chr10:94230099A>C	NM_004969.3	c.2120T>G	p.Val707Gly	62	109	V	V	V	V	No	No	No	No	DC (0.999)	possibly damaging (0.799)	Del (-4.89)	Dam (0.001)	No
	De novo	MYO1B	chr2:192234274T>C	NM_001130158.2	c.1039T>C	p.Tyr347His	11	23	Y	Y		Y	No	No	No	No	DC (0.999)	Probably Damaging (0.997)	Del (-4.39)	Dam (0.011)	No
	De novo	WDR75	chr2:190306288T>G	NM_032168.2	c.70T>G	p.Phe24Val	44	89	F		F	I	No	No	No	No	DC (0.999)	Possibly Damaging (0.515)	Del (-4.23)	Dam (0.007)	No
	De novo	FAM198A	chr3:43097017G>A	NM_001129908.2	c.1507G>A	p.Gly503Ser	17	35	G		G		No	No	No	No	DC (0.999)	Probably Damaging (1)	Del (-5.66)	Dam (0)	No
	De novo	WDR49	chr3:167250724C>T	NM_178824.3	c.940G>A	p.Glu314Lys	46	82	E		E		No	No	No	No	DC (0.912)	possibly damaging (0.78)	Del (-2.95)	Dam (0.006)	No
	De novo	FXYD1	chr19:35632062C>T	NM_005031.4	c.121C>T	p.Leu41Phe	48	88	L				No	No	No	No	DC (0.999)	probably damaging (0.999)	Del (-3.65)	Dam (0.003)	No
YUHL 21-21	Compound Het	OBSCN	chr1:228399851G>A	NM_001271223	c.367G>A	p.Glu123Lys	44	82	E	L	L	L	rs377006803 (A=0.0007/7)	7/10664 (no hom)	AA=0/AG=1/GG=4340	No	PM (0.999)	Possibly Damaging (0.725)	Neu (-0.63)	Tol (0.646)	No
			chr1:228471318G>A	NM_001271223	c.8852G>A	p.Gly2951Asp	92	188	G	G	G	G	rs184062354 (A=0.00005/6)	6/119754 (no hom)	No	No	PM (0.999)	Probably Damaging (0.935)	Del (-3.89)	Tol (0.230)	No
YUH 25-21	Hemi	SPANXC	chrx:140335705..140335706AG >TC	NM_022661.3	c.238_239delCTinsG A	p.Leu80Asp	11	14					No	No	No	No	PM (0.999)	Benign (0.007)	Del (-4.45)	Tol (0.107)	No
YUHL 26-21	De novo	TMEM87A	chr15:42510532..42510534del AGA	NM_015497.4	c.1582_1584delTCT	p.Ser528del	34	75	S	S	S	S	No	No	No	No	DC (0.999)	No	Del (-11.89)	No	No
	De novo	SLC24A6	chr12:113758672G>A	NM_024959.3	c.403C>T	p.Leu135Phe	34	63	L	L	L	L	No	No	No	No	DC (0.999)	Probably Damaging (0.999)	Del (-3.05)	Dam (0.025)	No
	De novo	MYH7B	chr20:33575448G>C	NM_020884.4	c.1362G>C	p.Glu454Asp	40	86	E	E	E	E	No	No	No	No	DC (0.998)	Probably Damaging (0.971)	Del (-2.58)	Dam (0.005)	No
	De novo	COL24A1	chr1:86249806delG	NM_152890.5	c.4211delC	p.Pro1404Glnfs* 36	73	133	P	P	P	Q	No	No	No	No	DC (1)	No	No	No	No

	De novo	NOP16	chr5:175812232..175812233TC>AT	NM_001256539.3	c.382_383delGAinsAT	p.Glu128Met	22	43	E	E	E		No	No	No	No	DC (0.999)	Probably Damaging (0.991)	Del (-6.19)	Dam (0.001)	No
	De novo	COL21A1	chr6:56021708G>A	NM_030820.3	c.1411C>T	p.Gln471*	43	88			Q		No	No	No	No	DC (1)	No	No	No	No
	De novo	IL20RA	chr6:137330474T>A	NM_014432.3	c.559A>T	p.Asn187Tyr	74	153	N	K			No	No	No	No	PM (0.997)	Probably Damaging (0.987)	Del (-6.78)	Dam (0.003)	No
YUHL 36-21	De novo	CTNND2	chr5:11159721G>A	NM_001332.3	c.2126C>T	p.Ser709Leu	28	46	S	S	S	S	No	No	No	No	DC (0.999)	Probably Damaging (0.991)	Del (-4.55)	Dam (0.002)	No
	De novo	NCOR2	chr12:124824618A>G	NM_006312.5	c.5621T>C	p.Val1874Ala	19	39	V	V	V	V	No	No	No	No	DC (0.999)	Probably Damaging (0.984)	Del (-3.8)	Dam (0)	No
	De novo	C2CD2	chr21:43338337C>G	NM_015500.1	c.598-1G>C		19	35					No	No	No	No	DC (0.999)	No	No	No	No
	De novo	TMEM192	chr4:166021908C>T	NM_001100389.1	c.311G>A	p.Trp104*	75	148	W	W	W		No	No	No	No	DC (1)	No	No	No	No
	De novo	SLC44A5	chr1:75669500T>C	NM_152697.4	c.2066A>G	p.Asn689Ser	40	80	N	W	N	N	No	No	No	No	DC (0.999)	Probably Damaging (0.987)	Del (-4.94)	Dam (0)	No
	De novo	PRSS3	chr9:33797856A>G	NM_007343.3	c.401A>G	p.Asn134Ser	57	92		N	N	N	No	No	No	No	DC (0.999)	Possibly Damaging (0.631)	Del (-4.27)	Dam (0.006)	No
	De novo	ZNF823	chr19:11832671A>G	NM_001080493.3	c.1678T>C	p.Cys560Arg	71	188		C	C	C	No	No	No	No	DC (0.998)	Probably Damaging (0.999)	Del (-9.38)	Dam (0)	No
	De novo	DNAH8	chr6:38794121T>C	NM_001206927.1	c.4037T>C	p.Ile1346Thr	31	74	I	I			No	No	No	No	DC (0.999)	Possibly Damaging (0.821)	Del (-4.18)	Dam (0.001)	No
	De novo	GPLD1	chr6:24476461A>G	NM_001503.3	c.278T>C	p.Leu93Pro	31	53	L		L		No	No	No	No	DC (0.999)	Probably Damaging (1)	Del (-5.17)	Dam (0.001)	No
	De novo	ARHGEF11	chr1:156941593A>G	NM_198236.2	c.598T>C	p.Tyr200His	24	42	Y		Y		No	No	No	No	PM (0.578)	Possibly Damaging (0.861)	Del (-2.52)	Dam (0.016)	No
	De novo	MEDAG	chr13:31480737G>T	NM_032849.3	c.85G>T	p.Asp29Tyr	18	34	D				No	No	No	No	DC (0.809)	Probably Damaging (0.984)	Del (-3.93)	Dam (0.006)	No
	De novo	OR4N5	chr14:20612394C>T	NM_001004724.1	c.500C>T	p.Pro167Leu	50	96		D	T	S	No	No	No	No	DC (0.990)	Probably Damaging (1)	Del (-9.87)	Dam (0)	No
	De novo	ZNF208	chr19:22171636T>A	NM_007153.3	c.79A>T	p.Asn27Tyr	165	320					No	No	No	No	PM (0.999)	Probably Damaging (0.916)	Del (-3.93)	Dam (0.011)	No
YUHL 40-21	De novo	ANKRD13A	chr12:110461907A>G	NM_033121.1	c.791A>G	p.Tyr264Cys	11	25	Y	Y	Y	Y	No	No	No	No	DC (0.999)	Probably Damaging (0.969)	Del (-7.56)	Dam (0.001)	No
	De novo	SUMF2	chr7:56141878G>A	NM_015411.2	c.408G>A	p.Trp136*	19	35	W	W	W	W	No	No	No	No	DC (1)	No	No	No	No
	De novo	ZNF652	chr17:47388672A>G	NM_001145365.1	c.1309+2T>C		14	40					No	No	No	No	DC (1)	No	No	No	No
	De novo	PMS1	chr2:190718725^190718726insGTA	NM_000534.4	c.883_884insGTA	p.Phe295delinsCysIle	11	22	F	F	F		No	No	No	No	PM (0.999)	No	Del (-9.53)	No	No
	De novo	KIAA1549	chr7:138583842G>C	NM_001164665.1	c.3706C>G	p.Pro1236Ala	36	71	P	P	P	P	No	No	No	No	DC (0.999)	Probably Damaging (1)	Del (-7.58)	Dam (0.003)	No
	De novo	TOR2A	chr9:130494926C>T	NM_001085347.2	c.638G>A	p.Trp213*	33	68	W	W	W	R	No	No	No	No	DC (1)	No	No	No	No
	De novo	SVEP1	chr9:113137662G>T	NM_153366.3	c.10586C>A	p.Ser3529Tyr	29	68	S	S	S	T	No	No	No	No	DC (0.999)	Possibly Damaging (0.837)	Del (-3.3)	Dam (0.004)	No
	De novo	GRM3	chr7:86394915A>G	NM_000840.2	c.454A>G	p.Ser152Gly	48	87	S	S	D	S	No	No	No	No	DC (0.999)	Possibly Damaging (0.641)	Del (-2.99)	Dam (0.033)	No
YUHL 43-21	De novo	CCNI	chr4:77976319A>C	NM_006835.2	c.674T>G	p.Leu225Arg	32	64	L	L	L	L	No	No	No	No	DC (0.999)	Probably Damaging (0.988)	Del (-5.36)	Dam (0)	No
	De novo	FAM172A	chr5:93217372A>G	NM_032042.5	c.590T>C	p.Leu197Pro	15	26	L	L	L	L	No	No	No	No	DC (0.999)	Probably Damaging (0.985)	Del (-4.87)	Dam (0.024)	No
	De novo	C11orf31	chr11:57509638..57509639delAT	NM_170746.3	c.318_319delAT	p.Lys106Asnfs*3	8	18	K	K			No	No	No	No	DC (1)	No	No	No	No
	De novo	PARP14	chr3:122420433^122420434insG	NM_017554.2	c.3032_3033insG	p.Gly1013Argfs*15	24	54	P		K		No	No	No	No	DC (1)	No	No	No	No
	De novo	MELTF	chr3:196743107C>T	NM_005929.5	c.1034G>A	p.Gly345Asp	18	40	G	G	G		No	No	No	No	DC (0.999)	Probably Damaging (0.998)	Del (-6.84)	Dam (0)	No
	De novo	SLC27A6	chr5:128301847^128301848insAACA	NM_001017372.2	c.17_18insAACA	p.Val8Apnfs*24	18	31	L	V	I	I	No	No	No	No	DC (1)	No	No	No	No
	De novo	ELMO3	chr16:67233603^67233604insC	NM_024712.3	c.295_296insC	p.Glu100*	25	34	P	A	S	P	No	No	No	No	DC (1)	No	No	No	No
	De novo	OAS2	chr12:113445732C>A	NM_016817.2	c.1879C>A	p.Gln627Lys	20	37	Q				No	No	No	No	PM (0.527)	Probably Damaging (0.957)	Del (-3.91)	Dam (0.005)	No
	Compound Het	HEATR1	chr1:236740100C>T	NM_018072.5	c.2905G>A	p.Asp969Asn	18	37	D	D	D	D	rs141424510 (T=0.0002/20)	20/120224 (no hom)	No	No	DC (0.999)	Probably Damaging (0.442)	Del (-4.18)	Tol (0.311)	No
			chr1:236762924C>T	NM_018072.5	c.360G>A		35	78					rs200399414 (T=0.00002/3)	3/120376 (no hom)	No	No	DC (1)	No	Neu (0)	Tol (1)	No
	Hemi	SLC25A14	chrx:129505596G>A	NM_001282197.1	c.998G>A	p.Arg333Gln	13	13	R		R	R	No	No	No	No	No	Probably Damaging (0.972)	Del (-3.43)	Dam (0)	No

YUHL 44-21	Hemi	SSX3	chrx:48213531T>C	NM_021014.3	c.185-2A>G		50	50					No	No	No	No	PM (0.989)	No	No	No	No
	De novo	CAND2	chr3:12858190G>A	NM_001162499.1	c.1759G>A	p.Val587Met	22	49	V	V	V	V	No	No	No	No	DC (0.999)	Probably Damaging (0.997)	Del (-2.88)	Dam (0)	No
	De novo	DOPEY2	chr21:37653848G>C	NM_005128.3	c.6099G>C	p.Gln2033His	34	76	Q	Q	Q	Q	No	No	No	No	DC (0.999)	Probably Damaging (0.998)	Del (-2.8)	Dam (0.008)	No
	De novo	ADAMTSL3	chr15:84506875C>G	NM_207517.2	c.635C>G	p.Ala212Gly	17	40	A	A	A	A	No	No	No	No	DC (0.999)	Probably Damaging (0.994)	Del (-2.88)	Dam (0)	No
	De novo	SWI5	chr9:131050954A>G	NM_001040011.1	c.667A>G	p.Lys223Glu	21	36	K				No	No	No	No	DC (0.961)	Probably Damaging (0.975)	Del (-2.8)	Dam (0.012)	No
	De novo	CEND1	chr11:788449T>C	NM_016564.3	c.128A>G	p.Glu43Gly	6	16	D		D		No	No	No	No	DC (0.819)	Possibly Damaging (0.466)	Del (-4.83)	Dam (0.005)	No

Table S8. Number of copy number variation detected by EXCAVATOR and ExomeDepth

#	Family ID	EXCAVATOR		ExomeDepth	
		CNV	biallelic deletions	CNV	biallelic deletions
1	YUHL8	111	4	168	3
2	YUHL10	138	5	185	8
3	YUHL13	142	9	185	8
4	YUHL14	99	5	181	7
5	YUHL20	144	3	184	10
6	YUHL21	95	2	113	1
7	YUHL24	112	10	148	5
8	YUHL25	132	8	121	5
9	YUHL26	121	9	190	9
10	YUHL36	112	5	176	9
11	YUHL40	148	12	151	10
12	YUHL43	139	9	114	5
13	YUHL44	144	6	135	6
**Average**		125.92	6.69	157.77	6.62
